# Carbene Addition and Its Remote Influence on Dy···Dy Coupling, Relaxation of Magnetization, and Magnetic Frustration in Fullerene Single-Molecule Magnets

**DOI:** 10.1021/jacsau.5c01106

**Published:** 2025-11-25

**Authors:** Matheus Felipe de Souza Barbosa, Wei Yang, Noel Israel, Fupin Liu, Bernd Büchner, Stanislav M. Avdoshenko, Alexey A. Popov

**Affiliations:** a Leibniz Institute for Solid State and Materials Research (IFW Dresden), Helmholtzstr. 20, Dresden 01069, Germany; b Jiangsu Key Laboratory of New Power Batteries, Jiangsu Collaborative Innovation Center of Biomedical Functional Materials, School of Chemistry and Materials Science, 12534 Nanjing Normal University, Nanjing 210023 China

**Keywords:** metallofullerene, carbene addition, single-molecule magnet, exchange interactions, spin−lattice relaxation

## Abstract

Magnetic properties of lanthanide endohedral metallofullerenes are strongly modulated by intramolecular metal–metal interactions, which suppress the quantum tunneling of magnetization (QTM) in Dy_2_ScN@C_80_, but lead to magnetic frustration with pronounced QTM in Dy_3_N@C_80_. In this work, we explore how exohedral chemical modification of Dy_2_ScN@C_80_ and Dy_3_N@C_80_ by photochemical addition of adamantylidene (Ad) affects Dy···Dy interactions and influences their single-molecule magnetism. For each fullerene, the photochemical reaction with adamantane aziridine produced two isomers of Ad monoadduct, minor [5,6]-open and major [6,6]-open. By virtue of the high sensitivity of the ^1^H nuclear spin probe in the Ad moiety to the position of Dy ions, paramagnetic NMR helped to establish Sc-Ad coordination in the [5,6] isomer and predominant Dy-Ad coordination in the [6,6] isomer of Dy_2_ScN@C_80_(Ad). SQUID magnetometry and relaxation measurements demonstrated that Ad addition has almost no effect on the strength of the Dy···Dy coupling in the [6,6] isomer of Dy_2_ScN@C_80_(Ad), but it does increase the coupling in the [5,6] counterpart by 20%. The blocking temperature of magnetization and the coercivity are both softened by adamantylidene addition, irrespective of the isomeric structure of Dy_2_ScN@C_80_(Ad). For Dy_3_N@C_80_(Ad), Ad addition substantially increased Dy···Dy coupling constants and the energy spread of exchange-coupled states in comparison to that of Dy_3_N@C_80_ and lifted geometric frustration. As a result, both Dy_3_N@C_80_(Ad) isomers exhibit open hysteresis without pronounced QTM signatures and have a higher blocking temperature of magnetization than the pristine Dy_3_N@C_80_. Our work demonstrates that chemical derivatization can have profound influence on the metal–metal coupling and relaxation of magnetization in metallofullerene molecular magnets.

## Graphical abstract

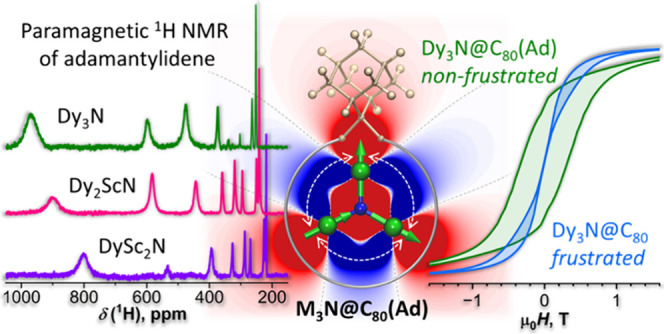

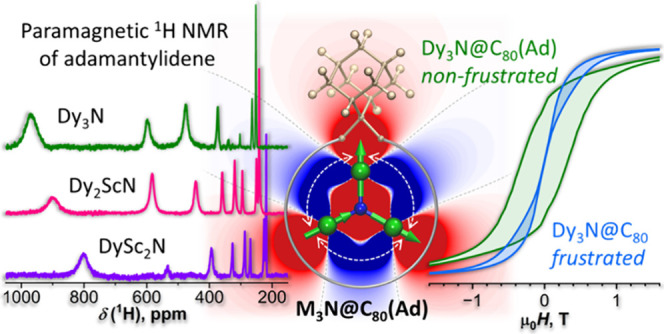

## Introduction

Molecular magnetism of multinuclear complexes is determined by an intricate interplay of single-ion anisotropy and intramolecular magnetic interactions between metal ions.^1–5^ In endohedral metallofullerenes (EMFs), carbon cage can host several magnetic lanthanide ions in close proximity to each other.^6–11^ Interactions between them are often mediated by one or two nonmetal ions, such as the oxide O^2–^,^12–16^ sulfide S^2–^,^17–19^ acetylide C_2_^2–^,^13,19^ or nitride N^3–^,^20–27^ to name a few. This confinement results, on the one hand, in the large single-ion anisotropy for lanthanides,^21,28,29^ and, on the other hand, in unusually strong exchange and dipolar interactions in endohedral species.^12,13,17^

The role of interactions is very visible in the evolution of the single-molecule magnetism (SMM) in the series of nitride clusterfullerenes Dy*_*x*_* Sc_3–*x*_N@*I* *_h_*-C_80_ (*x* = 1–3) as the number of Dy ions increases from 1 to 3.^20,22,23,30^ In all of these molecules, Dy ions experience a strong axial ligand field (LF) imposed by the central nitride ion. Thus, Dy magnetic moments are aligned along Dy–N bonds, and their magnetic ground-state doublet with *J* *_*z*_* = ± 15/2 is separated from higher-energy states by hundreds cm^–1^.^20,21,28,31^ But despite the very similar single-ion properties, a very different SMM behavior is observed for these molecules. Single-ion DySc_2_N@C_80_ shows magnetic hysteresis with a pronounced zero-field quantum tunneling of magnetization (QTM).^23,30^ In Dy_2_ScN@C_80_, ferromagnetic Dy···Dy coupling suppresses zero-field QTM, leading to a large remanence and coercivity. Dy_2_ScN@C_80_ also showed a high Orbach relaxation barrier of 1200 cm^–1^, corresponding to the fourth excited state in the LF-split ^6^H_15/2_ multiplet.^20^ In Dy_3_N@C_80_, a triangular arrangement of Dy ions results in geometric frustration and a highly degenerate sextet ground state.^22,28^ Zero-field QTM is again allowed, resulting in closing of the hysteresis in zero field and narrow opening at higher fields.^22^ Dy_3_N@C_80_ has the lowest blocking temperature of magnetization in the series. Thus, Dy···Dy coupling is responsible for the strong variation of the SMM properties in otherwise similar molecules. It is the goal of this work to understand if these interactions can be tuned by chemical derivatization of the fullerene cage.

Metallofullerenes have rich exohedral chemistry,^32–36^ particularly in the form of various cycloaddition reactions, which are often used for making functional EMF derivatives.^37–49^ But chemical functionalization of fullerenes is noninnocent from the point of view of endohedral species and their physical properties. Cycloaddition introduces a local perturbation in the fullerene π-system, which also affects how endohedral metal atoms interact with it and their internal dynamics inside the cage. For instance, metals tend to avoid modified fragments of the cage for some addition types but appear attracted to the addition site in other types of derivatives. [2 + 1] carbene addition, such as the photochemical reaction with adamantane diaziridine (Figure 1a), is a well-known example of the latter type.^50–56^ When attaching to the fullerene cage in M_3_N@*I* *_h_*-C_80_, the adamantylidene (Ad) group opens one cage σ-bond and usually forms two regioisomers, by the number of C–C bond types in the *I* *_h_*-C_80_ cage (Figure 1).^52^ In both [5,6]-open and [6,6]-open adducts, one of the metal atoms of the endohedral cluster is coordinated to the Ad-addition site (Figure 1b).

**Figure 1:**
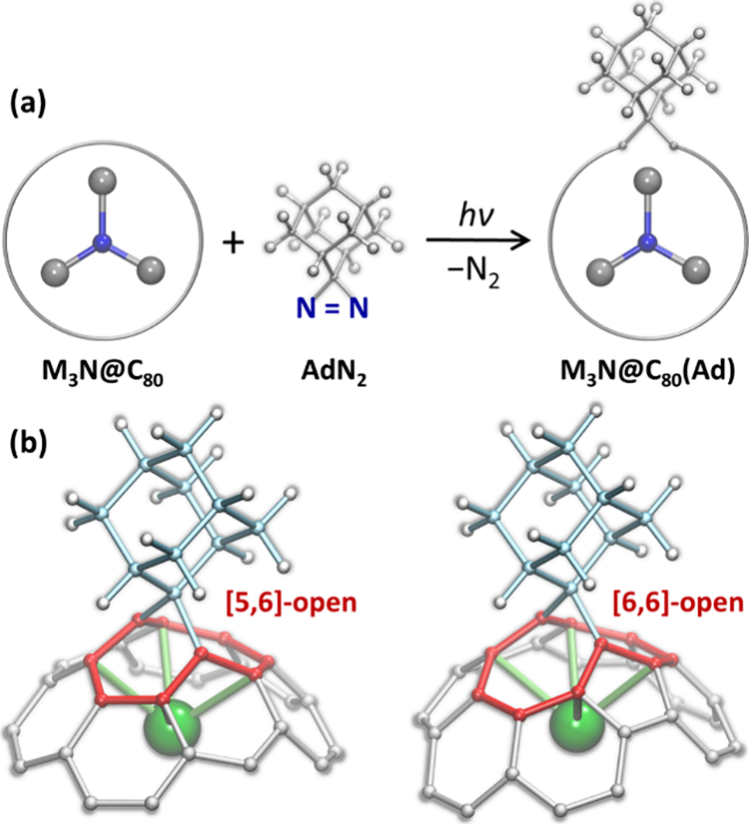
(a) Photochemical reaction between nitride clusterfullerene M_3_N@C_80_ and adamantane aziridine AdN_2_ with formation of the M_3_N@C_80_(Ad) monoadduct. (b) Fragments of [5,6]-open and [6,6]-open Ad monoadducts of M_3_N@*I* *_h_*-C_80_; pentagon/hexagon at the [5,6]-open edge and two hexagons at the [6,6]-open edge are highlighted in red, and endohedral metal coordinated to the Ad-addition site is shown as a green sphere.

Recently, we employed addition of adamantylidene to DySc_2_N@C_80_ and NdSc_2_N@C_80_ to study how chemical modification affects the single-ion anisotropy of lanthanide ions and related properties, such as the single-molecule magnetism.^57^ The isomeric structure of the adduct was found to strongly affect how the cluster coordinates to the Ad-addition site. Coordination via Sc appeared prevalent for the [5,6] isomer, while the coexistence of both lanthanide and Sc coordination modes was found for the [6,6] isomer. This had a decisive influence on the SMM properties, since the ^Ad^Dy coordination reduced the axiality of Dy^3+^ and decreased the blocking temperature of magnetization in comparison to the nonfunctionalized DySc_2_N@C_80_, whereas the ^Ad^Sc coordination enhanced the ligand-field splitting of Dy^3+^ and increased the blocking temperature. Thus, chemical modification can be used to fine-tune the magnetic properties of lanthanide EMFs by modifying their single-ion anisotropy. In this work, we shift the focus from the single-ion properties to Dy···Dy interactions and study how exohedral derivatization of the carbon cage can affect them in di- and trinuclear systems Dy_2_ScN@C_80_ and Dy_3_N@C_80_. We first analyze how composition of the Dy*_*x*_* Sc_3–*x*_N cluster (*x* = 1–3) influences the regioisomerism of carbene addition, determine molecular structures of the adducts by paramagnetic NMR and DFT calculations, and then proceed to the analysis of the static and dynamic magnetic properties of the derivatives by the SQUID magnetometry with assistance of magnetic simulations employing effective spin Hamiltonian formalism with parameters from ab initio calculations.

## Synthesis of M_3_N@C_80_(Ad) Adducts

Toluene solutions of Dy*_*x*_* Sc_3–*x*_N@*I* *_h_*-C_80_ (*x* = 0–3) mixed with excess of AdN_2_ were irradiated by a UV-LED light source (λ = 365 nm) to induce the photochemical addition of Ad to fullerenes (Figure 1a). Since we only studied M_3_N@C_80_ fullerenes with the *I* *_h_*-C_80_ cage isomer in this work, hereafter, we will omit the designation of the cage symmetry. Figure 2a shows the HPLC chromatograms measured at certain moments during the reaction. Formation of Dy*_*x*_* Sc_3–*x*_N@C_80_(Ad) monoadducts is evidenced by appearance of the double peak with twice shorter retention time than that of the pristine Dy*_*x*_* Sc_3–*x*_N@C_80_. Monoadducts can further react by adding another Ad moiety and forming bis-adducts Dy*_*x*_* Sc_3–*x*_N@C_80_(Ad)_2_, which appear in chromatograms at even shorter times (twice shorter than the monoadduct). Bis-adducts were found to form many regioisomers,^52^ which complicates their separation and strongly reduces isolable yields of individual end-products. Therefore, we preferred to stop irradiation when the amount of bis-adducts was still relatively low, even though conversion of Dy*_*x*_* Sc_3–*x*_N@C_80_ was not complete (Figure 2a). As follows from the irradiation time depicted in Figure 2a, the reactivity in the Dy*_*x*_* Sc_3–*x*_N@C_80_ series increases with the number of Dy ions in the cluster.

**Figure 2:**
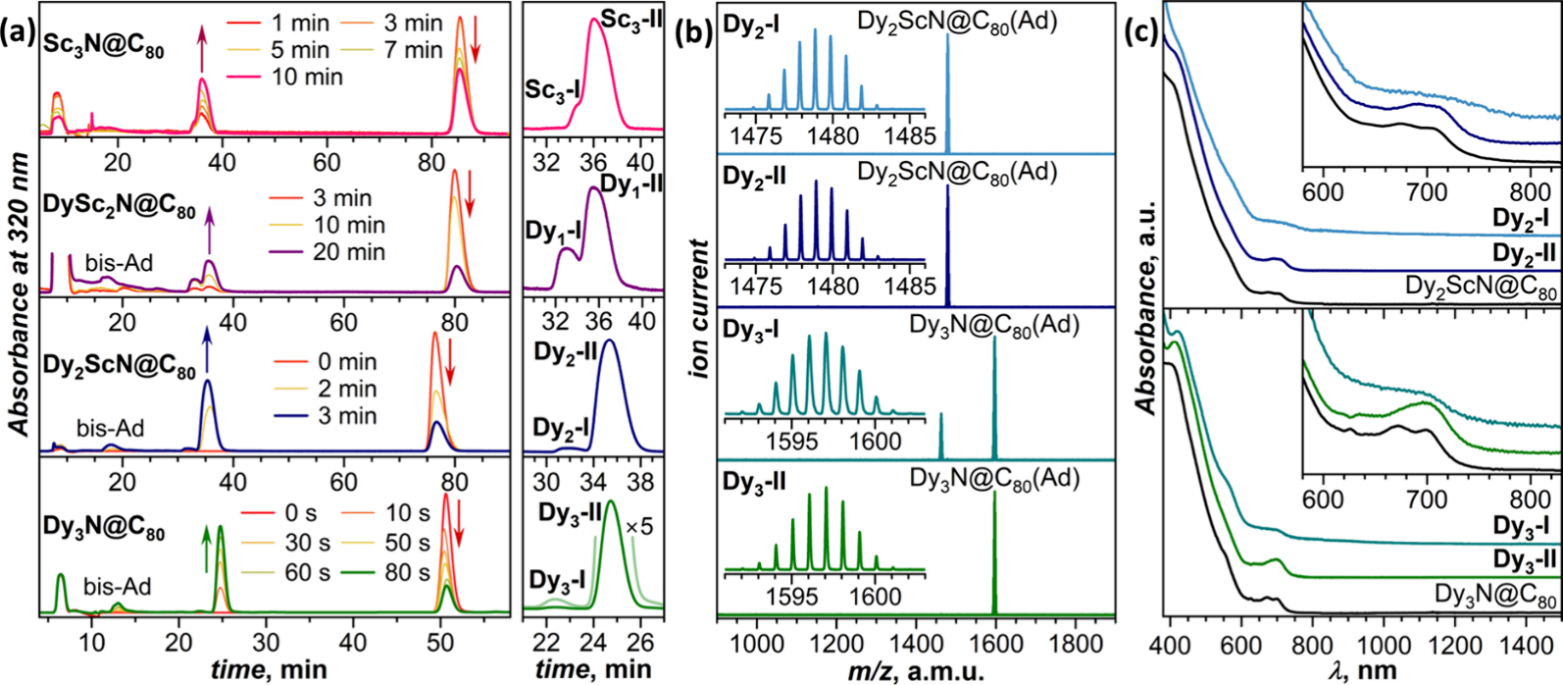
(a) HPLC traces measured at different moments of time during the photochemical reaction between AdN_2_ and Dy_*x*_Sc_3–*x*_N@C_80_ (*x* = 0–3); right panels show magnification of the region with two isomers of Dy_*x*_Sc_3–*x*_N@C_80_(Ad) monoadducts denoted as **Dy_*x*_-I** and **Dy_*x*_-II** (**Sc_3_-I** and **Sc_3_-II** for Sc_3_N@C_80_(Ad)). HPLC conditions: Buckyprep column, toluene as eluent, elution rate of 2 mL min^–1^ (Sc_3_N, DySc_2_N, and Dy_2_ScN), and 5 mL min^–1^ (Dy_3_N, two Buckyprep column). (b) MALDI mass spectra of separated Dy_2_ScN@C_80_(Ad) and Dy_3_N@C_80_(Ad) isomers, insets show isotopic distribution for molecular peaks; negative ion mode, TPBD (1,1,4,4 tetraphenyl-1,3-butadiene) as a matrix. (c) Vis-NIR absorption spectra of separated Dy_2_ScN@C_80_(Ad) and Dy_3_N@C_80_(Ad) isomers in CS_2_ solution compared to the spectra of pristine Dy_2_ScN@C_80_ and Dy_3_N@C_80_.

The double HPLC peak of the monoadduct is caused by two regioisomers with the Ad group attached across the [5,6] or [6,6] C–C bond (Figure 1b, Figure S1), which will be hereafter labeled as **Dy** *_**x**_* **-I** for [5,6]-open Dy*_*x*_* Sc_3–*x*_N@C_80_(Ad) with shorter retention time and **Dy** *_**x**_* **-II** for [6,6]-open Dy*_*x*_* Sc_3–*x*_N@C_80_(Ad) eluting later. Pure **Dy** **_2_** **-I**, **Dy** **_2_** **-II**, **Dy** **_3_** **-I**, and **Dy** **_3_** **-II** were obtained by HPLC separation of corresponding fractions (see Figure S2 for additional details) and characterized by matrix-assisted laser-desorption ionization (MALDI) mass-spectrometry with time-of-fight detection (Figure 2b). M_3_N@C_80_(Ad) adducts are usually stable under MALDI conditions and produce only molecular peaks, but for **Dy** **_3_** **-I**, we also observed fragmentation to Dy_3_N@C_80_. Since Ad addition does not change the π-system of the fullerene cage, absorption spectra of Dy_2_ScN@C_80_(Ad) and Dy_3_N@C_80_(Ad) are similar to those of nonderivatized fullerenes (Figure 2c). However, there is a subtle but visible difference between the spectra in that absorption features of [5,6] isomers are more smeared than those of [6,6] congeners.

[6,6] isomers are formed in higher yield than [5,6] for all M_3_N@C_80_ studied so far, but the ratio of the isomers varies in the large range and strongly depends on the composition of the M_3_N cluster.^52,57^ The size of the cluster (quantified as the sum of Shannon’s ionic radii^58^ of three metals) appears to be one of the major factors, and the [6,6]:[5,6] ratio increases from 6:1 for Sc_3_N to 80:1 for Dy_3_N. We also performed reactions with Lu_3_N@C_80_ and Y_3_N@C_80_ and found that they follow this trend (see Figures S3–S5 for further details). Importantly, our results on Sc_3_N@C_80_ and Lu_3_N@C_80_ are very close to the earlier report by Yamada et al.,^52^ indicating that the isomer ratio is stable against certain variation of reaction conditions. For M_3_N@C_80_ with asymmetric mixed metal clusters, the relative yield of [5,6] is increased, as follows from the numbers for DySc_2_N (2.8:1), NdSc_2_N (1.6:1), and to some extent Dy_2_ScN (26:1). DFT calculations discussed below demonstrate that [5,6] isomers of M_3_N@C_80_(Ad) are less stable than [6,6] isomers for all cluster compositions (Table 1), and the relative energy of the [5,6] isomer increases with the cluster size in an almost linear fashion (Figure 3). Thus, relative yields and relative energies do not simply follow each other, suggesting that the yield of isomers is probably controlled by kinetic factors. Note that the 1,3-dipolar cycloaddition (Prato reaction) and Diels–Alder addition to M_3_N@C_80_ were also found to depend on the cluster size, the [6,6] isomer being more abundant for larger clusters.^59–63^

**Table 1:** Sum of Metal Ionic Radii in M_3_N,^a^ Ratio of the Yields for [6,6] and [5,6] Isomer of M_3_N@C_80_(Ad),^b^ and DFT-Computed Relative Energies^c^

adduct	Σ*R*_*i*_^3+^, Å	ratio I: II	Δ*E* ([5,6]), kJ mol^–1^
Sc_3_N@C_80_	2.24	1:5.3 (1:6.2)^52^	4.5
DySc_2_N@C_80_	2.40	1:2.8 ^57^	14.5
NdSc_2_N@C_80_	2.47	1:1.6 ^57^	12.8
Dy_2_ScN@C_80_	2.57	1:26	16.8
Lu_3_N@C_80_	2.58	1:20 (1:22)^52^	15.8
Y_3_N@C_80_	2.70	1:50	20.7
Dy_3_N@C_80_	2.74	1:80	21.6

a Sum of Shannon’s ionic radii for VI-coordinated metal ions.

b The ratio of isomers is calculated from HPLC peak areas.

c PBE-D/PAW level with 4f-in-core pseudopotential for lanthanides using VASP code.^64,65^

**Figure 3:**
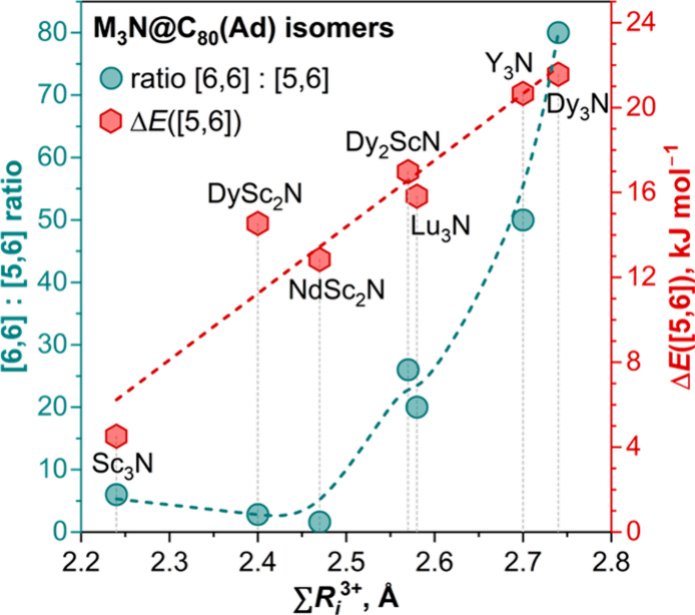
Experimental ratio of [6,6] and [5,6] isomers of M_3_N@C_80_(Ad) (scale on the left, dark cyan dots) and DFT-computed relative energies of [5,6] isomers versus more stable [6,6] isomers (scale on the right, red hexagons) plotted versus the sum of Shannon’s ionic radii of metals in M_3_N clusters. Dashed lines are shown to guide the eye only.

## Molecular Structures of Dy_2_ScN@C_80_(Ad) and Dy_3_N@C_80_(Ad)

### Paramagnetic ^1^H NMR

Isomeric structures of M_3_N@C_80_(Ad) adducts can be distinguished by ^1^H NMR spectroscopy since [6,6] and [5,6] adducts should give 8 and 9 proton signals, respectively. Furthermore, a dipolar magnetic field produced by lanthanide ions shifts the resonance positions of nearby nuclei, which can help to determine how an endohedral cluster is coordinated inside the cage. For a molecule with a single lanthanide ion in solution, pseudocontact shift of the *i*th proton (δ_*i*_^pc^) can be presented as a product of magnetic anisotropy and geometry factors:^66^

$$\delta_{i}^{\mathrm{pc}}=\frac{3\cos^{2}\theta_{i}-1}{12\pi R_{i}^{3}}\left(\chi_{zz}-\frac{\chi_{xx}+\chi_{yy}}{2}\right)$$ 1

where χ_*zz*_ and χ*_xx,yy_* are the longitudinal and transversal principal components of the magnetic susceptibility tensor, respectively, *R*_*i*_ is the distance between lanthanide atom and proton, and θ_*i*_ is the angle between quantization axis (*z* principal axis of the χ tensor) of the lanthanide ion and radius vector *R*_*i*_. When χ_*xx*_ ≠ χ_*yy*_, an additional term needs to be added to the formula to account for rhombicity, but our calculations described in the Supporting Information showed that this term is insignificant in Dy*_*x*_* Sc_3–*x*_N@C_80_(Ad), and in the following discussion, we rely on eq 1. Since pseudocontact shifts reflect both the magnetic anisotropy of lanthanide ion and its position in the molecule with respect to the probed nuclei, paramagnetic NMR became a valuable tool in the studies of molecular magnets.^57,67–78^

The DySc_2_N cluster in DySc_2_N@C_80_(Ad) can adopt two coordination modes, with Sc atom pointing toward the Ad-addition site (^Ad^Sc) and with the Dy atom in that position (^Ad^Dy) (Figure 4a). Because of the substantial difference in the geometric term, the ^Ad^Dy form of DySc_2_N@C_80_(Ad) has 10-fold stronger paramagnetic shifts of Ad protons than ^Ad^Sc, which allows their straightforward discrimination. Our recent study demonstrated the coexistence of both forms for **Dy** **_1_** **-II** (Figure 4b) and the dominance of the ^Ad^Sc form for **Dy** **_1_** **-I** (Figure S6).^57^ For a weakly interacting multinuclear system, a pseudocontact shift can be presented as a sum over individual contributions of lanthanide ions. As exchange and dipolar Dy···Dy interactions in nitride clusterfullerenes do not exceed several cm^–1^ (vide infra), they should be nonessential at room temperature, and Dy ions can be treated as noninteracting. The spatial distributions of the geometry terms in Dy_2_ScN@C_80_(Ad) and Dy_3_N@C_80_(Ad) are visualized in Figure 4a. The Dy_2_ScN cluster can also adopt ^Ad^Dy or ^Ad^Sc configurations, while the Dy_3_N cluster obviously has only the ^Ad^Dy form.

**Figure 4:**
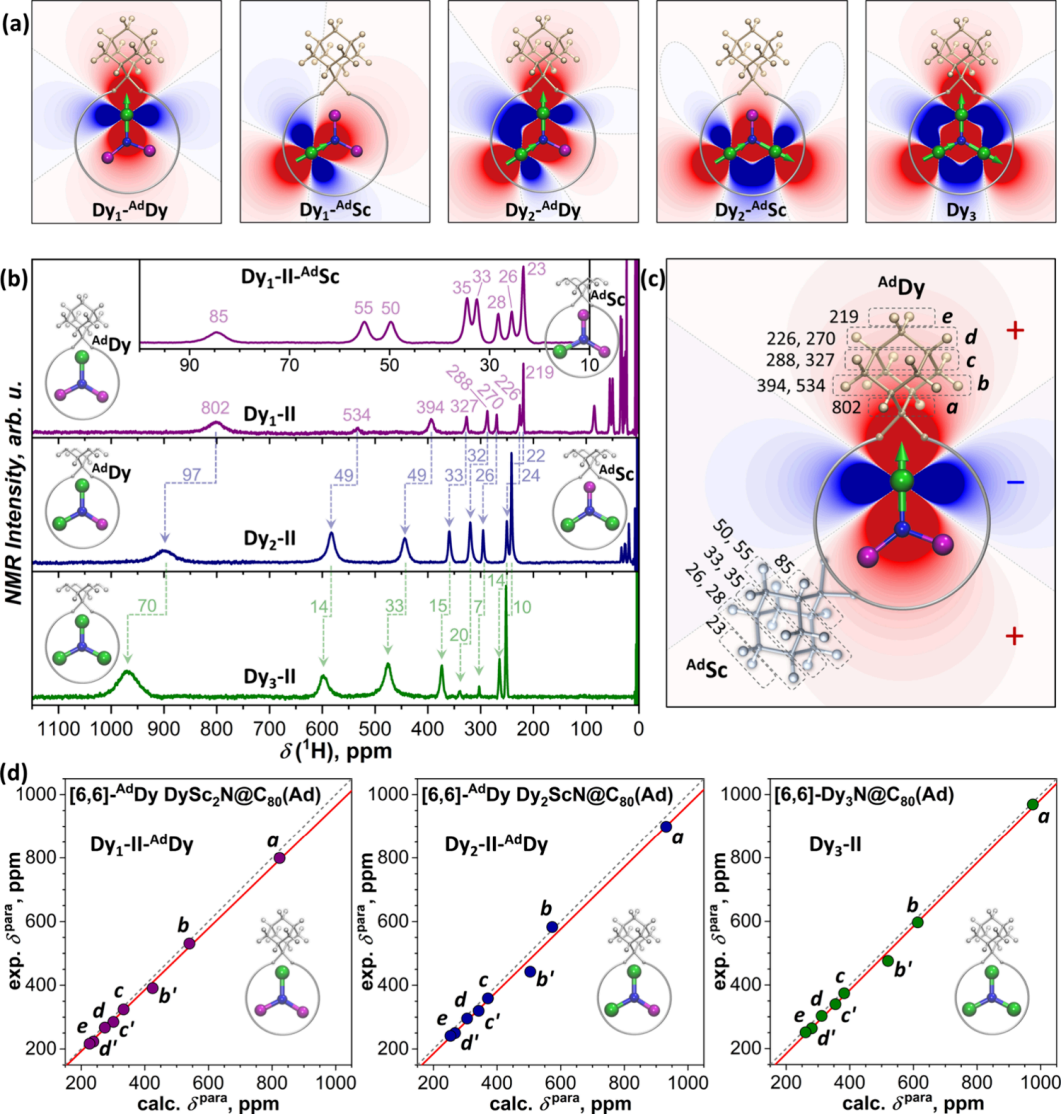
(a) Spatial distribution of the geometrical factor from eq 1 in the plane of the metal-nitride cluster for DySc_2_N@C_80_(Ad), Dy_2_ScN@C_80_(Ad), and Dy_3_N@C_80_(Ad) (red – positive, blue – negative); orientations of quantization axes for each Dy are shown as green arrows; for di- and trinuclear systems, we assume that all Dy ions have identical single-ion anisotropy. (b) ^1^H NMR spectra of **Dy_1_-II**, **Dy_2_-II**, and **Dy_3_-II** (*T* = 298 K, CS_2_ solution), inset shows the enlarged spectrum of the ^Ad^Sc form of **Dy_1_-II**; note that due to the very broad excitation bandwidth and inevitable variation of the phase correction, relative intensities of the peaks are not well comparable. (c) Spatial distribution of the function (3cos^2^θ – 1)/*R* ^3^ around Dy ion in the DySc_2_N cluster overlaid with schematic structures of ^Ad^Dy and ^Ad^Sc forms of DySc_2_N@C_80_(Ad) and ^1^H chemical shifts in the Ad moiety for each form; also shown is the labeling of proton types (***a*−*e***). (d) Correlation between experimental and calculated paramagnetic chemical shifts in ^Ad^Dy forms of **Dy_1_-II**, **Dy_2_-II**, and **Dy_3_-II**; dashed lines are plots of δ^exp^ = δ^calc^ and are shown to guide the eye, red lines are linear fits (see the Supporting Information for more details on calculations and correlations between experimental and calculated shifts).

Figure 4b compares the ^1^H NMR spectra of **Dy** **_1_** **-II**, **Dy** **_2_** **-II**, and **Dy** **_3_** **-II**. All three compounds exhibit eight peaks at 200–1000 ppm, a characteristic for the ^Ad^Dy form with the Ad moiety in the [6,6] position. In accordance with the additivity conjecture, ^Ad^Dy chemical shifts increase with the number of Dy atoms in the nitride cluster. If the contribution of Ad-coordinated Dy is assumed identical in all three compounds, the incremental increase should be caused by one or two Dy ions distant to the Ad site, and increments should be similar to the chemical shifts in the ^Ad^Sc form of **Dy** **_1_** **-II** (see inset in Figure 4b and numerical values in Figure 4c). After diamagnetic contributions of 2–3 ppm are considered, the increase of the ^Ad^Dy chemical shifts from **Dy** **_1_** **-II** to **Dy** **_2_** **-II** almost exactly matches the chemical shifts of the ^Ad^Sc form of **Dy** **_1_** **-II**, but the increase from **Dy** **_2_** **-II** to **Dy** **_3_** **-II** is considerably smaller than might be expected for a simple additivity scheme. We then computed paramagnetic chemical shifts using eq 1, for which atomic coordinates were obtained from DFT calculations, whereas principal orientations and components of the χ tensor at 298 K were computed ab initio with the CASSCF/RASSI-SO method. Excellent agreement between computed and experimental values (Figure 4d) allows use of these results for a deeper analysis.

Calculations of chemical shifts for M_3_N@C_80_(Ad) derivatives are complicated by the dynamics of the cluster, which rotates around the ^Ad^M–N bond. Thus, DFT calculations localize several low-energy conformers with different rotational angles (vide infra). The cluster dynamics does not pose a problem for **Dy** **_1_** **-II-** **^Ad^** **Dy**, because orientation of the χ tensor and position of the ^Ad^Dy ion with respect to the Ad moiety does not change during the cluster rotation. The values obtained for the lowest-energy conformer or averaged over several conformers are nearly identical and only slightly overestimate experimental shifts, which can be corrected by scaling computed shifts by a factor of 0.964 (Table S1 and Figures S10 and S11).

Situation is more complicated for the **Dy** **_1_** **-II-** **^Ad^** **Sc** form because rotation of the cluster around the ^Ad^Sc–N bond changes the position of the Dy ion and orientation of its χ tensor. Chemical shifts, calculated for the main conformer of [6,6]-^Ad^Sc DySc_2_N@C_80_(Ad), do not give a good agreement with the experimental spectrum (Table S2, Figure S13a), and only a moderate improvement was achieved by averaging over six conformers (Table S2, Figure S13b). Despite these caveats, chemical shifts of **Dy** **_2_** **-II-** **^Ad^** **Dy** and **Dy** **_3_** **-III** calculated for their lowest-energy conformers (Figure 5 and Figures S16 and S17) agree with experimental values quite well (Figure 4d). These calculations showed that pseudocontact shifts induced by ^Ad^Dy ions are not constant but rather increase with the number of Dy ions in the cluster (Table S4). The main factors causing this trend are pushing the ^Ad^Dy ion closer to the Ad moiety and a concomitant shortening of the Dy–N bond length and a slight increase of the anisotropy term in eq 1 when the size of the M_3_N cluster increases. Contributions of the ^cage^Dy ions to paramagnetic shifts tend to decrease in the same row, but their accurate modeling requires explicit consideration of the cluster dynamics.

**Figure 5:**
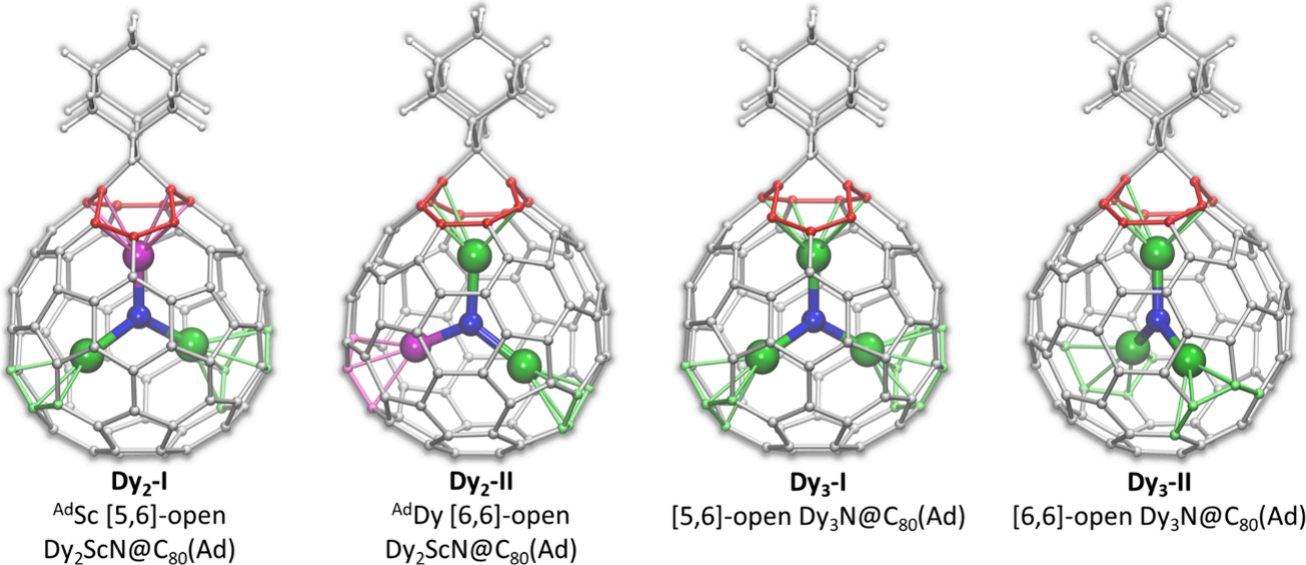
DFT-optimized molecular structures of the most stable conformers of **Dy_2_-I**, **Dy_2_-II**, **Dy_3_-I**, and **Dy_3_-II**. Dy – green, Sc – magenta, N – blue, C – gray, H – white. Two hexagons or pentagon and hexagon adjacent to the open bond are highlighted in red; Dy–C distances shorter than 2.55 Å and Sc–C distances shorter than 2.45 Å are shown as light-green and light-magenta bonds, respectively.

Chemical shifts of the ^Ad^Sc form of **Dy** **_2_** **-II** also defy simple additivity. Whereas **Dy** **_1_** **-II** exhibits eight well-defined ^Ad^Sc signals at 23–85 ppm, whose intensity is higher than that of the ^Ad^Dy form, for **Dy** **_2_** **-II**, we only detect four relatively weak ^1^H signals at 19–33 ppm. Thus, we can conclude that the equilibrium amount of the ^Ad^Sc form of **Dy** **_2_** **-II** is considerably smaller than that of ^Ad^Dy. Note that paramagnetic signals with shifts below 10 ppm are hard to detect on the background of diamagnetic impurities present in the sample, such as solvent residues, traces of the leaked HPLC column materials, and a lock.

For the isomers with the [5,6]-open addition site, much lower yield and requirements for prolonged acquisition time prevented a detailed NMR study. Nonetheless, measurements of **Dy** **_2_** **-I** demonstrated that it has several signals at 25–41 ppm, in the range of the ^Ad^Sc conformation, whereas peaks in the ^Ad^Dy range are at the noise level (Figure S6). Thus, ^Ad^Sc appears to be the dominant form of **Dy** **_2_** **-I**, which follows analogous observation for **Dy** **_1_** **-I**.^57^ Similar to the situation with the ^Ad^Sc form of **Dy** **_2_** **-II**, chemical shifts of **Dy** **_2_** **-I** are smaller than those in **Dy** **_1_** **-I**, indicating that the additivity rule does not hold here as well.

### DFT Calculations

Orientations of Dy_2_ScN and Dy_3_N clusters in Ad adducts were analyzed with DFT computations. Earlier studies of EMF-Ad derivatives demonstrated that metal atoms usually prefer coordination to the Ad addition site,^50–52,54–56^ whereas noncoordinating orientations of the M_3_N cluster are less stable by at least 45 kJ mol^–1^.^57^ Therefore, in this work we considered only the structures with metal-coordinated Ad site. A set of starting geometries was generated by rotating the endohedral cluster in ^Ad^Sc or ^Ad^Dy forms around the ^Ad^M–N bond with a step of 10–15°, and their optimization was then performed at the DFT level using PBE–PAW method in VASP.^64^ The procedure resulted in several unique conformers for each isomer, shown in Figures S18–S23. For Dy_2_ScN@C_80_(Ad), we found that the most stable [6,6]-open conformer (Figure 5) is lower in energy than the [5,6]-open counterpart by 16.8 kJ mol^–1^. Furthermore, the most stable ^Ad^Dy conformer of **Dy** **_2_** **-II** is lower in energy than the ^Ad^Sc form by 6.6 kJ mol^–1^, whereas the stability order in **Dy** **_2_** **-I** is reversed, with the ^Ad^Sc conformer being more stable than ^Ad^Dy but only by 0.3 kJ mol^–1^. Although these energy differences between the ^Ad^Dy and ^Ad^Sc forms are small and comparable to the thermal energy at room temperature, the switch of the DFT-favored structure from the ^Ad^Dy form for the [6,6] isomer to the ^Ad^Sc form for the [5,6] isomer agrees with the NMR results.

For Dy_3_N@C_80_(Ad), the difference between the [5,6] and [6,6] isomers is further increased to 21.6 kJ mol^–1^. The relative stability of the [6,6] isomer over [5,6] increases with the size of the endohedral cluster, which correlates with the experimental observation that the yield of the [5,6] isomer decreases dramatically on going from DySc_2_N@C_80_(Ad) to Dy_2_ScN@C_80_(Ad) to Dy_3_ScN@C_80_(Ad) (Table 1).

A notable effect of the addition of Ad to M_3_N@C_80_ is the change in the geometrical parameters of the endohedral cluster. The C_80_ cage has limited inner space, and encapsulation of the M_3_N cluster may induce a strain when metals are too large.^21,79^ Thus, the strain grows from DySc_2_N@C_80_ to Dy_2_ScN@C_80_ to Dy_3_N@C_80_, while Dy–N bonds shorten their length by ∼0.05 Å with each additional Dy atom. Opening of the σ C–C bond in M_3_N@C_80_(Ad) increases the space available to the metal-nitride cluster, which is reflected in the elongation of Dy–N bonds by 0.02–0.04 Å, from 2.106 Å in Dy_2_ScN@C_80_ to 2.135–2.152 Å in Dy_2_ScN@C_80_(Ad), and from 2.063 Å in Dy_3_N@C_80_ to 2.085–2.094 Å in Dy_3_N@C_80_(Ad).

The local geometry of the Dy-Ad coordination fragment is also visibly affected by the number of Dy ions in the M_3_N cluster. With the increase of the cluster size, the C–C distance in the open bond elongates from 2.138 Å in **Dy** **_1_** **-II** through 2.167 Å in **Dy** **_2_** **-II** to 2.204 Å in **Dy** **_3_** **-III** in the lowest-energy conformers thereof. The distance between Dy and the quaternary carbon of the Ad moiety decreases in the same series, from 3.418 Å in **Dy** **_1_** **-II** to 3.370 Å in **Dy** **_2_** **-II** to 3.322 Å in **Dy** **_3_** **-III**. These changes show that Dy is pushed closer to the Ad moiety in M_3_N@C_80_(Ad) with larger M_3_N clusters.

## Magnetic Properties of Dinuclear Systems: Dy_2_ScN@C_80_ and Dy_2_ScN@C_80_(Ad)

### Single-Ion Anisotropy

Ab initio CASSCF/RASSI-SO calculations (OpenMolcas code^80,81^ ) of DFT-optimized Dy_2_ScN@C_80_(Ad) molecules demonstrate that the ground-state Kramers doublet of each Dy ion corresponds to the pure state *m*_J_ = ± 15/2 with the pseudospin *g*_*z*_ of ∼19.8–19.9 and quantization axis aligned along the corresponding Dy–N bond with a deflection angle of no more than 2–3° (Table S7–S9). This is quite typical for Dy ions in nitride clusterfullerenes. Further nuances depend on how given Dy ion is positioned inside the fullerene.^82^ For metals coordinated to unfunctionalized fragments of the cage (^cage^Dy), the overall ligand-field (LF) splitting is in a range of 1430–1510 cm^–1^, whereas the gap between the first and the second KDs (KD1 and KD2) exceeds 365 cm^–1^, reaching 521 cm^–1^ in one of the coordination geometries. Note that CASSCF calculations likely underestimate the LF splitting by 15–20% because of the lack of dynamic correlation.^57,82,83^ When Dy is coordinated to the Ad-addition site, the LF splitting and KD1–KD2 gap are reduced to ∼1250 and ∼330 cm^–1^, respectively. The reduced magnetic axiality of the ^Ad^Dy ions is also apparent from the pseudospin *g*-tensor, as their *g*_*x*,*y*_ values of 0.003–0.004 are 1–2 orders of magnitude larger than for the ^cage^Dy ions.

### SMM Properties and Dy···Dy Coupling

Figure 6a,b and Figure S25 show low-temperature magnetization curves and FC/ZFC (field cooling/zero-field-cooled) measurements of **Dy** **_2_** **-I** and **Dy** **_2_** **-II**. Both compounds exhibit blocking of magnetization and opening of magnetic hysteresis. The blocking temperature *T*_B_, defined as the ZFC maximum, occurs at 5.0 K for **Dy** **_2_** **-I**, while its FC/ZFC bifurcation point is shifted to ∼6 K. Likewise, the hysteresis is open up to 5 K (already very narrow at this temperature) and closes by 6 K. For **Dy** **_2_** **-II**, the ZFC maximum occurs at 5.7 K, while the bifurcation point is observed at 10–11 K. Very narrow hysteresis opening of **Dy** **_2_** **-II** can be seen up to 10 K. These values can be compared to *T*_B_ = 8.0 K in the pristine Dy_2_ScN@C_80_.^20^ The shape of magnetic hysteresis of **Dy** **_2_** **-I** and **Dy** **_2_** **-II** without discernible drop of magnetization in zero field is common for Dy_2_ScN@C_80_ ^22^ and other dinuclear Dy-clusterfullerenes.^13,15,19,21^ Coercive fields at 1.8 K of 0.51 T for **Dy** **_2_** **-I** and of 0.58 T for **Dy** **_2_** **-II** are smaller than 0.70 T observed for Dy_2_ScN@C_80_. Thus, the blocking temperature and coercivity criteria show that the Ad addition softens the SMM performance of Dy_2_ScN@C_80_ irrespective of the regioisomery.

**Figure 6:**
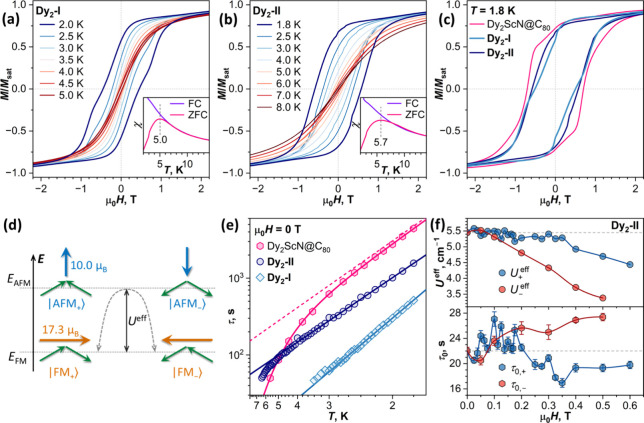
(a, b) Magnetic hysteresis curves of (a) **Dy_2_-I** and (b) **Dy_2_-II**; insets show comparison of field-cooled (FC) and zero-field-cooled (ZFC) susceptibility curves for determination of the blocking temperature of magnetization *T*_B_. (c) Comparison of magnetic hysteresis shapes for **Dy_2_-I**, **Dy_2_-II**, and Dy_2_ScN@C_80_ at 1.8 K. The average magnetic field sweep rate in hysteresis measurements was 2.9 mT s^–1^ (see Figure S25 for hysteresis curves measured at different sweep rates); FC/ZFC curves were measured at 0.2 T with a temperature sweep rate of 5 K min^–1^. (d) Schematic presentation of the lowest-energy magnetic states of the Dy_2_ScN cluster: orientation of Dy^3+^ magnetic moments (green arrows) in two quasi-doublets with FM and AFM alignment and total magnetic moments in these states (orange and blue arrows). (e) Zero-field magnetization relaxation times of **Dy_2_-I** and **Dy_2_-II** and their fitting with the Orbach mechanism τ^–1^ = τ_0_^–1^ exp­(−*U* ^eff^/*T*); only times longer than 100 s were used in the fits. Also shown are relaxation times of Dy_2_ScN@C_80_ and their fit with a combination of Orbach and Raman mechanisms from ref 24 (solid curve); contribution of Orbach mechanism is plotted as the dashed line. (f) *U* ^eff^ and τ_0_ values for **Dy_2_-II**, determined from temperature dependencies of τ_+_ and τ_–_ measured in different magnetic fields; to guide the eye, horizontal dashed lines mark *U* ^eff^ and τ_0_ determined in zero field. Error bars for τ_0_ values in panel (f) are uncertainties of Arrhenius fits; analogous uncertainties of *U* ^eff^ are smaller than the symbol size.

We recently found that coordination of Dy to the Ad-site in mononuclear DySc_2_N@C_80_(Ad) led to the lower blocking temperature of magnetization and narrower hysteresis in comparison to the unfunctionalized DySc_2_N@C_80_, whereas DySc_2_N@C_80_(Ad) with the Sc-coordinated Ad-site showed higher *T*_B_ than the latter.^57^ The single-molecule magnetism of DySc_2_N@C_80_ and its derivatives is inevitably determined by the single-ion magnetic properties, and the softer SMM indicators of the ^Ad^Dy form clearly correlate with its reduced magnetic axiality. For Dy_2_ScN@C_80_(Ad), NMR spectroscopy showed that **Dy** **_2_** **-I** mainly has ^Ad^Sc coordination with two ^cage^Dy ions, whereas **Dy** **_2_** **-II** is dominated by the form with one ^Ad^Dy and one ^cage^Dy ions. However, despite the lower axiality of its ^Ad^Dy ion, **Dy** **_2_** **-II** has slightly higher *T*_B_, considerably higher bifurcation temperature and similarly higher hysteresis closing temperature than **Dy** **_2_** **-I**. The differences in single-ion anisotropy are evidently of a lesser importance for Dy_2_ScN@C_80_(Ad) isomers, suggesting that their low-temperature magnetization dynamics is chiefly determined by Dy···Dy interactions. This conclusion agrees with the recent computational study of spin-relaxation mechanism in a di-Co exchange-coupled system.^84^

Alignment of Dy moments in the Dy_2_ScN nitride cluster along Dy–N bonds leads to two quasi-doublets with ferromagnetic (FM) and antiferromagnetic (AFM) alignment of Dy magnetic moments (Figure 6d). Since Dy moments in the Dy_2_ScN cluster are noncollinear and make an angle of α ≈ 120°, the total magnetic moment of the AFM state is not zero but close to 10 μ_B_, whereas that of the FM state is near 17.3 μ_B_. The FM quasi-doublet of Dy_2_ScN@C_80_ is more stable than the AFM quasi-doublet by 5.6 cm^–1^, which creates an exchange bias preventing the zero-field quantum tunneling of magnetization (QTM). The QTM dominates the relaxation of magnetization of single-ion systems, including DySc_2_N@C_80_ and DySc_2_N@C_80_(Ad), and leads to the characteristic shape of magnetic hysteresis with an abrupt drop in magnetization near zero field. In the Dy_2_ScN cluster, a flip of one Dy magnetic moment promotes the system from the FM to the AFM state, which requires additional energy and therefore makes the one-moment zero-field QTM impossible. The QTM, in which both Dy moments flip at once, is a low-probability process and was clearly observed in EMFs only at very low temperatures.^17,85^ The QTM in zero field is thereby suppressed, and magnetic hysteresis of both Dy_2_ScN@C_80_(Ad) isomers has significant remanence and coercivity similar to that of the nonfunctionalized Dy_2_ScN@C_80_. The shape of the magnetization curves suggests FM coupling in Dy_2_ScN@C_80_(Ad). The FM-coupled ground state is also evidenced for both Dy_2_ScN@C_80_(Ad) isomers by the sharp low-temperature peak in their χ*T* curves (Figure S26).

Magnetization relaxation times of **Dy** **_2_** **-I** and **Dy** **_2_** **-II** were studied by DC magnetometry (Figures S27–S31, Tables S12–S22). Zero-field times of the adducts appeared faster than in Dy_2_ScN@C_80_ for the most of the low-*T* range and showed linear dependences in Arrhenius coordinates (Figure 6e), giving the relaxation barriers of 9.7 ± 0.1 K (6.7 cm^–1^) for **Dy** **_2_** **-I** and 7.7 ± 0.1 K (5.4 cm^–1^) for **Dy** **_2_** **-II**. These barrier heights are comparable to 8.0 K (5.6 cm^–1^) in Dy_2_ScN@C_80_.^24^ The Orbach mechanism with *U* ^eff^ value of several K is often observed in the low-temperature magnetic relaxation of dinuclear EMF-SMMs, and its barrier corresponds to the energy difference between the FM and AFM states (Figure 6d).^12,13,15,17,19,21,22^ χ*T* curves simulated in the assumption that the energy difference between FM and AFM states is equal to *U* ^eff^ values reproduce experimental curves quite well (Figure S26). An alternative explanation for the Arrhenius behavior might be the Raman mechanism involving a low-frequency optical mode (also known as “local mode” mechanism).^86–89^ However, DFT-computed intramolecular vibrational frequencies of Dy_2_ScN@C_80_(Ad) are higher than 20 cm^–1^ (Table S6, Figure S24), suggesting that the local mode mechanism in this case is unlikely. Thus, based on the relaxation barriers, we can conclude that the Dy···Dy coupling in **Dy** **_2_** **-I** is 20% stronger, while in **Dy** **_2_** **-II**, it is of the same size as in the nonfunctionalized Dy_2_ScN@C_80_ (Table 2). However, Ad addition still leads to a faster relaxation below 5 K because of reduced attempt times, particularly in **Dy** **_2_** **-I** (τ_0_ = 2.4 ± 0.1 s), and to a lesser extent in **Dy** **_2_** **-II** (τ_0_ = 21.2 ± 0.5 s), in comparison to Dy_2_ScN@C_80_ (τ_0_ = 56 ± 1 s). Above 5 K, the relaxation of Dy_2_ScN@C_80_ outpaces that of **Dy** **_2_** **-II** because the former switches to the Raman mechanism with a faster acceleration of relaxation (Figure 6e). From relaxation measurements, we can also determine another SMM figure of merit and temperature at the relaxation time of 100 s, which amounts to *T*_B100_ = 2.6 K in **Dy** **_2_** **-I** and *T*_B100_ = 4.9 K in **Dy** **_2_** **-II** and Dy_2_ScN@C_80_.

**Table 2:** Dy···Dy Coupling Constants, Relative Energies and Magnetic Moments of Exchange-Coupled States of Dy_2_ScN@C_80_, Dy_3_N@C_80_, and their Ad Monoadducts

adduct	*j*_12_, *j*_13_, *j*_23_, cm^–1^	Δ*E*, cm^–1^	μ, μ_B_
Dy_2_ScN@C_80_	0.055	0.00, 5.60^a^	17.01, 10.54
**Dy**_**2**_ **-I**	0.057	0.00, 6.71^a^	17.48, 9.72
**Dy**_**2**_ **-II**	0.045	0.00, 5.38^a^	17.45, 9.65
Dy_3_N@C_80_	0.007, 0.007, 0.007	0.00, 0.02, 0.03, 1.59^b^	20.10, 19.97, 19.89, 0.16
**Dy**_**3**_ **-I**	0.055, 0.055, 0.017	0.00, 3.38, 3.94, 11.55^b^	19.35, 20.42, 19.97, 0.49
**Dy**_**3**_ **-II**	0.028, 0.042, −0.015	0.00, 4.31, 7.09, 8.21^b^	20.16, 20.60, 19.09, 0.93

a For dinuclear systems, Δ*E* is defined as the *U* ^eff^ value for the low-temperature Orbach mechanism, while *j* is determined to match the *U* ^eff^ value udenr Hamiltonian (1).

b For trinuclear systems, Δ*E* and *j* values are determined from matching experimental χ*T* and magnetization curves by simulations with Hamiltonian (1).

The relative positions of the FM and AFM states and the crossing of their levels determine the shape of magnetic hysteresis, which can be used for a complementary determination of the AFM-FM energy difference. For powder samples such as those studied in this work, molecular orientations with respect to the external field are distributed randomly. Two situations are then expected in the field dependence of magnetization, as depicted in Figure 7a. If the orientation of the external field is close to the orientation of the ground-state magnetic moment, FM states are more stable than AFM in the whole field range (see Zeeman diagram in the upper part of Figure 7a). If molecules are polarized in some high positive field to ensure the |FM_+_⟩ state and the field is then swept to the negative direction, no level crossing occurs down to zero field. In zero field, |FM_+_⟩ and |FM_–_⟩ levels cross, but the QTM is usually not very efficient at this kind of crossing in binuclear systems, as discussed above. As a result, many molecules will retain their magnetization until the next level crossing of the |FM_+_⟩↔|AFM_–_⟩ type (denoted by letter A in Figure 7a), at which the QTM is much more efficient. As the angular distribution of molecular orientations has a sharp maximum,^17,85^ the |FM_+_⟩↔|AFM_–_⟩ crossing produces an inflection in the hysteresis shape and a peak of the *dM*/*dH* derivative. From the field *H*_A_, at which this feature occurs, the FM-AFM energy difference can be estimated as Δ*E*_AFM–FM_ [cm^–1^] = 0.93*H*_A_[T]­μ_Dy_[μ_B_].^17^ Analysis of hysteresis shapes and measurements at different sweep rates (Figure S25) revealed the presence of the level-crossing features in **Dy** **_2_** **-I** at 0.75 T and in Dy_2_ScN@C_80_ at 0.52 T. From these values, Δ*E*_AFM–FM_ can be estimated as 7.0 and 4.9 cm^–1^, respectively, in reasonable agreement with the values obtained from the barrier heights discussed above. For **Dy** **_2_** **-II**, we could not identify the |FM_+_⟩↔|AFM_–_⟩ level-crossing features in the hysteresis shape. Presumably, the QTM at this crossing is less efficient than that in **Dy** **_2_** **-I.**

**Figure 7:**
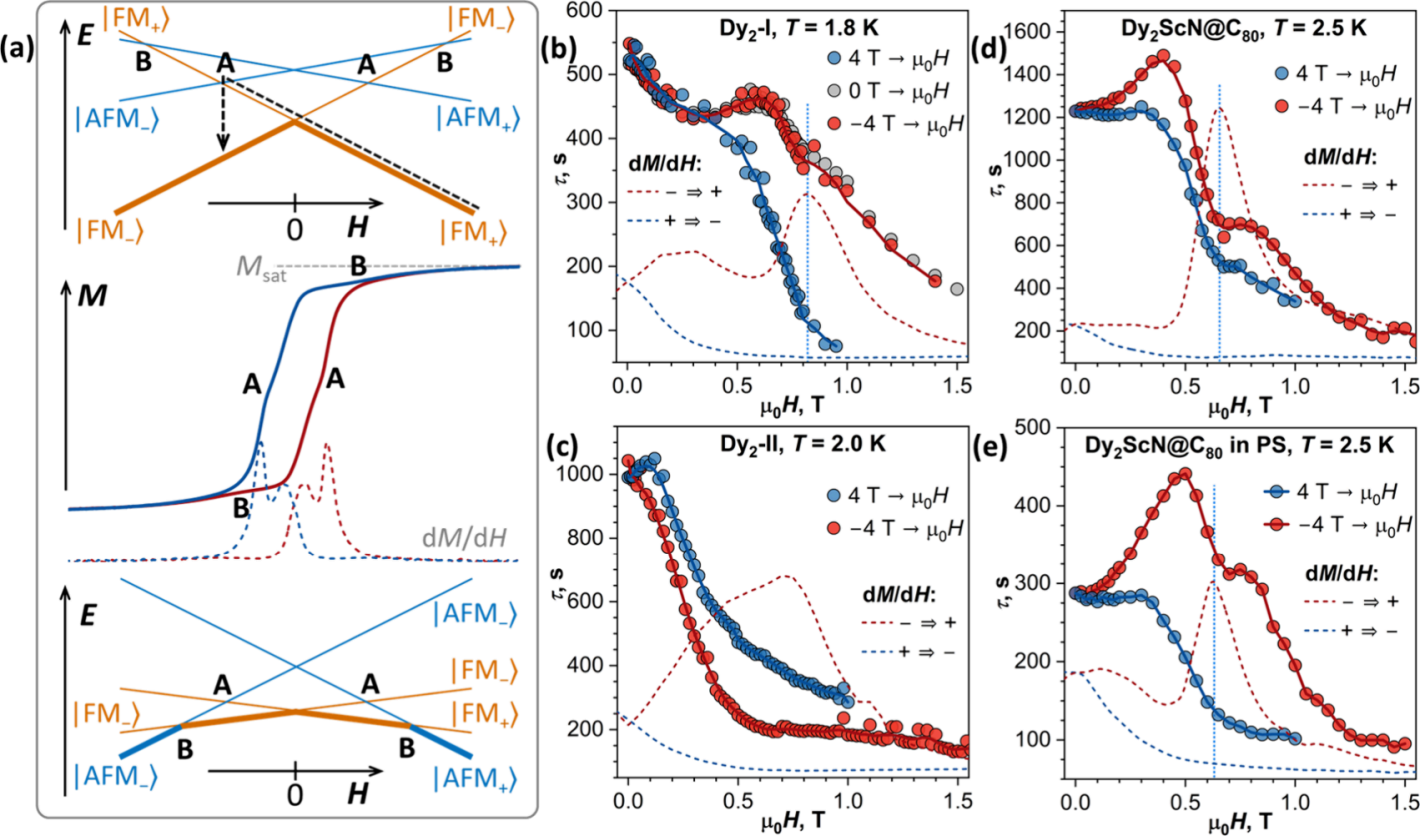
(a) Magnetic hysteresis of Dy_2_ScN@C_80_(Ad)-I with derivatives of magnetization (middle panel) and Zeeman diagrams for the Dy_2_ system with FM-coupled ground state and different orientations of the magnetic field (upper plot – field orientation is close to the magnetic moment of the FM state, bottom plot – field orientation is close to the magnetic moment of the AFM state); level crossings of type A and B correspond to features A and B in the magnetic hysteresis. (b–e) Field dependence of magnetization relaxation times of (b) **Dy_2_-I**, (c) **Dy_2_-II**, (d) Dy_2_ScN@C_80_, and (e) Dy_2_ScN@C_80_ diluted in polystyrene measured after magnetization of the sample at 4 T (blue, τ_+_) and −4 T (red, τ_–_). Also shown for each graph are derivatives of magnetic hysteresis curves at the same temperature (dashed curves).

For some molecules, the orientation of the external field will be closer to the magnetic moment of the excited AFM state. In this case, the AFM state will become more stable than the FM state at some field (bottom panel in Figure 7a). For such molecules, sweeping the field from the positive to negative direction will produce a crossing of |AFM_+_⟩ and |FM_+_⟩ levels (point B in Figure 7a, bottom), and corresponding inflection will occur in the hysteresis and equilibrium magnetization curves. But since the fraction of such molecules is small and the angular distribution is quite diffuse, the inflection in the hysteresis curve is smeared over the large field range. Nonetheless, its presence is responsible for the characteristic shape of the magnetization curve and for the much larger field required to saturate the magnetization in binuclear systems with noncollinear magnetic moments when compared to single-ion systems.

### Magnetic Field Dependence of Relaxation Times

The variation of the level energies and the presence of level crossings (see Zeeman diagrams in Figure 7a), as well as acceleration of the direct relaxation process with increasing field, suggest a significant magnetic field dependence of relaxation times. We studied the latter in two series of measurements. In the first series, the sample was magnetized at 4 T, then the field was ramped to a certain positive value μ_0_ *H* < 4 T, and the decay of magnetization was measured over time. In the second series, the sample was magnetized at −4 T, then the field was ramped to a positive μ_0_ *H*, and a gradual increase of magnetization was followed in this field. Both types of *M*–*t* dependencies were fitted with a stretched exponential function, giving relaxation times denoted hereafter as τ_+_ (for the 4 T → μ_0_ *H* route) and τ_–_ (for the −4 T → μ_0_ *H* route).

Figure 7b–e shows the results of such measurements for **Dy** **_2_** **-I**, **Dy** **_2_** **-II**, and Dy_2_ScN@C_80_. Near zero field, τ_+_ and τ_–_ values are similar, as follows from the time-reversal symmetry, but then diverge in higher fields. For **Dy** **_2_** **-I**, the divergence starts at 0.4 T, as τ_+_ decreases rapidly above this field, whereas τ_–_ first increases, reaches the maximum at 0.6 T, and only then starts a gradual decrease (Figure 7b). For **Dy** **_2_** **-II**, τ_+_ and τ_–_ diverge already in small fields, as τ_+_ shows a small increase and reaches the maximum near 0.1 T, whereas τ_–_ decays monotonously all the way starting from zero field (Figure 7c). Thus, differently from **Dy** **_2_** **-I**, the τ_+_ of **Dy** **_2_** **-II** is longer than τ_–_ in all studied fields. The field dependence of Dy_2_ScN@C_80_ is closer to that of **Dy** **_2_** **-I**: while τ_+_ remains constant between 0 and 0.3 T and then decreases fast, τ_–_ first grows from 0 to 0.4 T, then decreases abruptly between 0.4 and 0.65 T, stabilizes between 0.65 and 0.8 T, and then enters the second segment of decay at higher field.

The fact that the relaxation time measured in a certain field depends on the direction of the field ramp before the measurement may seem unusual but, in hindsight, should not be surprising. Consider Zeeman diagram in the upper part of Figure 7a. At large positive field, all molecules will be in the |FM_+_⟩ state. If the field is then ramped to a lower positive value, establishing a new equilibrium will require redistribution of the population from |FM_+_⟩ to |FM_–_⟩. Likewise, if the field is ramped from a large negative value, at which all molecules are in the |FM_–_⟩ state, then the relaxation of magnetization should be dominated by the transformation from |FM_–_⟩ to |FM_+_⟩. If the energies of |FM_+_⟩ and |FM_–_⟩ states are not equal (as they are for μ_0_ *H* ≠ 0) and relaxation proceeds via the same excited state (as in the Orbach mechanism), then the activation energies of the forward and backward processes will be obviously different. Of course, in the dynamic equilibrium, the rates of the forward and backward processes should be equal, but it does not mean that their time constants should be equal as well. Note that the difference between τ_+_ and τ_–_ cannot be observed by the AC technique, in which the field is oscillated near some constant value with a small amplitude of less than 1 mT, but can be straightforwardly measured using the DC technique. With the rapidly growing number of SMMs with long relaxation times, this effect should be encountered more and more often.

The dependence of relaxation time on the exposure of the sample to a certain field before the measurement is the essence of the hole-digging technique, developed for the studies of the QTM in SMMs.^90^ Different QTM relaxation rates are then caused by variation of dipolar magnetic fields, which are produced by intermolecular interactions. To evaluate the role of dipolar fields in our systems, we measured τ_+_ and τ_–_ for Dy_2_ScN@C_80_ diluted in polystyrene (Figure 7e, Figure S31, Tables S21 and S22). As distances between fullerene molecules in the diluted sample are increased, intermolecular dipolar magnetic interactions should be strongly diminished. Dilution unexpectedly resulted in a considerable shortening of the relaxation times, but the shapes of the τ_+_ and τ_–_ field dependencies remained similar (Figure 7d,e). Furthermore, the ratio of relaxation times of powder and PS-diluted sample is different for τ_+_ and τ_–_, and both change with the field (Figure S32). A similar effect was earlier observed for the dilution of DySc_2_N@C_80_ in PS, which slowed down the QTM relaxation near zero field, but accelerated relaxation at higher fields.^30^ Acceleration of relaxation in the diluted sample may be caused by the increased phonon density of states in the polymer matrix. Lowest-frequency intramolecular vibrations of Dy_2_ScN@C_80_ occur at 38 cm^–1^ ^24^ and thus not relevant for the relaxation of magnetization at 2.5 K, which therefore should be driven by acoustic phonons. Amorphous polymers are characterized by the excess of vibrational density of states at very low frequencies (dubbed as the boson peak),^91–93^ which can provide additional relaxation channels for the embedded molecular magnet. On the other hand, acceleration of relaxation with dilution is also a characteristic for the systems experiencing the phonon bottleneck.^94^ For them, dilution changes the balance between the spin–lattice and lattice-bath relaxation processes, providing more relaxation channels for the spin system.^95^ The bottlenecked Orbach mechanism is predicted to show the *H* ^0.5^ field dependence for relaxation times,^96^ but our field dependence is more complex and has several regimes (Figure 7, Figure S32), which precludes the definitive conclusion.

In another experiment aimed at creating a different local environment during relaxation of magnetization, we measured τ_–_′ by ramping the field to μ_0_ *H* from 0 T rather than from −4 T and found that this alteration did not affect τ_–_ relaxation times within the accuracy limits of the measurement (Figure 7b). Thus, a variation of concentrations of the |FM_+_⟩ and |FM_–_⟩ forms does not induce detectable differences in relaxation times, at least as long as the dominant form is not changed.

Divergence between τ_+_ and τ_–_ in the applied magnetic field can be either caused by variation of parameters for a given relaxation mechanism or by the influence of different relaxation mechanisms when the sample is subjected to alternative premeasurement magnetization routes. While zero-field relaxation of Dy_2_ScN@C_80_ and its Ad adducts is driven by the Orbach mechanism (Figure 6e), the magnetic field can also induce a direct mechanism and the QTM, the latter when the field is in the vicinity of the level crossing. The Zeeman diagram in Figure 6a demonstrates that the −4 T → μ_0_H route can include the level crossing, whereas the 4 T → μ_0_H route will not. The QTM manifests itself by the acceleration of relaxation near the (anti)­crossing point. A dip in the τ_–_-*H* dependence at 0.65 T coinciding with the peak of the d*M*/d*H* derivative and the absence of an analogous feature in the τ_+_-*H* dependence are indeed observed for Dy_2_ScN@C_80_. **Dy** **_2_** **-I** may also have a similar albeit strongly smeared τ_–_ feature near 0.8 T, whereas **Dy** **_2_** **-II** has none. The clearness of the QTM signature in the τ_–_-*H* dependence correlates with the shape of the magnetic hysteresis, which has a well-pronounced and sharp QTM feature for Dy_2_ScN@C_80_ (Figure 6c), somewhat smeared but still discernible feature for **Dy** **_2_** **-I** (Figure 6a) and none for **Dy** **_2_** **-II** (Figure 6b).

To reveal further details of the relaxation mechanism, we studied the temperature dependence of τ_–_ and τ_+_. Measurements were performed for **Dy** **_2_** **-II**, as it gives Arrhenius behavior in zero field in a broader temperature range than Dy_2_ScN@C_80_ and its relaxation times are sufficiently long to allow measurements at higher temperature than those for **Dy** **_2_** **-I** (Figure 6e). In the field up to 0.5–0.6 T, temperature dependence of both τ_–_ and τ_+_ showed Arrhenius behavior, suggesting that the direct mechanism is still not very efficient, while the Orbach mechanism dominates. At higher fields, relaxation becomes considerably faster (Figure 7), as the direct mechanism presumably starts to take over. In fact, the competition between the Orbach and direct mechanisms should have a more complicated form since the Zeeman splitting of the ground state pseudodoublet in Dy_2_ScN@C_80_(Ad) approaches the size of the Orbach barrier already when the field along *z* direction reaches 0.34 T. Unfortunately, trustworthy measurements of temperature dependence in above 0.5–0.6 T become impossible since reliability of DC technique for times shorter than 100 s is questionable.

Effective barriers and attempt times determined from temperature dependencies are plotted in Figure 6f. The *U*_+_^eff^ values (that is, determined from τ_+_ dependencies) remain near 5.2–5.5 cm^–1^ up to a field of 0.35 T and then decrease to 4.4 cm^–1^ at 0.6 T. Corresponding attempt times (τ_0,+_) increase on average but oscillate up to 0.2 T and then shorten at higher field. Oscillations of τ_0,+_ and *U*_+_^eff^ may be caused by hyperfine splitting as observed in a holmium metallacrown complex^97^ but can also reflect the level of experimental uncertainty. We tested the second option by implementing uncertainties of τ based on the β values from stretched exponential fitting,^98,99^ but such fits did not produce noticeable changes in determined τ_0,+_ and *U*_+_^eff^ values and their uncertainties (Table S18). The decrease of both τ_0,+_ and *U*_+_^eff^ above 0.35 T correlates with the decrease of τ_+_ in isothermal measurements (Figure 7c), while the increase of τ_0,+_ with nearly constant *U*_+_^eff^ at lower field corresponds to the growth of τ_+_ below 0.1 T. The *U*_–_^eff^ values decrease with the field more monotonously and much faster than *U*_+_^eff^, reaching 3.4 cm^–1^ by 0.5 T. At the same time, τ_0,–_ values show a gradual increase. As isothermal measurements demonstrate a constant decrease of τ_–_ in the field (Figure 7c), the decrease of *U*_–_^eff^ outweighs the growth of τ_0,–_.

The reasons for these trends are not fully understood at this moment. In the measurements of τ_–_, the dominant relaxation route under the Orbach mechanism should be |FM_–_⟩ → |AFM_±_ ⟩ → |FM_+_⟩. The energy difference between |FM_–_⟩ and |AFM_±_ ⟩ states decreases in the positive field, and hence, a decrease of *U*_–_^eff^ might be expected as indeed observed. Recent computational studies of spin-phonon relaxation in molecular magnets demonstrated that optical modes play a paramount role, whereas conclusions derived from the Debye model of phonons are not very relevant for SMMs.^87,100^ Yet, as the low-temperature barriers for Dy_2_ScN@C_80_ and Dy_2_ScN@C_80_(Ad) are considerably smaller than the frequencies of intramolecular vibrations (Table S6), we suggest that the spin relaxation here is mainly driven by lattice phonons. Then, the Debye model still should be valid, and the attempt time of the Orbach process should scale as the third power of *U* ^eff^.^101^ If so, a decrease of the *U*_–_^eff^ should also correspond to the decrease of τ_0,–_, but this is opposite the experimental results. Following the same logic, the dominant relaxation route for τ_+_ should be |FM_+_⟩ → |AFM_±_ ⟩ → |FM_–_⟩, leading to the growth of *U*_+_^eff^ and τ_0,+_ with the field. Instead, we observe constant *U*_+_^eff^ in a part of the field range followed then by its decrease, while τ_0,+_ similarly defies the expectations. One of the factors complicating the interpretation may be a distribution of molecular orientations, which smears the field dependencies. Another factor is that the energies of the barriers are quite low and comparable to thermal energy, whereas asymptotic formulas derived for the Orbach relaxation mechanisms imply the energy of the excited state being considerably higher.^101^ Mixing of lattice and low-frequency molecular modes^24,102^ may lead to deviations from the Debye model, while low energies of the barriers make the system susceptible to the phonon bottleneck.^96^ Thus, we believe that a more elaborate microscopic approach to spin-phonon relaxation will be required to fully understand these results and hope that our work will motivate in-depth theoretical studies of these phenomena.

## Magnetic Properties of Trinuclear Systems: Dy_3_N@C_80_ and Dy_3_N@C_80_(Ad)

### Single-Ion Anisotropy

The lowest-energy conformer of isolated Dy_3_N@C_80_ molecule has *C*_3_ symmetry with equivalent Dy ions, each coordinating cage hexagon and showing quasi-η^6^ hapticity. Our CASSCF calculations predict an LF splitting of 1377 cm^–1^ and the ground state doublet with *J* *_*z*_* = ±15/2. Addition of an Ad group affects the single-ion anisotropy of Dy ions in a similar way as already discussed for Dy_2_ScN@C_80_(Ad). The ^Ad^Dy atom in Dy_3_N@C_80_(Ad) has somewhat reduced LF splitting (1252–1370 cm^–1^) and increased *g* *_x,y_* values, whereas ^cage^Dy atoms retain higher axiality and even increase LF splitting to 1470–1560 cm^–1^ (Tables S10 and S11). For both types, the ground-state Kramers doublet is separated from the second KD by more than 300 cm^–1^ and has *J* *_*z*_* = ± 15/2 with the quantization axis aligned parallel with the corresponding Dy–N bond.

### Magnetic Frustration in Dy_3_N@C_80_

Triangular arrangement of magnetic moments is an archetypical model for several peculiar phenomena, such as toroidicity^103–108^ and magnetic frustration.^22,28,109–112^ Frustration in triangular lattices is usually associated with antiferromagnetic coupling, but in Dy_3_N@C_80_, frustration appears for ferromagnetically coupled moments.^22,28^ Since Dy magnetic moments are pinned to the Dy–N bonds, they are located in the plane of the triangle and arranged at an angle of 120° to each other. This arrangement prevents simultaneous ferromagnetic alignment of the moments for all three Dy···Dy contacts. When two pairs are aligned ferromagnetically, the third one inevitably adopts an antiferromagnetic alignment (Figure 8a). The ground state of this system is 6-fold degenerate. At the same time, simultaneous antiferromagnetic alignment in all three Dy···Dy pairs meets no geometric limitations, yielding a nonmagnetic doublet at higher energy.

**Figure 8:**
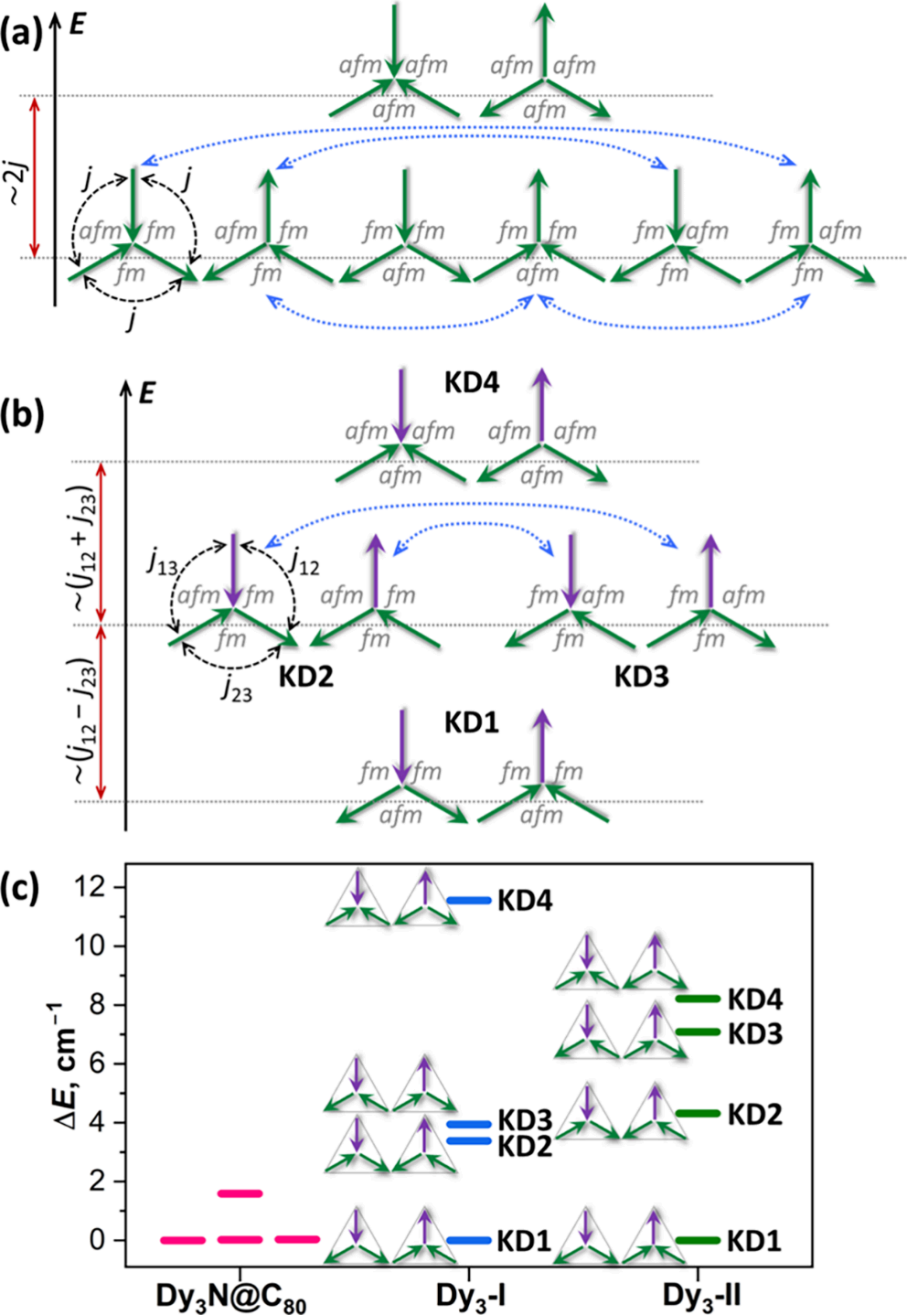
Schematic alignment of magnetic moments in the Dy_3_N cluster in (a) Dy_3_N@C_80_ and (b) Dy_3_N@C_80_(Ad), the latter in the assumption of a magnetic equivalence of two Dy ions and *j*_12_ = *j*_13_ > *j*_23_. Magnetic moments of individual Dy^3+^ ions are shown as green arrows (coordination to the unfunctionalized part of the cage) or dark violet arrows (^Ad^Dy coordination). Blue dotted arrows denote (some of) the possible QTM pathways between different states involving a flip of one Dy^3+^ magnetic moment. (c) Energies of the most stable Kramers doublets (KDs) of Dy_3_N@C_80_, **Dy_3_-I**, and **Dy_3_-II** deduced from simulations of χ*T* and magnetization curves.

The frustrated ground state of Dy_3_N@C_80_ eliminates the exchange bias, which prevents zero-field QTM in Dy_2_ScN@C_80_. A flip of one Dy moment in the sextet ground state of Dy_3_N@C_80_ produces another component of the sextet, and all six states can be interconverted in zero field via single-moment QTM steps. Thus, although Dy_3_N@C_80_ exhibits magnetic hysteresis below 5 K, the opening is very narrow, especially in the field smaller than 0.1 T, where the fast relaxation of magnetization is driven by the QTM (Figure 9a, Figure S33a). Zero-field QTM in Kramers systems is strongly dependent on intermolecular dipolar magnetic fields, which facilitate the opening of the tunneling gap. Indeed, when we diluted Dy_3_N@C_80_ in polystyrene to reduce local dipolar fields, the hysteresis opening in zero field became visibly broader (Figure 9a, Figure S33b,c).

**Figure 9:**
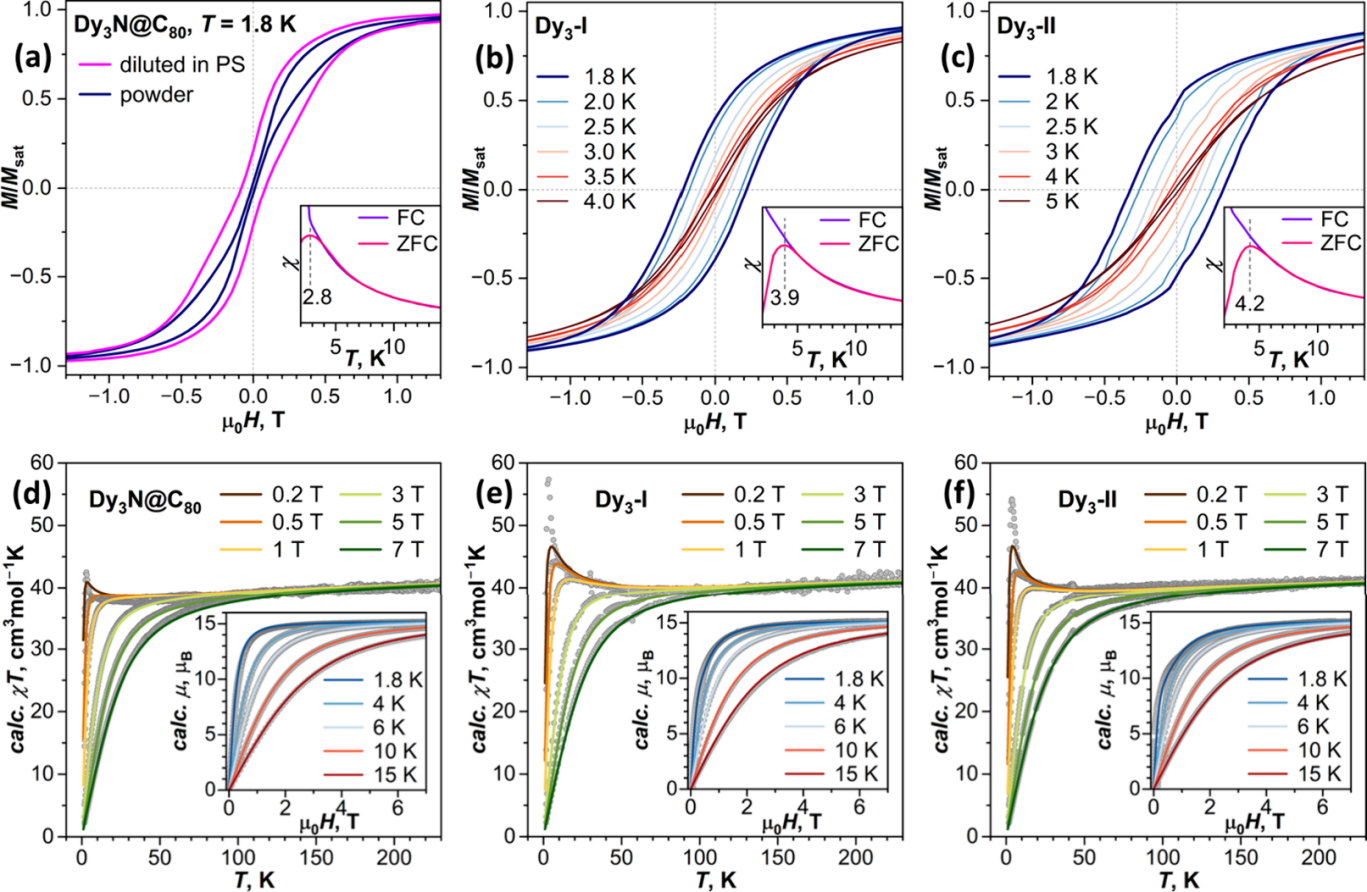
(a) Magnetic hysteresis of Dy_3_N@C_80_ powder and Dy_3_N@C_80_ diluted in polystyrene (PS) measured at 1.8 K. (b, c) Magnetic hysteresis curves of (b) Dy_3_–I and (c) Dy_3_–II measured at different temperatures. Insets in panels (a–c) show a comparison of field-cooled (FC) and zero-field-cooled (ZFC) susceptibility curves for determination of the blocking temperature of magnetization *T*_B_. Average magnetic field sweep rate in hysteresis measurements was 2.9 mT s^–1^; FC/ZFC curves were measured at 0.2 T with a temperature sweep rate of 5 K min^–1^. (d–f) Comparison of experimental (dots) and simulated (solid lines) χ*T* and magnetization curves for: (d) Dy_3_N@C_80_, (e) Dy_3_–I, and (f) Dy_3_–II; χ is defined as *M*/*H.*

The structure of the energy levels of Dy_3_N@C_80_ can be mapped onto a simple effective spin Hamiltonian:

$${Ĥ}_{\mathrm{spin}}=\underset{i}{\sum }{Ĥ}_{{\mathrm{LF}}_{i}}-2j_{12}{Ĵ}_{1}\cdot {Ĵ}_{2}-2j_{13}{Ĵ}_{1}\cdot {Ĵ}_{3}-2j_{23}{Ĵ}_{2}\cdot {Ĵ}_{3}+{Ĥ}_{\mathrm{ZEE}}$$ 2

where *Ĥ*_LF*_i_*_ are single-ion ligand-field Hamiltonians of Dy^3+^ ions, *i* runs from 1 to 3, Dy···Dy coupling is modeled by a bilinear product of total angular momentum operators of Dy ions ( ${Ĵ}_{1}$ , ${Ĵ}_{2}$ , and ${Ĵ}_{3}$ ) scaled by effective coupling constants *j*_*ij*_, and *Ĥ*_ZEE_ is the Zeeman term describing interaction with magnetic field. Operators ${Ĵ}_{i}$ operate on the space of the ^6^H_15/2_ multiplet of Dy^3+^ and are defined in their individual frames, the quantization axis for each Dy being roughly parallel to the corresponding Dy–N bond. Essentially, the ligand-field terms in Hamiltonian (2) split the ground-state ^6^H_15/2_ multiplet of each Dy ion into 8 KDs, whereas interaction terms couple individual states of different ions. The overall Hamiltonian space spans 4096 states, of which only 8 lowest-energy ones, forming four Kramers doublets (Figure 8), will be discussed hereafter. When employing Hamiltonian (2) to model magnetic properties of Dy_3_N@C_80_ and Dy_3_N@C_80_(Ad) as discussed below, we used PHI code^113^ with powder-averaging, while ligand-field parameters and angles between quantization axes were transferred from CASSCF calculations.

When three Dy ions are equivalent by symmetry and all Dy···Dy coupling constants are equal and positive, Hamiltonian (2) gives the spectrum of the lowest-energy levels shown in Figure 8a. In ref 22, the gap between the sextet and the doublet of 2.4 ± 1.6 cm^–1^ was estimated by fitting the low-temperature magnetization curve. The comparably large uncertainty interval was caused by the weak dependence of the magnetization on the energy of the nonmagnetic AFM state (Figure S34). The χ*T* product is more sensitive to this energy, and our simulations using Hamiltonian (2) with different coupling constants demonstrate that the gap of 1.6 ± 0.4 cm^–1^ (corresponding to *j* = 0.007 cm^–1^) gives the closest agreement to experimental χ*T* and magnetization data (Figure 9d, see also Figure S35).

### SMM Properties and Dy···Dy Coupling in Dy_3_N@C_80_(Ad)

Magnetization measurements of Dy_3_N@C_80_(Ad) isomers revealed a considerable effect of Ad addition. Both compounds show an open hysteresis without visible signatures of the QTM (Figure 9b,c, Figure S36) and with the coercivity of 0.22 T in **Dy** **_3_** **-I** and 0.32 T in **Dy** **_3_** **-II** when measured at 1.8 K with a sweep rate of 2.9 mT s^–1^. Opening is observed up to 4 K in **Dy** **_3_** **-I** and up to 5 K in **Dy** **_3_** **-II**. Their blocking temperatures near 4–5 K are lower than in mono and di-Dy congeners but higher than in Dy_3_N@C_80_. Relaxation times measured by the DC technique in zero field exhibit Arrhenius behavior with attempt times of 3.0(2) and 5.5(2) s and effective barriers of 5.7(1) and 5.5(2) cm^–1^ for **Dy** **_3_** **-I** and **Dy** **_3_** **-II**, respectively (Figure S37). As we demonstrate below, these values fall into the energy range of the exchange-coupled states of the Dy_3_N cluster in Dy_3_N@C_80_(Ad). However, the reliability of these barriers is limited by a small temperature interval in which these temperature dependencies can be measured before relaxation times become too short. On the scale of the best single-ion^114–119^ and dinuclear^38,120–126^ lanthanide SMMs, the SMM performance of Dy_3_N@C_80_(Ad) is quite modest, but among more than 50 trimetallic Dy SMMs described after the first report in 2006,^103^ only a small fraction showed an open hysteresis at 1.8 K,^22,112,127–132^ and only one^111^ has its hysteresis closing temperature higher than in Dy_3_N@C_80_(Ad).

Suppression of the zero-field QTM indicates that magnetic frustration in Dy_3_N@C_80_(Ad) derivatives is removed. Addition of Ad reduces molecular symmetry and makes Dy ions inequivalent, which lifts the 6-fold degeneracy. Since all Dy ions in Dy_3_N@C_80_(Ad) share an identical ground state with *J* *_*z*_* = ± 15/2, which is well separated from the first excited LF state, modification of the single-ion anisotropy cannot lift the degeneracy at helium temperatures. Therefore, we can conclude that the main factor responsible for the breakdown of frustration in Dy_3_N@C_80_(Ad) is the asymmetry of the Dy···Dy interactions. In fact, Ad addition also affects Dy–N–Dy angles in the Dy_3_N cluster, and the angles between quantization axes of Dy ions are not strictly 120°, but rather α_12_ = 117.8°, α_13_ = 120.4°, and α_23_ = 121.2° in **Dy** **_3_** **-I** and α_12_ = 117.4°, α_13_ = 125.3°, and α_23_ = 116.8° in **Dy** **_3_** **-II** (the values listed are for the lowest-energy conformers). This effect alone would split the sextet even if all coupling constants in Hamiltonian (2) remain equal, but the splitting introduced this way is on the order of 1 cm^–1^ and is not large enough to suppress the QTM in powder samples.

Although the structure of Dy_3_N@C_80_(Ad) has no rigorous 2-fold symmetry, ^cage^Dy ions should have a similar structural environment and properties, as already pointed out above based on CASSCF calculations. Therefore, as a starting approximation, we assume that *j*_12_ = *j*_13_ ≠ *j*_23_ (^Ad^Dy has number 1; two ^cage^Dy numbers numbered 2 and 3). Under Hamiltonian (2), this assumption splits the sextet into a doublet and a quartet (Figure 8b). If the quartet were the ground state (*j*_12_ = *j*_13_ < *j*_23_), some single-ion QTM transitions would be still possible in zero field, which is not observed experimentally. We therefore propose that the *j*_12_ = *j*_13_ > *j*_23_ relation is more plausible as it leads to the doublet ground state depicted in Figure 8b, for which QTM in zero field will be suppressed by the exchange bias. Starting from this conjecture, we simulated magnetization and χ*T* curves of both Dy_3_N@C_80_(Ad) isomers employing Hamiltonian (2), in which we varied Dy···Dy coupling constants (Figures S38–S43). These simulations revealed that the magnetization curve measured at 1.8 K is almost insensitive to the energy of the AFM state (KD4), but it depends considerably on the splitting of KD1-KD2-KD3, whereas the shape of the χ*T* curve shows weak dependence on the splitting of the first three KDs but is more sensitive to the KD4 energy. Thus, combining χ*T* and magnetization data is crucial to achieve a reasonable description of the exchange-coupled states in Dy_3_N@C_80_(Ad). For **Dy** **_3_** **-I**, the original conjecture of equal *j*_12_ and *j*_13_ constants appeared sufficient, while for **Dy** **_3_** **-II**, we further split the *j*_12_ and *j*_13_ values to achieve better agreement with experimental data.

Energies of four exchanged KDs obtained as a result of these simulations are compared in Figure 8c and Table 2, whereas corresponding χ*T* and magnetization curves are plotted in Figure 9e,f. Intermediate steps of simulations are outlined in the Supporting Information. For **Dy** **_3_** **-I**, we obtained coupling constants of 0.055, 0.055, and 0.017 cm^–1^, with KD energies of 0.0, 3.4, 3.9, and 11.6 cm^–1^. For **Dy** **_3_** **-II**, coupling constants are 0.028, 0.042, and −0.015 cm^–1^, while KD energies are 0.0, 4.3, 7.1, and 8.2 cm^–1^. In the view of the simple form of the effective spin Hamiltonian (2), coupling constants should be understood as merely parameters setting the relative energies of exchange KDs (Figure S38) rather than reflecting a certain coupling mechanism. These values demonstrate that the exohedral addition of adamantylidene has a profound effect on intracluster magnetic interactions in Dy_3_N@C_80_ and thereby boosts the SMM performance of Dy_3_N@C_80_(Ad) in comparison to the pristine fullerene.

## Conclusions

In this work, we studied the influence of adamantylidene addition on single-molecule magnetism of nitride clusterfullerenes with dinuclear and trinuclear Dy clusters Dy_2_ScN@C_80_ and Dy_3_N@C_80_. The original SMM properties of these metallofullerenes are strongly modulated by Dy···Dy interactions, which suppress the QTM by the exchange bias in Dy_2_ScN@C_80_, but lead to frustration with pronounced QTM in Dy_3_N@C_80_. We found that in the photochemical reaction with adamantane aziridine, each of these fullerenes forms two isomers of Ad monoadducts, whose relative yield depends on the size and shape of the nitride cluster. In particular, the yield of the [6,6] isomer increases dramatically in the DySc_2_N–Dy_2_ScN–Dy_3_N series. Since coordination of the Ad-addition site by Dy results in large ^1^H paramagnetic shifts of several hundred ppm, paramagnetic NMR was quite instrumental to determine the preferable coordination mode of the Dy_2_ScN cluster in Dy_2_ScN@C_80_(Ad). The Sc-Ad coordination is found to be prevalent for the [5,6] isomer, whereas the Dy-Ad coordination is more preferred for the [6,6] isomer.

In Dy_2_ScN@C_80_(Ad), Ad addition has almost no effect on the strength of the Dy···Dy coupling in the [6,6] isomer but does increase the coupling in the [5,6] counterpart by 20%. The blocking temperature of magnetization and the coercivity are both softened by adamantylidene addition irrespective of the isomeric structure of Dy_2_ScN@C_80_(Ad). At the same time, the differences in single-ion anisotropy appear to be of lower importance for the SMM properties of Dy_2_ScN@C_80_(Ad) isomers, suggesting that their low-temperature magnetization dynamics are mainly determined by Dy···Dy interactions. For the trinuclear system, we found that the addition of adamantylidene substantially increases Dy···Dy coupling constants and the energy spread of exchange-coupled states of the Dy_3_N cluster. It also imposes asymmetry onto Dy···Dy interactions in the triangular Dy_3_N cluster, thereby lifting the geometric frustration and degeneracy of the ground-state sextet. As a result, both Dy_3_N@C_80_(Ad) isomers exhibit open hysteresis without pronounced QTM signatures and have higher blocking temperature of magnetization than the pristine Dy_3_N@C_80_. Thus, chemical derivatization of the fullerene cage can influence the metal–metal coupling in endohedral species, and the magnitude of the effect can vary from insignificant to quite substantial. Either way, derivatization has a visible influence on the relaxation of magnetization and SMM properties of dinuclear and trinuclear EMF-SMMs, which can be utilized for the fine-tuning of the SMM properties in multinuclear EMFs and should be considered when designing functional derivatives thereof.

## Supplementary Material



## Notes

Experimental and computational details, synthesis and HPLC separation, spectroscopic characterization, computational results, and results of magnetic measurements (PDF)
