# Supplementary material for: Carbene Addition and Its Remote Influence on Dy···Dy Coupling, Relaxation of Magnetization, and Magnetic Frustration in Fullerene Single-Molecule Magnets
Source: JACS Au. 2025 Nov 25;5(12):6134–50. doi: 10.1021/jacsau.5c01106 (PMC12728640; doi:10.1021/jacsau.5c01106)
Supplement: Supplementary file 1 [file au5c01106_si_001.pdf]

Carbene addition and its remote influence on Dy···Dy coupling, relaxation of magnetization, and magnetic frustration in fullerene single-molecule magnets

Matheus Felipe de Souza Barbosa,[$a] Wei Yang,[$a] Noel Israel,[a] Fupin Liu,[b] Bernd Büchner,[a] Stanislav M. Avdoshenko,[a]* Alexey A. Popov[a]*

[a] *Leibniz Institute for Solid State and Materials Research (IFW Dresden), Helmholtzstr. 20, 01069, Dresden, Germany*

[b] *Jiangsu Key Laboratory of New Power Batteries, Jiangsu Collaborative Innovation Center of Biomedical Functional Materials, School of Chemistry and Materials Science, Nanjing Normal University, Nanjing, 210023 China*

Supporting information

Experimental and computational details	S2
Synthesis and separation of $M_3N@C_{80}$(Ad)	S3
^{1}H NMR spectroscopy	S8
Calculations of paramagnetic chemical shifts	S10
DFT computations	S22
Low-frequency vibrations	S25
CASSCF calculations	S26
DFT-optimized Cartesian coordinates	S31
Magnetic properties of $Dy_2ScN@C_{80}$ and $Dy_2ScN@C_{80}$(Ad)	S36
Magnetic properties of $Dy_3N@C_{80}$ and $Dy_3N@C_{80}$(Ad)	S47
References	S54

Experimental and computational details

Reagents: Fullerenes $Dy_2ScN@C_{80}$ and $Dy_3N@C_{80}$ were obtained by us earlier[1, 2] using arc-discharge evaporation of graphite rods filled with graphite powder mixed with Dy_2O_3/Sc_2O_3 or pure Dy_2O_3 in low-pressure He atmosphere and using guanidine isocyanate as a source of nitrogen. 2-adamantane-2,3'-[3H]-diazirine was synthesized from 2-adamantanone following literature methodics.[3, 4]

HPLC: HPLC analysis and separation were performed for toluene solutions of fullerene and with toluene as an eluent, employing semipreparative COSMOSIL Buckyprep chromatographic columns (Nacalai Tesque) and Agilent 1260 Infinity II LC System. Recycling HPLC separation was performed using Sunflow 100 system (SunChrome).

Mass spectrometry: Matrix-assisted laser desorption/ionization time-of-flight (MALDI-TOF) mass-spectra were measured with a Bruker autoflex mass-spectrometer; TPBD (1,1,4,4 tetraphenyl-1,3-butadiene) was used as a matrix.

UV-Vis spectrometry: UV-vis-NIR absorption spectra were measured in CS_2 solution at room temperature with Shimadzu 3100 spectrophotometer.

NMR spectroscopy: ^1H NMR spectra were measured with 500 MHz Avance II spectrometer (Bruker) in CS_2 solution. For spectra measured in extended range (several hundred ppm), different phase correction had to be used in different parts of the spectra.

Magnetic measurements. Magnetic measurements of powder samples were performed using a Quantum Design VSM MPMS3 magnetometer. For powder measurements, samples were drop-casted from CS_2 solution. For dilution in polystyrene, several granules of solid PS were dissolved in 0.5 mL of toluene together with ~0.1 mg fullerene. Solution was then drop-cast on a glass slide heated on a plate to 120°C. The polymer film with fullerene left after evaporation of the solvent was transferred to a propylene SQUID capsule using wooden stick.

Average magnetic field sweep rate in hysteresis measurements was 2.9 mT s^{-1}. Relaxation times were determined from stretched exponential fits of magnetization decays, measured after the sample was magnetized in a high field, and then the field was ramped as fast as possible (70 mT s^{-1}) to the required field. Decay curves were then fitted with the function:

$$M(t) = M_{eq} + (M_0 - M_{eq}) \exp\left[-\left(\frac{t}{\tau}\right)^{\beta}\right]$$

Where M_{eq} and M_0 are the equilibrium and initial magnetizations, respectively, τ is the relaxation time and β is a parameter, characterizing distribution of relaxation rates in the sample. For a single-exponential decay, β = 1. Reliable estimation of τ requires the duration of the decay curve measurement to be at least 3 τ. For relaxation times shorter than ~100 s, the method becomes increasingly unreliable because substantial part of the sample relaxes during the field sweep, and therefore only a tail of distribution with longer times is measured.

Simulations of magnetic properties were performed with powder-averaging using PHI code.[5]

DFT computations. DFT optimization of $YSc_2N@C_{80}$(Ad) and $Y_3N@C_{80}$(Ad) conformers was first performed at the PBE level with implemented TZ2P-quality basis set for C, N, and H, and ECP basis set for Y using Priroda code.[6, 7] Conformers were then re-optimized at the PBE-D/PAW level using the VASP code and recommended pseudopotentials with f-shell in-core treatment for Dy;[8-12] to avoid artificial intermolecular interactions when using periodic VASP code, the molecules were centered inside a box with the size of 20 Å.

CASSCF calculations. *Ab initio* energies and wave functions of Dy^{3+} multiplets in $Dy_2ScN@C_{80}$(Ad) and $Dy_3N@C_{80}$(Ad) molecules have been calculated for DFT-optimized structures at the CASSCF/RASSI-SO level of theory using the quantum chemistry package OpenMOLCAS[13] and SINGLE_ANISO module.[14] Only one Dy atoms was treated *ab initio* at a time, while others were replaced with Y. The basis sets were ANO-RCC-VTZP for Dy and ANO-RCC-VDZP for other elements.

Synthesis and separation of $M_3N@C_{80}(Ad)$

In a typical procedure, 1.2−1.5 mg of endohedral metallofullerene (~1×10^{-3} mmol) and 20 equiv. of AdN_2 (2×10^{-2} mmol, 3.24 mg) were dissolved in 30 mL of anhydrous toluene and placed in a Schlenk tube. The mixture was degassed by freeze-pump-thaw cycles under reduced pressure for removing O_2 and then irradiated with a 365 nm LED light (LightningCure LC-L1 from Hamamatsu) at room temperature under N_2 atmosphere, with continuous stirring throughout the reaction. During the reaction, we periodically withdraw 0.5 mL aliquots of the solution using a syringe for chromatographic analysis (Buckyprep column, Φ = 10 mm × 250 mm, elution with toluene) at specified time intervals. To prevent significant build-up of bis-adducts, irradiation was stopped when conversion of the pristine fullerene was 50−75%. Two isomers of Ad monoadduct were then obtained by separation with linear and recycling HPLC, and their composition was identified by MALDI mass-spectrometry.

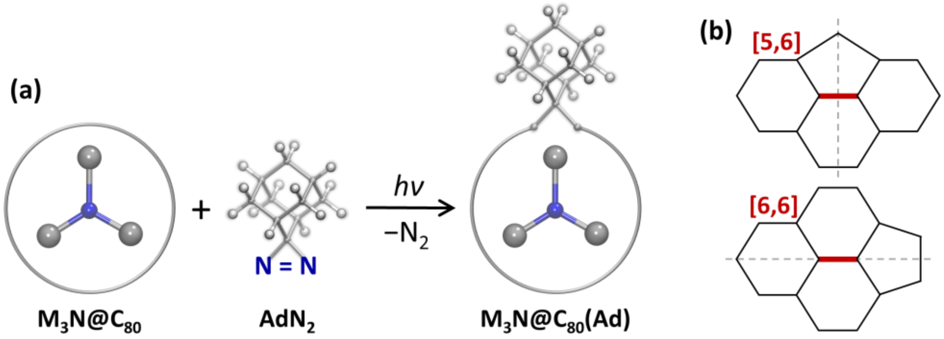

Figure S1. (a) Reaction scheme for photochemical addition of adamantylidene to $M_3N@C_{80}$; (b) two types of C−C bonds in I_h-C_{80} cage, giving two isomers of $M_3N@I_h$-$C_{80}(Ad)$.

Conversion of $M_3N@C_{80}$ and yield of $M_3N@C_{80}(Ad)$ isomers based on HPLC peak areas

$Dy_2ScN@C_{80}(Ad)$: conversion of $Dy_2ScN@C_{80}$ 72%; net yield of both $Dy_2ScN@C_{80}(Ad)$ isomers 64% (89% if counted on reacted $Dy_2ScN@C_{80}$), yield of $Dy_2ScN@C_{80}(Ad)$ isomer based on reacted $Dy_2ScN@C_{80}$: ~3% for Dy_2-I, 86% for Dy_2-II.

$Dy_3N@C_{80}(Ad)$: conversion of $Dy_3N@C_{80}$ 74%; net yield of both $Dy_3N@C_{80}(Ad)$ isomers 65% (88% if counted on reacted $Dy_3N@C_{80}$), yield of $Dy_3N@C_{80}(Ad)$ isomer based on reacted $Dy_3N@C_{80}$: ~1% for Dy_3-I, 87% for Dy_3-II.

$Sc_3N@C_{80}(Ad)$: conversion of $Sc_3N@C_{80}$ 53%; net yield of both $Sc_3N@C_{80}(Ad)$ isomers 37% (70% if counted on reacted $Sc_3N@C_{80}$), yield of $Sc_3N@C_{80}(Ad)$ isomer based on reacted $Sc_3N@C_{80}$: ~4% for Lu_3-I, 94% for Lu_3-II.

$Lu_3N@C_{80}(Ad)$: conversion of $Lu_3N@C_{80}$ 53%; net yield of both $Lu_3N@C_{80}(Ad)$ isomer 51% (94% if counted on reacted $Lu_3N@C_{80}$), yield of $Lu_3N@C_{80}(Ad)$ isomer based on reacted $Lu_3N@C_{80}$: ~4% for Lu_3-I, 94% for Lu_3-II.

$Y_3N@C_{80}(Ad)$: conversion of $Y_3N@C_{80}$ 53%; net yield of both $Y_3N@C_{80}(Ad)$ isomer 45% (84% if counted on reacted $Y_3N@C_{80}$), yield of $Y_3N@C_{80}(Ad)$ isomer based on reacted $Y_3N@C_{80}$: ~2% for Y_3-I, 82% for Y_3-II.

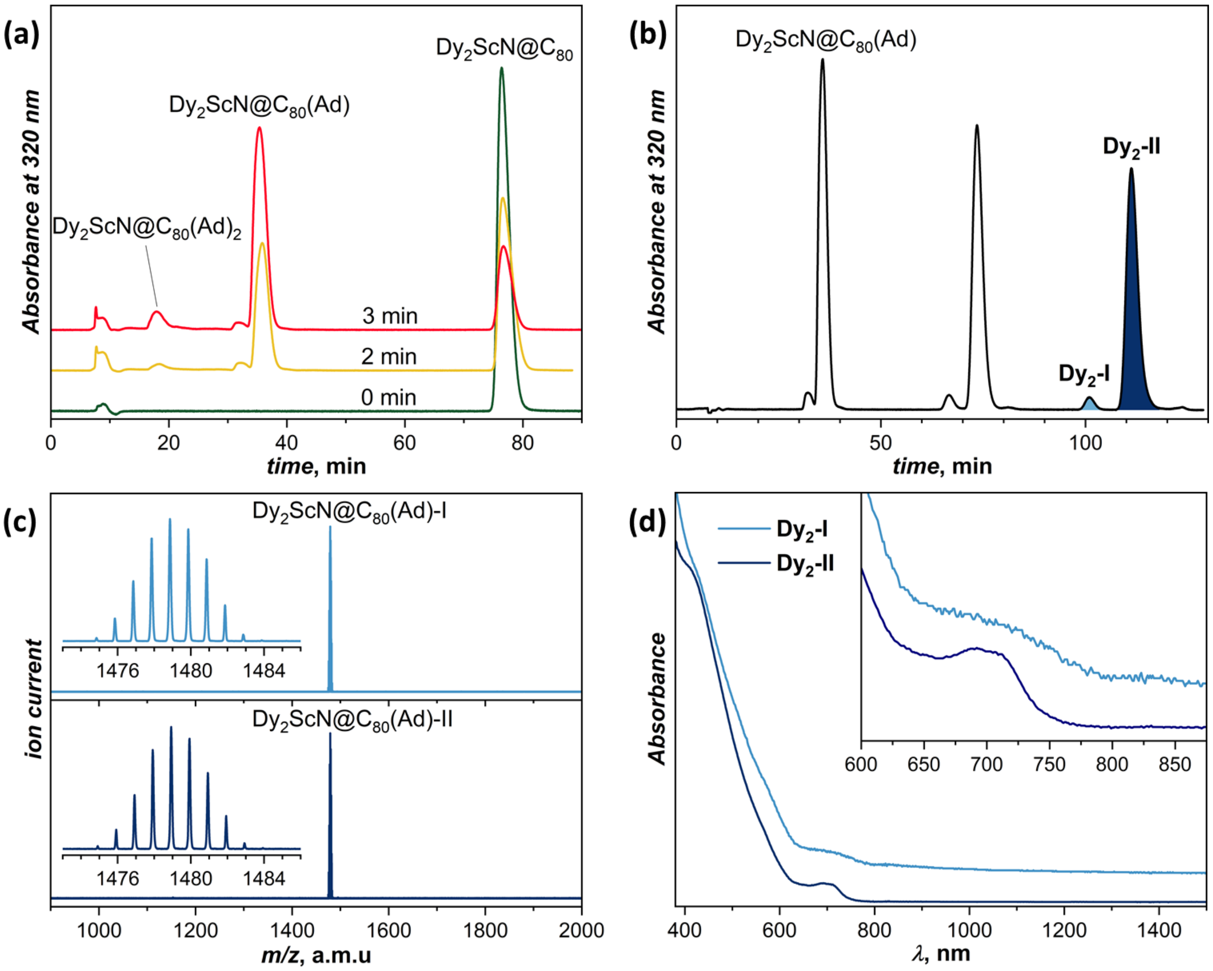

Figure S2. Synthesis and separation of $Dy_2ScN@C_{80}(Ad)$. (a) HPLC traces measured during photochemical reaction between $Dy_2ScN@C_{80}$ and AdN_2. (b) Separation of two isomers with recycling HPLC. (c) MALDI mass-spectra of separated isomers **Dy_2-I** and **Dy_2-II**. (d) Vis-NIR absorption spectra of **Dy_2-I** and **Dy_2-II**.

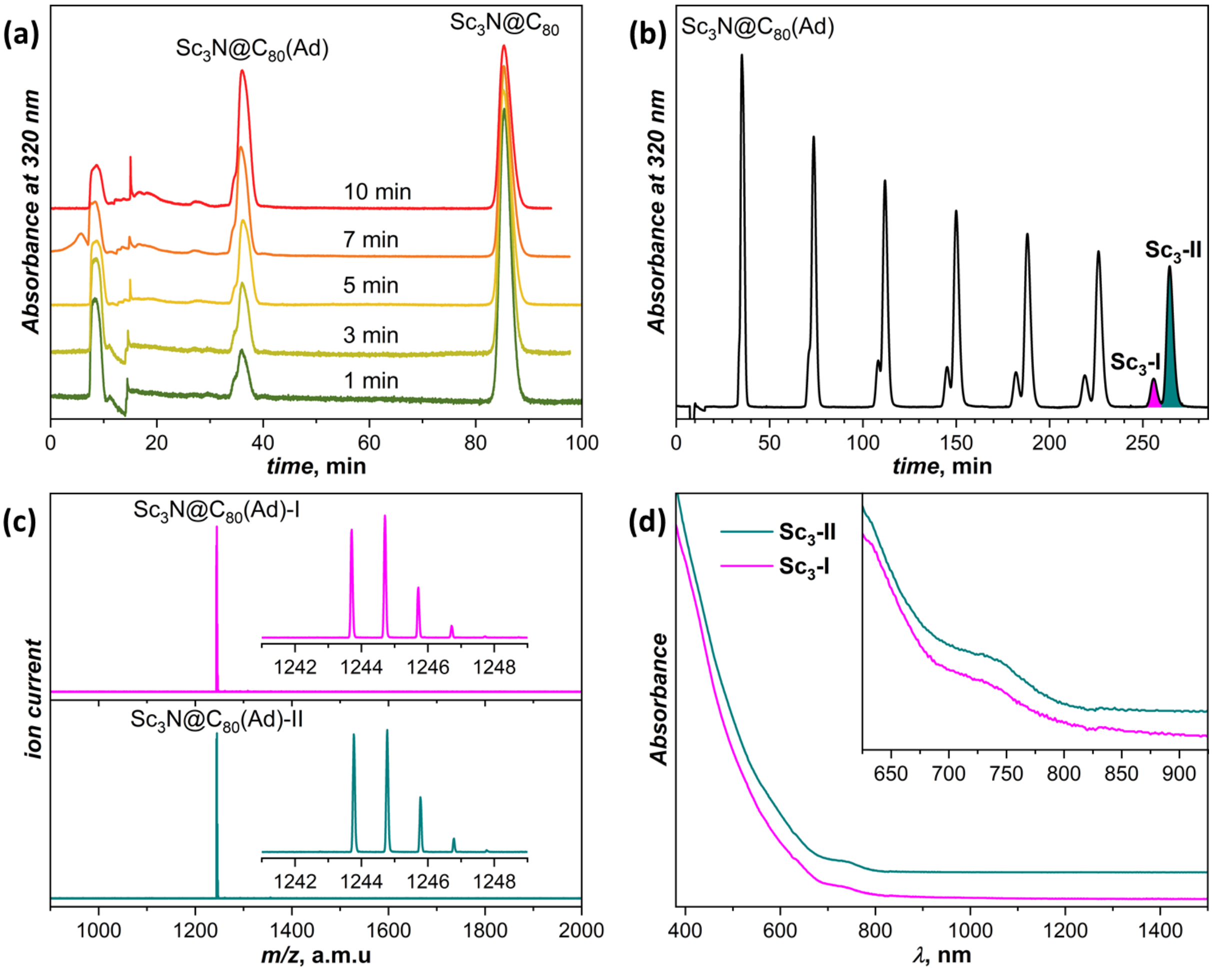

Figure S3. Synthesis and separation of $Sc_3N@C_{80}$(Ad). (a) HPLC traces measured during photochemical reaction between $Sc_3N@C_{80}$ and AdN_2. (b) Separation of two isomers with recycling HPLC. (c) MALDI mass-spectra of separated isomers **Sc_3-I** and **Sc_3-II**. (d) Vis-NIR absorption spectra of **Sc_3-I** and **Sc_3-II**.

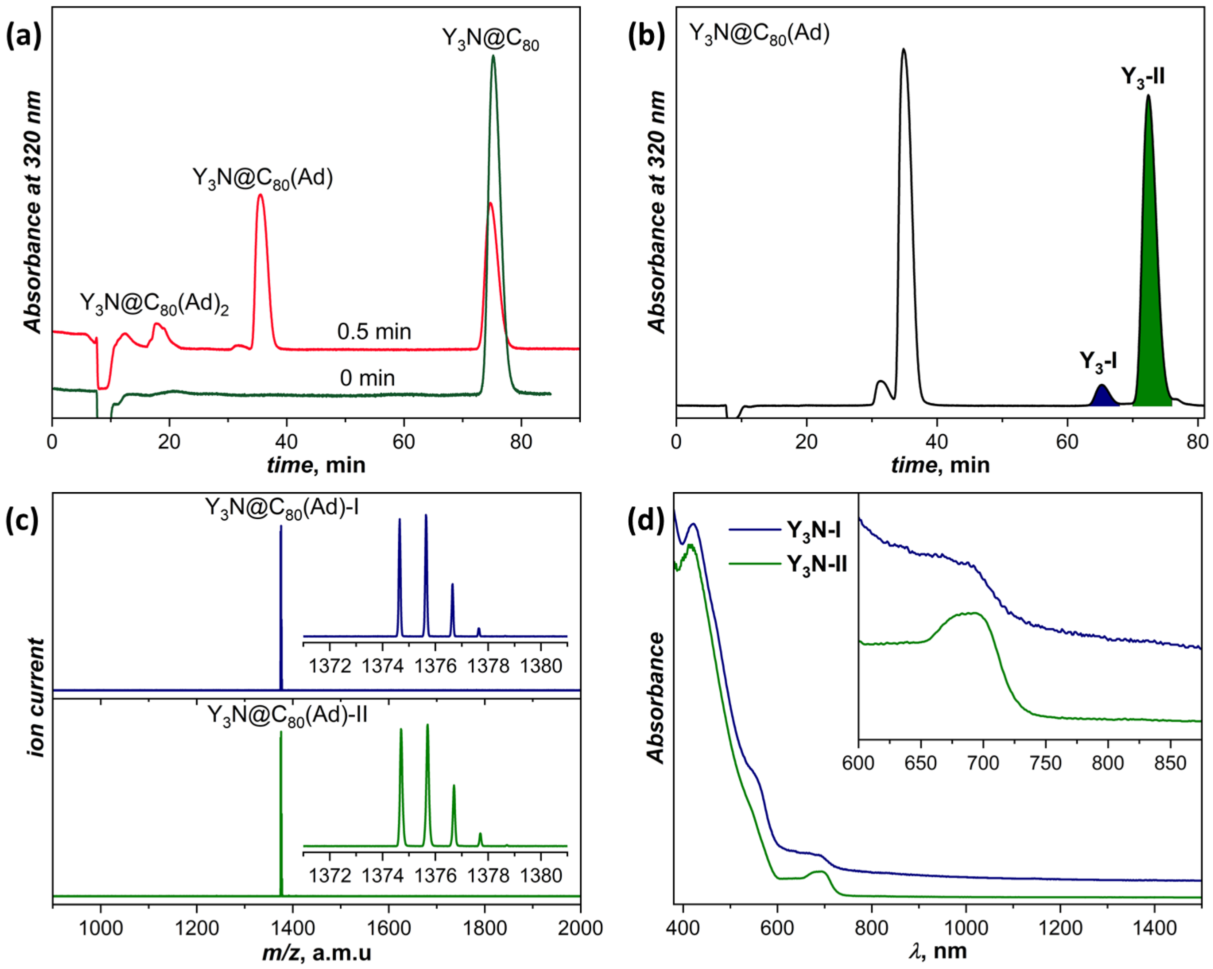

Figure S4. Synthesis and separation of $Y_3N@C_{80}(Ad)$. (a) HPLC traces measured during photochemical reaction between $Y_3N@C_{80}$ and AdN_2. (b) Separation of two isomers with recycling HPLC. (c) MALDI mass-spectra of separated isomers **Y_3-I** and **Y_3-II**. (d) Vis-NIR absorption spectra of **Y_3-I** and **Y_3-II**.

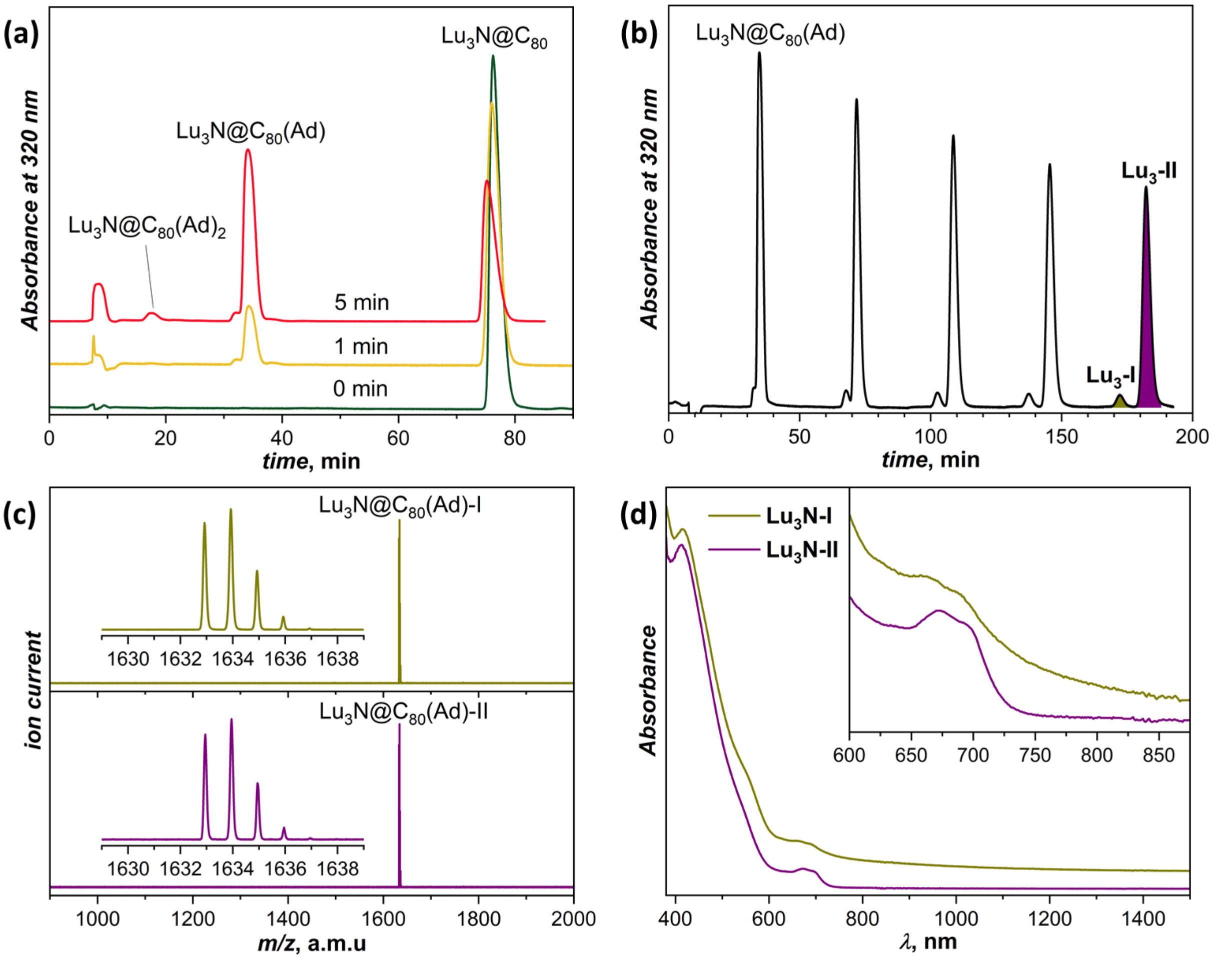

Figure S5. Synthesis and separation of $Lu_3N@C_{80}$(Ad). (a) HPLC traces measured during photochemical reaction between $Lu_3N@C_{80}$ and AdN_2. (b) Separation of two isomers with recycling HPLC. (c) MALDI mass-spectra of separated isomers **Lu_3-I** and **Lu_3-II**. (d) Vis-NIR absorption spectra of **Lu_3-I** and **Lu_3-II**.

^{1}H NMR spectra

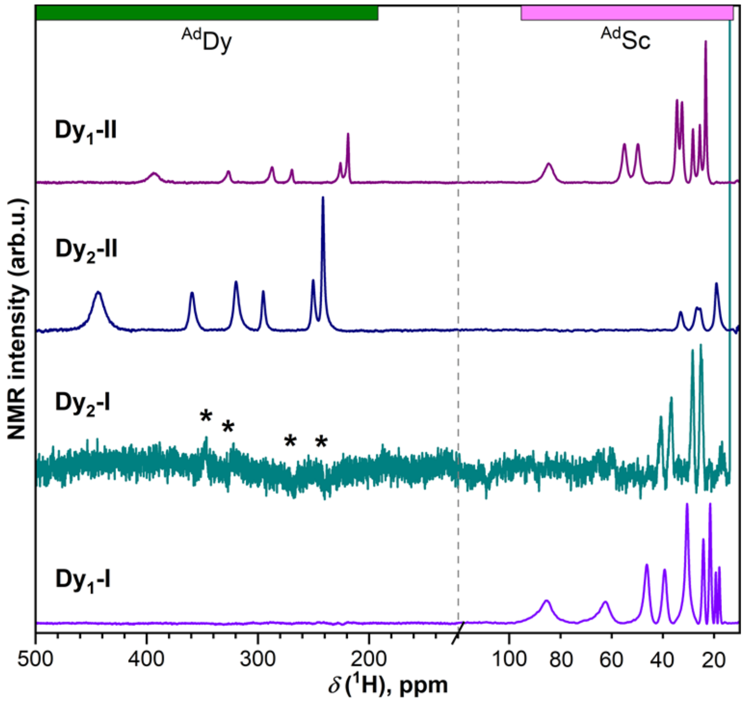

Figure S6. ^{1}H NMR spectra of **Dy$_1$-II**, **Dy$_2$-II**, **Dy$_2$-I**, and **Dy$_1$-I** measured in CS_2 solution at room temperature; note the change of the scale at 120 ppm. **Dy$_1$-II** and **Dy$_2$-II** exhibit signals both in the ^{Ad}Dy range (δ > 200 ppm) and ^{Ad}Sc range (δ < 100 ppm), suggesting that two forms coexist in solution at room temperature. Note that the relative intensity of the ^{Ad}Sc form in **Dy$_1$-II** is considerably higher than in **Dy$_2$-II**. In **Dy$_2$-I**, only four signals in the ^{Ad}Sc range can be distinguished. Some peaks in the ^{Ad}Dy range may be present (marked by "*"), but signal-to-noise ratio is not sufficient to detect them reliably. The yield of **Dy$_2$-I** is 26 times lower than the yield of **Dy$_2$-II** (Figure S2), which limits the sample amount and prevents measuring the spectra of better quality in a reasonable acquisition time. In **Dy$_1$-I**, the ^{Ad}Sc form also dominates as follows from the lack of signals in the ^{Ad}Dy range.

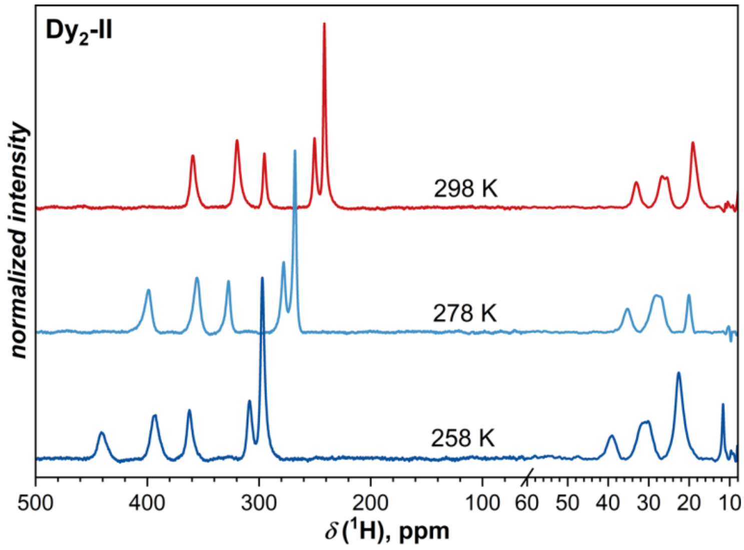

Figure S7. ^{1}H NMR spectra of **Dy$_2$-II** measured at 298, 278, and 258 K; note the change of the scale at 120 ppm. Signals at large positive shifts required different excitation window and were not measured at 278 and 258 K; see Figure 4 in the main text for the spectrum of **Dy$_2$-II** in the whole range.

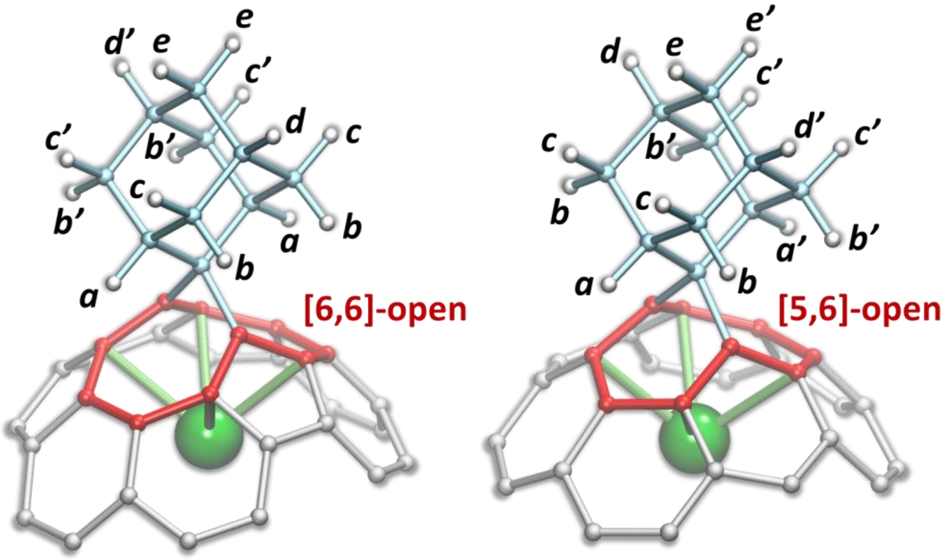

Figure S8. Labeling of protons in the Ad moiety in [6,6]-open and [5,6]-open isomers. Ad moiety has C_{2v} symmetry and five types of protons, 2×***a***, 4×***b***, 4×***c***, 2×***d***, and 2×***e***. When it is attached to the fullerene, its symmetry is reduced to C_s, and remaining symmetry plane has different orientation in [6,6] and [5,6] isomers. As a result, [6,6] isomer has eight types of protons, 2×***a***, 2×***b***, 2×***b'***, 2×***c***, 2×***c'***, ***d***, ***d'***, 2×***e***. [5,6] isomer has nine types of protons, ***a***, ***a'***, 2×***b***, 2×***b'***, 2×***c***, 2×***c'***, 2***d***, ***e***, ***e'***. In this notation, ***b*** and ***b'*** are equivalent in C_{2v} symmetry but become different in C_s.

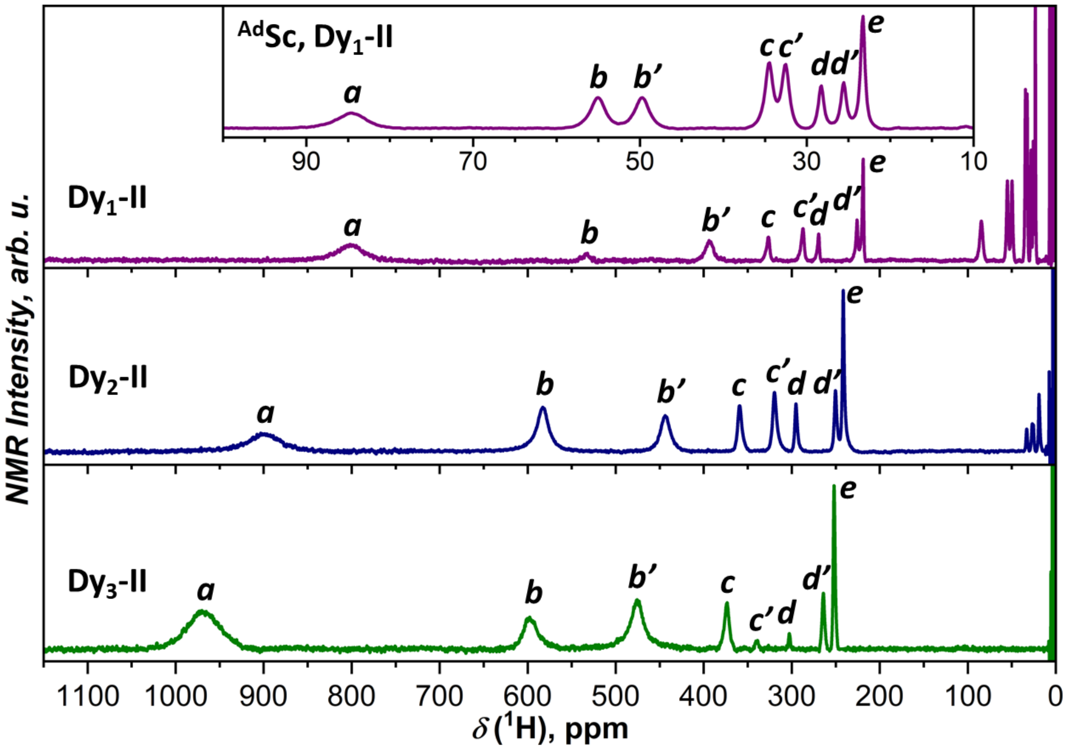

Figure S9. Assignment of proton signals in ^{1}H NMR spectra of [6,6]-$DySc_2N@C_{80}$(Ad) (**Dy₁-II**), [6,6]-$Dy_2ScN@C_{80}$(Ad) (**Dy₂-II**), and [6,6]-$Dy_3N@C_{80}$(Ad) (**Dy₃-II**). T = 298 K, peaks at δ > 200 ppm corresponds to ^{Ad}Dy forms.

Calculations of paramagnetic chemical shifts

For lanthanides with axial magnetic anisotropy, pseudocontact paramagnetic shift, which they induce in other nuclei, can be calculated as:

$$\delta_i^{pc} = \frac{3\cos^2\theta_i - 1}{12\pi R_i^3}\left(\chi_{zz} - \frac{\chi_{xx} + \chi_{yy}}{2}\right) \qquad \text{(S1)}$$

where χ_{ij} are components of the magnetic susceptibility tensor. When $\chi_{xx} \neq \chi_{yy}$, an additional term is added to the formula to account for rhombicity:

$$\delta_i^{pc} = \frac{1}{12\pi R_i^3}\left((3\cos^2\theta_i - 1)\left(\chi_{zz} - \frac{\chi_{xx} + \chi_{yy}}{2}\right) + \frac{3}{2}\sin^2\theta_i\cos(2\varphi_i)\left(\chi_{xx} - \chi_{yy}\right)\right) \qquad \text{(S2)}$$

Here R_i is the distance between lanthanide and nucleus of interest, and θ_i and φ_i are angles in the spherical coordinate system centered on the lanthanide. In particular, θ_i is the angle between quantization axis (principal *z*-axis of the χ tensor) and radius-vector R_i. In the calculations discussed in this section, we used DFT-optimized molecular structures, principal components of the χ^{298} tensor were obtained from CASSCF calculations with OpenMolcas/SINGLE_ANISO as a single-ion magnetic susceptibility tensor computed for *T* = 298 K, while angles θ_i and φ_i were calculated based on principal orientations of the χ^{298} tensor (note that they deviate slightly from principal orientations of the *g*-tensor for KD1); details of DFT and CASSCF calculations for $DySc_2N@C_{80}(Ad)$ were reported in Ref. [15], calculations for $Dy_2ScN@C_{80}(Ad)$ and $Dy_3N@C_{80}(Ad)$ were performed in this work and are described in further sections. Labeling of protons in Ad moieties and assignment of experimental peaks are shown in Figures S8 and S9, respectively. Experimental δ^{para} values are estimated by subtracting from experimental chemical shifts the average diamagnetic chemical shift of Ad protons in $Sc_3N@C_{80}(Ad)$, equal 2.6 ppm.

At first, we analyzed if Eq. S1 and Eq. S2 can predict paramagnetic chemical shifts in ^{Ad}Dy and ^{Ad}Sc forms of $DySc_2N@C_{80}(Ad)$. At room temperature, endohedral M_3N cluster in the $M_3N@C_{80}(Ad)$ molecule rotates around the ^{Ad}M–N bond, which gives effective C_s symmetry observed in NMR measurements. For each $DySc_2N@C_{80}(Ad)$ isomer, DFT calculations localized several conformers with different orientation of the endohedral cluster, which span the range of less than 10 kJ mol^{-1}. Rotation of the cluster can be described as jumps between these structures.

Conformers of [6,6]-^{Ad}Dy $DySc_2N@C_{80}(Ad)$ are shown in Figure S10, their computed chemical shifts and principal components of the χ^{298} tensor are listed in Table S1, while Figure S11 visualizes comparison between computed and experimental δ^{para} values. Chemical shifts computed in axial approximation (Eq. (S1)) for the lowest-energy conformer agree very well with experimental values except for a slight overestimation, which can be corrected for by scaling theoretical values by 0.964. Addition of rhombic term (Eq. S2) produces only insignificant changes, which do not affect the overall agreement. Furthermore, averaging of the computed chemical shifts over conformers using statistical weights, computed from their relative energies, also does not improve the agreement between experiment and theory. Thus, an axial χ^{298} tensor, a coincidence of the principal z-axis with the cluster rotation axis, and a central position of the lanthanide ensure that Eq. S1 gives very good agreement with experiment for the ^{Ad}Dy form, and it is sufficed to use only the lowest-energy conformer.

Situation is more complicated for ^{Ad}Sc forms because rotation of the cluster around the ^{Ad}Sc–N bond changes position of the Dy and orientation of its χ^{298} tensor with respect to the Ad moiety. Chemical shifts, calculated for the main conformer of [6,6]-^{Ad}Sc $DySc_2N@C_{80}(Ad)$ using Eq. S1 do not give a good agreement with experimental spectrum (Table S2, Figure S13a), and the use of Eq. S2 does not change

the results either. Furthermore, averaging over six conformers with corresponding weights gives only a moderate improvement (Figure S13b). Note that the accuracy of the conformer relative energies is not known, and variation of these values may produce significant changes in their weights and hence in predicted averaged values. Besides, static picture may be not sufficient for the description of the cluster dynamics, which would require explicit modelling of the rotation, such as provided by DFT molecular dynamics. However, it can be noticed that deviations from experiment in groups of quasi-equivalent protons, such as ***b*** and ***b'***, have an opposite sign for ***b*** and ***b'*** counterparts (Figure S13a). If the chemical shifts in such groups are averaged (denoted as ⟨***b***⟩), then the excellent correlation between experiment and theory is observed (Figure S13c-d). Apparently, albeit artificial, such averaging appears to be a surprisingly good model for the rotation of the cluster, even when only the single lowest-energy conformer is used (Figure S13c).

For the [5,6]-$DySc_2N@C_{80}$(Ad) isomer, only ^{Ad}Sc form is available in sufficient amounts to produce measurable NMR signals. Similar to its [6,6] counterpart, here calculations for the lowest-energy conformer did not give a good correlation between experiment and theory (Table S3, Figure S15a). However, averaging over six conformers resulted in a much better agreement (Figure S15b). Averaging of chemical shifts for quasi-equivalent protons again improved agreement dramatically, even for just a single conformer (Figure S15c-d). Computed paramagnetic shifts for **Dy_2-I** are overestimated and require scaling by 0.82–0.85.

For calculations of chemical shifts in [6,6]-^{Ad}Dy $Dy_2ScN@C_{80}$ and [6,6]-$Dy_3N@C_{80}$ we used only the lowest-energy conformer of each structure, while contributions from each Dy were computed independently and then summed up. Although this can hardly be expected to give a good description of the contributions of ^{cage}Dy ions, a very good agreement between computed and experimental values was obtained. Averaging over quasi-equivalent protons further improved the correlation, but only marginally. Calculated values are somewhat overestimated, but if contributions of ^{Ad}Dy and ^{cage}Dy are adjusted using fitting coefficients from Figures S11a and S13c, the sum matches experimental values nearly exactly (Figure S16b and S16d). Similar good correspondence between experimental and computed paramagnetic shifts is obtained for $Dy_3N@C_{80}$(Ad) (Figure S17). Analysis of individual contribution shows that the shifts induced by the ^{Ad}Dy ion tend to increase from $DySc_2N$ to Dy_2ScN and then to Dy_3N (Table S4). This trend is caused by a combination of factors, including pushing Dy ion towards Ad moiety when the size of the M_3N cluster increases and a slight increase of anisotropy term in Eq. S1. Contributions of ^{cage}Dy ions to the δ^{para} values decrease accordingly in the same raw. As Dy⋯Dy coupling is small (less than 10 K), we do not observe its contribution in room-temperature NMR spectra.

Calculations for ^{Ad}Sc forms of [5,6] and [6,6]-$Dy_2ScN@C_{80}$(Ad) isomers give more ambiguous results (Table S5), suggesting that the explicit consideration of rotational dynamics might be necessary to proper account for the influence of two ^{cage}Dy ions. Experimental chemical shifts do not exceed 33–41 ppm, whereas calculations predict larger values. However, since not all expected experimental signals could be identified in the spectra, further discussion appears premature.

Table S1. Paramagnetic chemical shifts of Ad protons computed for different conformers of [6,6]-^{Ad}Dy DySc$_2$N@C$_{80}$(Ad) (**Dy$_1$-II**), T = 298 K.

	[6,6]-^{Ad}Dy_01	[6,6]-^{Ad}Dy_02	[6,6]-^{Ad}Dy_03	[6,6]-^{Ad}Dy_04	[6,6]-^{Ad}Dy_06	[6,6]-^{Ad}Dy_07	δ^{para} aver.	δ^{para} exp.
**Δ*E*, kJ mol^{-1}**	0.00	3.60	4.67	5.11	6.91	8.03		
weight	0.66	0.15	0.10	0.04	0.02	0.03		
**χ_x, cm^3 mol^{-1}**	0.013	0.013	0.014	0.014	0.011	0.012		
**χ_y, cm^3 mol^{-1}**	0.016	0.015	0.015	0.015	0.016	0.016		
**χ_z, cm^3 mol^{-1}**	0.111	0.111	0.111	0.111	0.112	0.112		
**$(\chi_z - 0.5\{\chi_x+\chi_y\})$**	0.096	0.097	0.097	0.097	0.098	0.098		
**δ^{para}, Eq. 1**								
a	823.6	837.3	818.4	777.9	838.9	813.4	823.3	**799.3**
b	540.2	464.0	549.8	619.0	467.3	531.6	531.1	**530.9**
b'	424.6	511.1	408.7	312.8	505.0	410.9	432.9	**390.9**
c	333.5	314.6	334.7	346.6	315.8	328.0	330.7	**324.2**
c'	301.3	328.6	295.6	260.5	326.5	294.3	303.5	**285.0**
d	274.6	251.4	276.5	295.6	252.5	269.4	271.5	**267.0**
d'	238.5	267.8	232.7	198.2	265.0	231.6	241.1	**223.2**
e	226.6	229.6	224.9	217.3	229.0	221.6	226.4	**216.5**
**δ^{para}, Eq. 2**								
a	829.7	842.1	820.9	775.3	853.8	824.1	828.8	**799.3**
b	539.0	463.0	550.9	619.0	463.0	530.1	530.1	**530.9**
b'	422.9	509.9	409.8	314.4	502.3	407.8	431.7	**390.9**
c	334.1	315.1	335.2	346.2	317.0	329.4	331.3	**324.2**
c'	301.6	329.0	296.1	260.6	328.2	295.2	304.0	**285.0**
d	274.2	251.0	276.7	295.6	251.2	269.0	271.2	**267.0**
d'	238.0	267.4	233.0	198.8	264.1	230.6	240.7	**223.2**
e	226.6	229.7	225.0	217.3	229.2	221.8	226.5	**216.5**

"Δ*E*" are DFT-computed relative energies of conformers, "weight" for a given conformer is calculated based on its relative energy and symmetry, χ values are computed *ab initio* for T = 298 K and are given in SGI system, "δ^{para} aver." are chemical shifts averaged over conformers, "δ^{para} exp." are obtained by subtracting 2.6 ppm from experimental chemical shifts, where 2.6 ppm is an average diamagnetic shift of Ad protons.

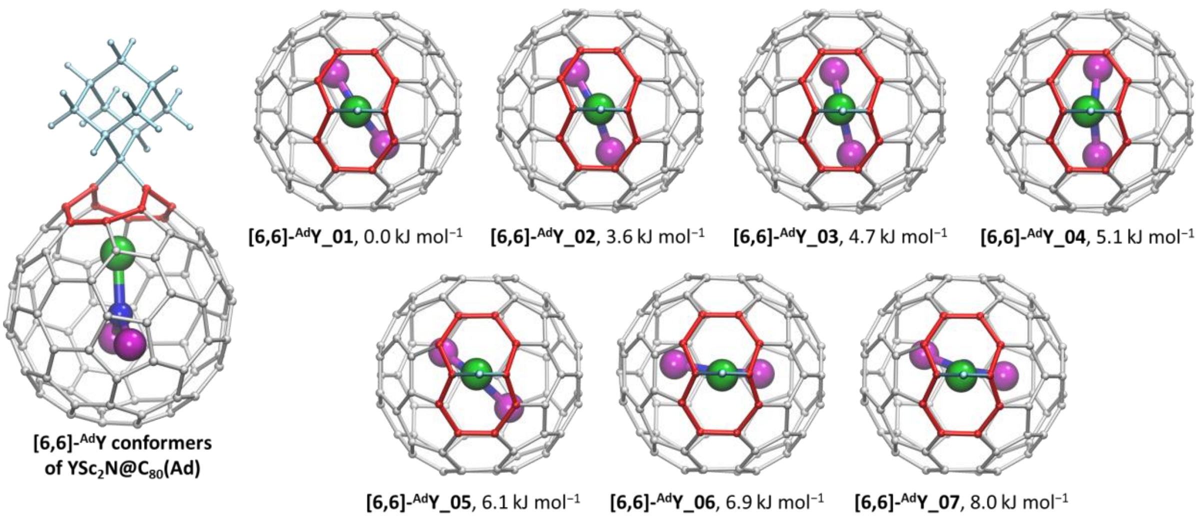

Figure S10. Molecular structure of seven lowest-energy [6,6]-^{Ad}Y conformers of $YSc_2N@C_{80}$(Ad) and their relative energies (PBE/TZ2P level) from Ref. [15]. These structures were then re-optimized with Dy instead of Y and used in calculations of chemical shifts (Table S1). CASSCF calculations for conformer [6,6]-AdDy_05 did not converge, and averaging in chemical shift calculations was done over six conformers.

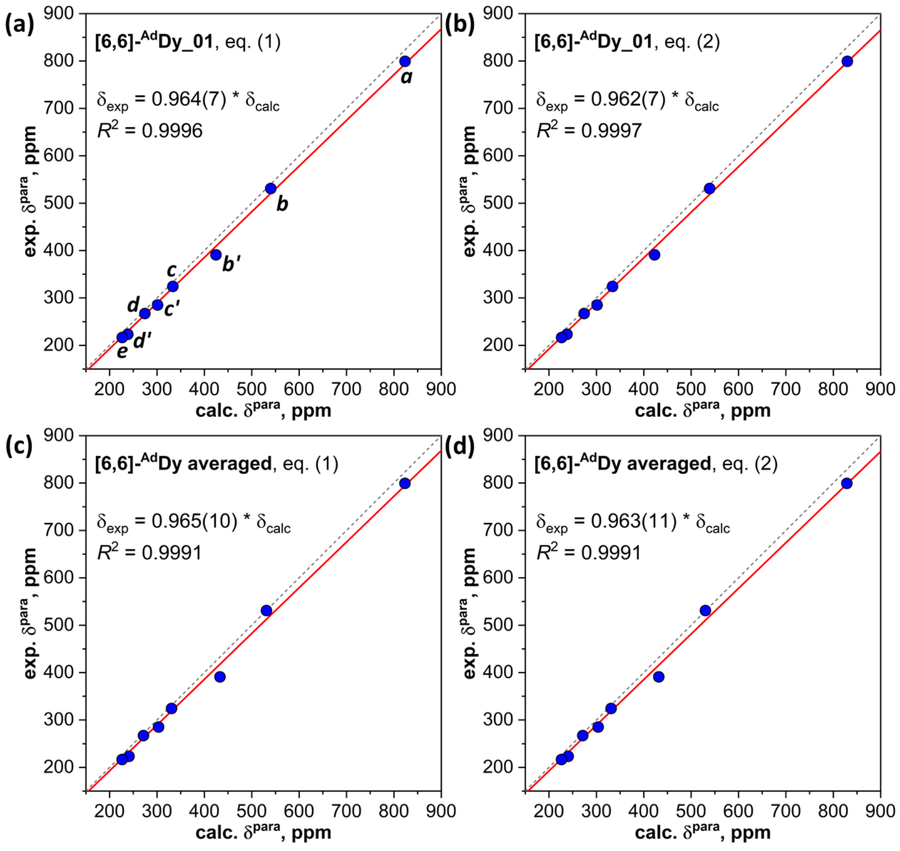

Figure S11. Experimental versus calculated paramagnetic chemical shifts in [6,6]-^{Ad}Dy $DySc_2N@C_{80}$(Ad), T = 298 K. (a) The lowest energy conformer, axial approximation (Eq. S1). (b) The lowest energy conformer, Eq. S2. (c) Averaging over six conformers (Figure S10), Eq. S1. (b) Averaging over six conformers, Eq. S2. In each plot, dashed line is $\delta_{exp} = \delta_{calc}$, red line is a linear fit for $\delta_{exp} = const*\delta_{calc}$.

Table S2. Paramagnetic chemical shifts of Ad protons computed for different conformers of [6,6]-^{Ad}Sc $DySc_2N@C_{80}(Ad)$ (**Dy_1-II**), T = 298 K.

	[6,6]-^{Ad}Sc_01	[6,6]-^{Ad}Sc_02	[6,6]-^{Ad}Sc_03	[6,6]-^{Ad}Sc_04	[6,6]-^{Ad}Sc_05	[6,6]-^{Ad}Sc_06	δ^{para} aver.	δ^{para} exp.
**ΔE, kJ mol^{-1}**	0.00	3.94	5.04	5.77	6.73	9.28		
weight	0.70	0.07	0.09	0.07	0.05	0.02		
**χ_x, cm^3 mol^{-1}**	0.009	0.009	0.008	0.010	0.009	0.009		
**χ_y, cm^3 mol^{-1}**	0.012	0.010	0.010	0.012	0.012	0.012		
**χ_z, cm^3 mol^{-1}**	0.118	0.119	0.119	0.117	0.118	0.118		
**$(\chi_z - 0.5\{\chi_x+\chi_y\})$**	0.107	0.110	0.110	0.106	0.107	0.107		
**δ^{para}, Eq. 1**								
a	84.0	45.7	99.5	105.2	82.1	94.8	84.2	**82.0**
b	92.3	96.8	64.6	8.4	9.0	1.7	78.9	**52.4**
b'	13.6	−69.5	71.8	122.8	97.3	112.9	26.0	**47.2**
c	48.0	37.2	42.6	23.6	17.4	18.2	43.1	**31.9**
c'	19.8	−21.1	45.0	64.9	48.5	58.2	24.2	**30.0**
d	44.9	40.7	34.2	9.1	7.0	4.7	38.7	**25.7**
d'	8.9	−31.5	37.2	61.5	46.3	55.5	14.7	**23.0**
e	23.1	4.2	30.5	30.5	22.6	26.1	23.0	**20.7**
a	84.0	45.7	99.5	105.2	82.1	94.8	84.2	**82.0**
**⟨*b*⟩**	53.0	13.6	68.2	65.6	53.1	57.3	52.5	**49.8**
**⟨*c*⟩**	33.9	8.0	43.8	44.3	32.9	38.2	33.7	**31.0**
**⟨*d*⟩**	26.9	4.6	35.7	35.3	26.7	30.1	26.7	**24.4**
e	23.1	4.2	30.5	30.5	22.6	26.1	23.0	**20.7**
**δ^{para}, Eq. 2**								
a	85.4	45.8	98.3	104.4	84.4	92.9	85.1	**82.0**
b	93.3	96.8	63.4	7.9	9.8	1.1	79.5	**52.4**
b'	15.8	−69.4	71.8	122.4	98.5	111.9	27.6	**47.2**
c	48.9	37.3	41.9	23.2	18.3	17.5	43.7	**31.9**
c'	21.1	−21.1	44.7	64.5	49.5	57.4	25.2	**30.0**
d	45.5	40.8	33.6	8.8	7.5	4.3	39.1	**25.7**
d'	10.0	−31.4	37.2	61.2	47.1	54.9	15.5	**23.0**
e	23.8	4.3	30.1	30.2	23.3	25.6	23.4	**20.7**
a	85.4	45.8	98.3	104.4	84.4	92.9	85.1	**82.0**
**⟨*b*⟩**	54.6	13.7	67.6	65.2	54.2	56.5	53.6	**49.8**
**⟨*c*⟩**	35.0	8.1	43.3	43.9	33.9	37.4	34.4	**31.0**
**⟨*d*⟩**	27.7	4.7	35.4	35.0	27.3	29.6	27.3	**24.4**
e	23.8	4.3	30.1	30.2	23.3	25.6	23.4	**20.7**

"ΔE" are DFT-computed relative energies of conformers, "weight" for a given conformer is calculated based on its relative energy and symmetry, χ values are computed *ab initio* for T = 298 K and are given in CGS system, "δ^{para} aver." are chemical shifts averaged over conformers, "δ^{para} exp." are obtained by subtracting 2.6 ppm from experimental chemical shifts, where 2.6 ppm is an average diamagnetic shift of Ad protons; in averaged shifts of quasi-equivalent protons (***b*** and ***b'***, ***c*** and ***c'***, ***d*** and ***d'***) are denoted as ⟨***b***⟩, ⟨***c***⟩, and ⟨***d***⟩.

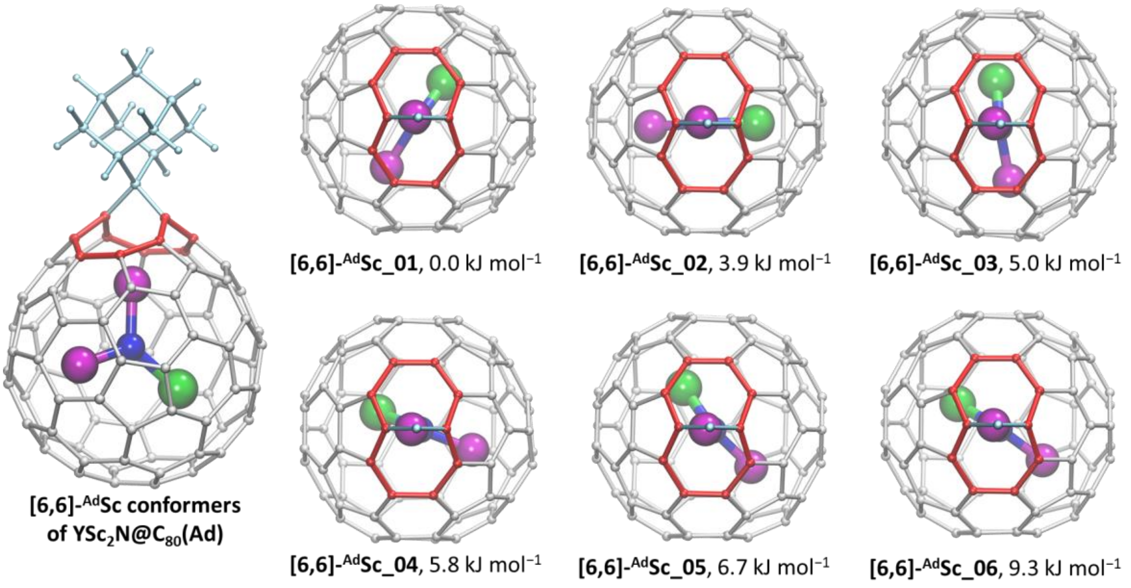

Figure S12. Molecular structure of six lowest-energy [6,6]-^{Ad}Sc conformers of $YSc_2N@C_{80}$(Ad) and their relative energies (PBE/TZ2P level) from Ref. [15]. These structures were then re-optimized with Dy instead of Y and used in calculations of chemical shifts (Table S2).

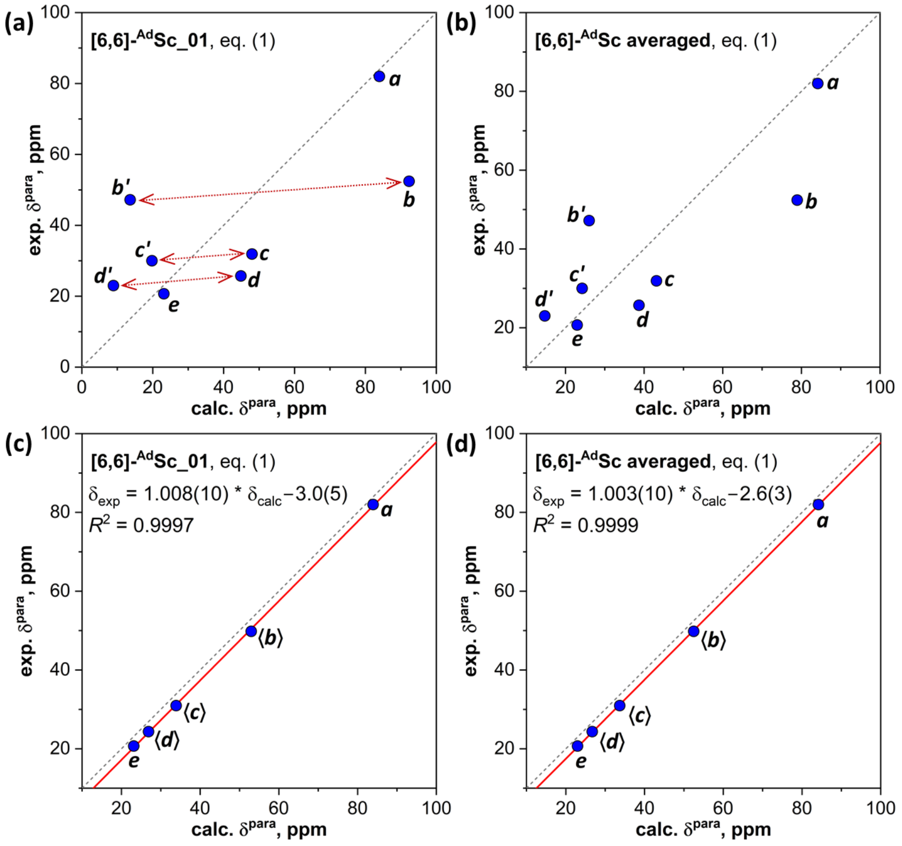

Figure S13. Experimental *versus* calculated paramagnetic chemical shifts in [6,6]-^{Ad}Sc $DySc_2N@C_{80}$(Ad), T = 298 K. (a) The lowest energy conformer, Eq. S1. (b) Averaging over six conformers (Figure S12), Eq. S1. (c) The lowest energy conformer, Eq. S1, and averaging in ***b*/*b'***, ***c*/*c'*** and ***d*/*d'*** pairs (averaged values are denoted as ⟨***b***⟩, ⟨***c***⟩, ⟨***d***⟩). (d) Averaging over six conformers, Eq. S1, and averaging in ***b*/*b'***, ***c*/*c'*** and ***d*/*d'*** pairs. In each plot, dashed line is $\delta_{exp} = \delta_{calc}$, red line is a linear fit for $\delta_{exp} = c_1 * \delta_{calc} + c_2$.

Table S3. Paramagnetic chemical shifts of Ad protons computed for different conformers of [5,6]-^{Ad}Sc $DySc_2N@C_{80}(Ad)$ (**Dy$_1$-I**), T = 298 K.

	[5,6]-^{Ad}Sc_01	[5,6]-^{Ad}Sc_02	[5,6]-^{Ad}Sc_03	[5,6]-^{Ad}Sc_04	[5,6]-^{Ad}Sc_05	[5,6]-^{Ad}Sc_06	δ^{para} aver.	δ^{para} exp.
**Δ*E*, kJ mol^{-1}**	0.00	1.10	1.60	2.59	2.98	7.25		
weight	0.35	0.22	0.18	0.12	0.10	0.02		
**χ_x, cm^3 mol^{-1}**	0.009	0.009	0.009	0.009	0.009	0.009		
**χ_y, cm^3 mol^{-1}**	0.011	0.011	0.011	0.012	0.011	0.011		
**χ_z, cm^3 mol^{-1}**	0.118	0.118	0.118	0.117	0.119	0.118		
**($\chi_z - 0.5\{\chi_x+\chi_y\}$)**	0.108	0.108	0.108	0.107	0.109	0.108		
**δ^{para}, Eq. 1**								
a	178.7	88.9	45.5	15.8	99.9	121.7	105.1	**82.9**
a'	8.9	81.5	176.4	174.1	20.8	39.6	77.7	**59.8**
b	96.3	51.3	45.5	22.6	45.5	61.5	62.0	**43.7**
b'	29.8	46.2	94.5	53.8	14.9	29.7	46.7	**36.8**
c	74.7	35.5	22.2	22.2	34.8	46.5	45.2	**28.0**
c'	3.9	29.4	73.4	101.3	2.7	13.1	34.3	**21.6**
d	32.0	25.2	37.7	31.4	13.9	23.0	29.4	**19.0**
e	35.6	22.7	26.8	19.3	15.9	23.9	26.8	**16.8**
e'	18.6	21.0	38.9	34.5	8.2	16.0	23.7	**15.4**
**⟨*a*⟩**	93.8	85.2	111.0	95.0	60.3	80.6	91.4	**71.4**
**⟨*b*⟩**	63.1	48.7	70.0	38.2	30.2	45.6	54.3	**40.2**
**⟨*c*⟩**	39.3	32.4	47.8	61.8	18.7	29.8	39.7	**24.8**
d	32.0	25.2	37.7	31.4	13.9	23.0	29.4	**19.0**
**⟨*e*⟩**	27.1	21.8	32.9	26.9	12.1	19.9	25.3	**16.1**
**δ^{para}, Eq. 2**								
a	179.0	88.3	42.4	17.5	98.2	120.7	104.5	**82.9**
a'	7.6	80.5	176.1	173.3	19.1	38.4	76.7	**59.8**
b	96.0	50.5	43.7	21.7	43.8	60.4	61.1	**43.7**
b'	29.2	45.2	93.9	54.3	13.0	28.4	45.9	**36.8**
c	74.6	35.0	20.7	21.2	33.7	45.9	44.6	**28.0**
c'	3.5	28.8	73.1	102.1	1.6	12.4	33.9	**21.6**
d	31.8	24.7	37.0	31.2	12.9	22.3	28.9	**19.0**
e	35.5	22.3	26.1	19.4	15.1	23.4	26.5	**16.8**
e'	18.4	20.6	38.4	34.4	7.4	15.4	23.3	**15.4**
**⟨*a*⟩**	93.3	84.4	109.2	95.4	58.7	79.5	90.6	**71.4**
**⟨*b*⟩**	62.6	47.9	68.8	38.0	28.4	44.4	53.5	**40.2**
**⟨*c*⟩**	39.0	31.9	46.9	61.6	17.7	29.1	39.2	**24.8**
d	31.8	24.7	37.0	31.2	12.9	22.3	28.9	**19.0**
**⟨*e*⟩**	26.9	21.5	32.2	26.9	11.2	19.4	24.9	**16.1**

"Δ*E*" are DFT-computed relative energies of conformers, "weight" for a given conformer is calculated based on its relative energy and symmetry, χ values are computed *ab initio* for T = 298 K and are given in CGS system, "δ^{para} aver." are chemical shifts averaged over conformers, "δ^{para} exp." are obtained by subtracting 2.6 ppm from experimental chemical shifts, where 2.6 ppm is an average diamagnetic shift of Ad protons; in averaged shifts of quasi-equivalent protons (***a*** and ***a'***, ***b*** and ***b'***, ***c*** and ***c'***, ***e*** and ***e'***) are denoted as ⟨***a***⟩, ⟨***b***⟩, ⟨***c***⟩, and ⟨***e***⟩.

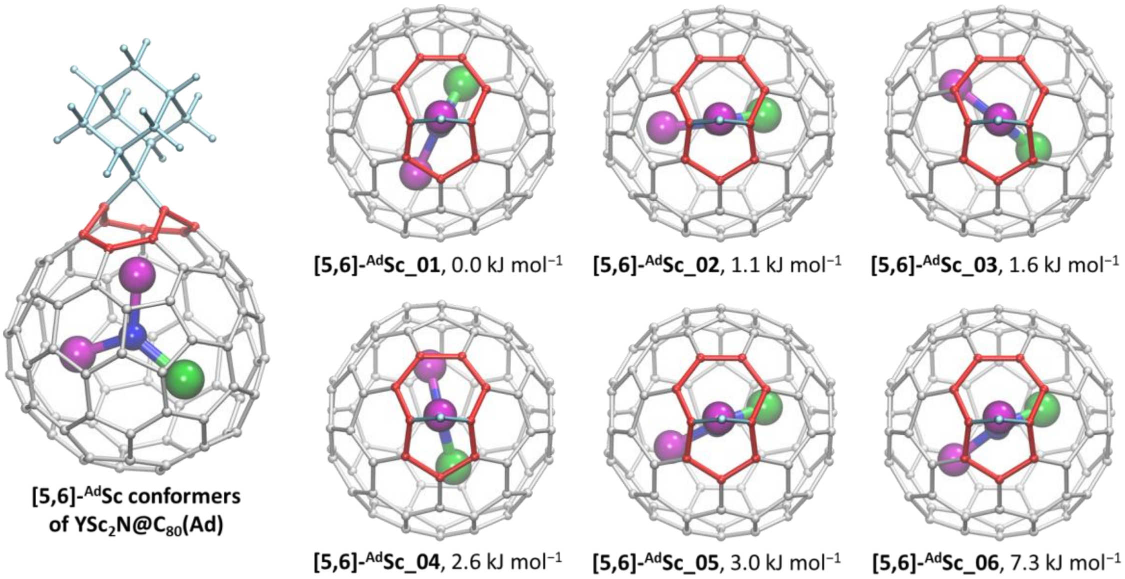

Figure S14. Molecular structure of six unique [5,6]-^{Ad}Sc conformers of $YSc_2N@C_{80}(Ad)$ and their relative energies (PBE/TZ2P level) from Ref. [15]. These structures were then re-optimized with Dy instead of Y and used in calculations of chemical shifts (Table S3).

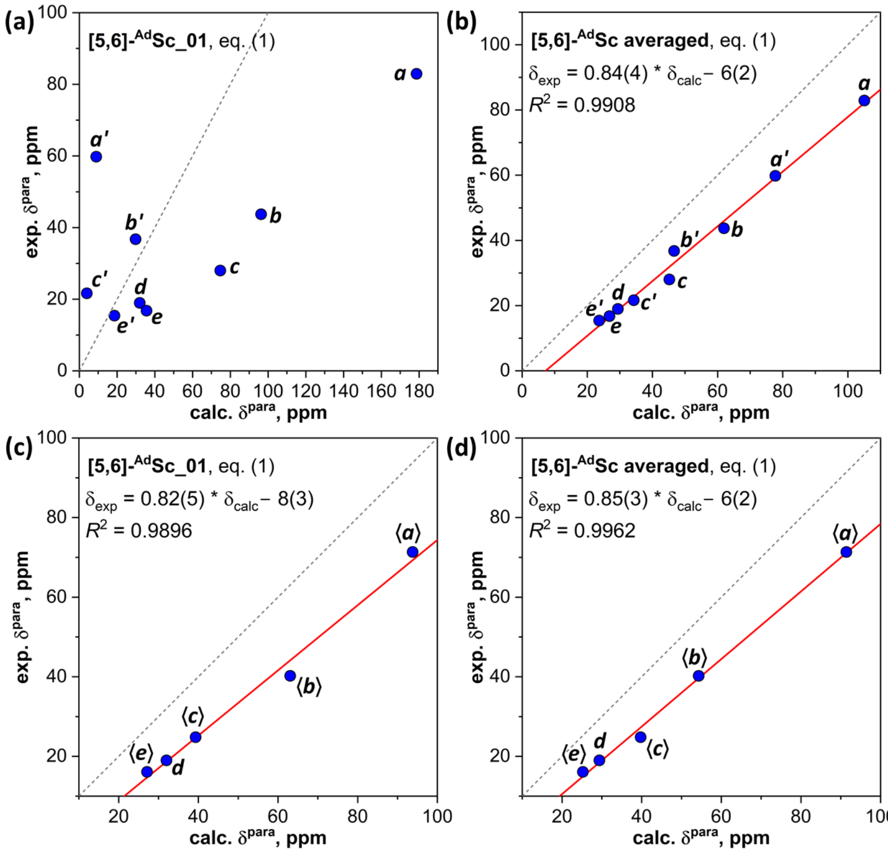

Figure S15. Experimental *versus* calculated paramagnetic chemical shifts in [5,6]-^{Ad}Sc $DySc_2N@C_{80}(Ad)$, T = 298 K. (a) The lowest energy conformer, Eq. S1. (b) Averaging over six conformers (Fig. S14), Eq. S1. (c) The lowest energy conformer, Eq. S1, and averaging in ***a*/*a'***, ***b*/*b'***, ***c*/*c'*** and ***e*/*e'*** pairs (averaged values are denoted as ⟨***a***⟩, ⟨***b***⟩, ⟨***c***⟩, ⟨***e***⟩). (d) Averaging over six conformers, Eq. S1, and averaging in ***a*/*a'***, ***b*/*b'***, ***c*/*c'*** and ***e*/*e'*** pairs. In each plot, dashed line is $\delta_{exp} = \delta_{calc}$, red line is a linear fit $\delta_{exp} = c_1 * \delta_{calc} + c_2$.

Table S4. Paramagnetic chemical shifts of Ad protons computed for [6,6]-$Dy_2ScN@C_{80}$(Ad) (**Dy_2-II**) and [6,6]-$Dy_3N@C_{80}$(Ad) (**Dy_3-II**), T = 298 K.

	^{Ad}Dy	^{cage}Dy-1	^{cage}Dy-2	δ^{para} sum.	δ^{para} sum.*	δ^{para} exp.	^{Ad}Dy Dy_1-II
**<u>$Dy_2ScN@C_{80}$(Ad)</u>**							
χ_x, cm^3 mol^{-1}	0.010	0.009					0.013
χ_y, cm^3 mol^{-1}	0.016	0.010					0.016
χ_z, cm^3 mol^{-1}	0.112	0.118					0.111
$(\chi_z - 0.5\{\chi_x+\chi_y\})$	0.099	0.109					0.096
δ^{para}, Eq. 1							
a	856.7	74.9		931.6	898.4	**898.9**	823.6
b	466.6	106.8		573.5	554.5	**582.5**	540.2
b'	526.2	–21.8		504.4	482.3	**442.5**	424.6
c	319.6	51.6		371.2	357.1	**358.8**	333.5
c'	337.1	5.1		342.2	327.1	**319.6**	301.3
d	254.7	51.4		306.0	294.3	**295.6**	274.6
d'	275.1	–7.3		267.8	254.8	**249.8**	238.5
e	234.5	19.1		253.6	242.3	**241.1**	226.6
a	856.7	74.9		931.6	898.4	**898.9**	
⟨***b***⟩	496.4	42.5		538.9	518.4	**512.5**	
⟨***c***⟩	328.3	28.4		356.7	342.1	**339.2**	
⟨***d***⟩	264.9	22.0		286.9	274.5	**272.7**	
e	234.5	19.1		253.6	242.3	**241.1**	
**<u>$Dy_3N@C_{80}$(Ad)</u>**							
χ_x, cm^3 mol^{-1}	0.011	0.008	0.008				
χ_y, cm^3 mol^{-1}	0.015	0.010	0.010				
χ_z, cm^3 mol^{-1}	0.112	0.118	0.118				
$(\chi_z - 0.5\{\chi_x+\chi_y\})$	0.099	0.109	0.109				
δ^{para}, Eq. 1							
a	877.2	27.8	73.3	975.3	941.5	**968.9**	
b	555.1	–28.1	89.6	613.6	591.1	**596.5**	
b'	467.7	56.4	–1.0	520.2	500.8	**475.5**	
c	349.8	–8.6	44.1	382.4	367.1	**373.8**	
c'	325.7	20.7	11.6	355.0	340.5	**339.6**	
d	286.6	–14.4	42.5	311.7	298.6	**302.6**	
d'	259.7	22.8	1.1	280.5	268.4	**263.8**	
e	241.7	3.5	18.7	260.9	249.4	**251.1**	
a	877.2	27.8	73.3	978.3	941.5	**968.9**	
⟨***b***⟩	511.4	14.2	44.3	569.9	546.0	**536.0**	
⟨***c***⟩	337.8	6.0	27.9	371.7	353.8	**356.7**	
⟨***d***⟩	273.1	4.2	21.8	299.1	283.5	**283.2**	
e	241.7	3.5	18.7	263.9	249.4	**251.1**	

χ values are computed *ab initio* for T = 298 K and are given in CGS system, "δ^{para} sum" are chemical shifts obtained as direct sum of two or three components (for Dy_2ScN and Dy_3N, respectively), "δ^{para} sum*" are obtained by summation of the values, scaled using linear fit parameters from Figures S11a and S13c; in averaged shifts of quasi-equivalent protons (***b*** and ***b'***, ***c*** and ***c'***, ***d*** and ***d'***) are denoted as ⟨***b***⟩, ⟨***c***⟩, and ⟨***d***⟩.

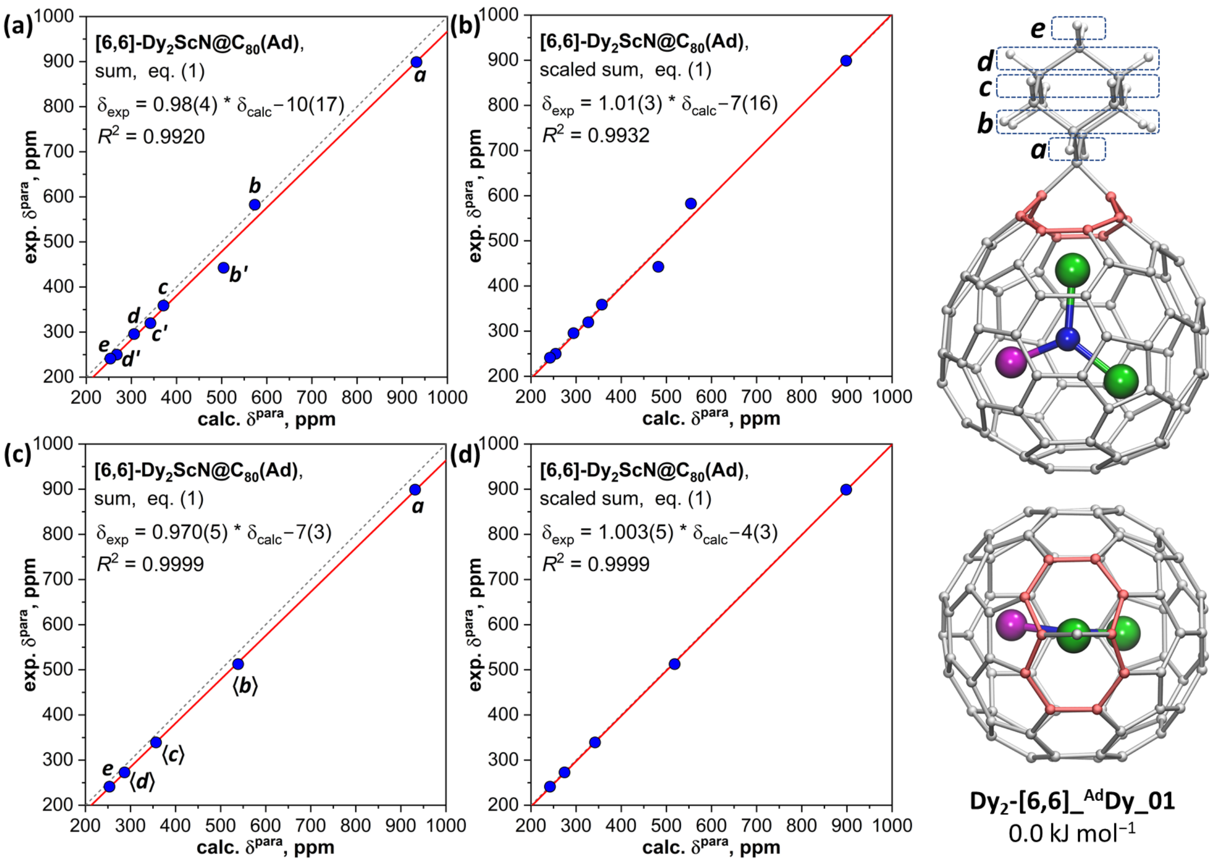

Figure S16. Experimental *versus* calculated paramagnetic chemical shifts in [6,6]-^{Ad}Dy $Dy_2ScN@C_{80}$(Ad), T = 298 K, all calculations for the lowest-energy conformer. (a) Sum of contributions from two Dy ions. (b) Sum of contributions from two Dy ions, each scaled using fitting coefficients from Figures S11a (for ^{Ad}Dy) and S13c (for ^{cage}Dy). (c) Sum of contributions from two Dy ions and averaging in ***b*/*b'***, ***c*/*c'*** and ***d*/*d'*** pairs (averaged values are denoted as ⟨***b***⟩, ⟨***c***⟩, ⟨***d***⟩). (d) Sum of contributions from two Dy ions, each scaled using fitting coefficients from Figures S11a (for ^{Ad}Dy) and S13c (for ^{cage}Dy), and averaging in ***b*/*b'***, ***c*/*c'*** and ***d*/*d'*** pairs. In each plot, dashed line is $\delta_{exp} = \delta_{calc}$, red line is a linear fit $\delta_{exp} = c_1 * \delta_{calc} + c_2$.

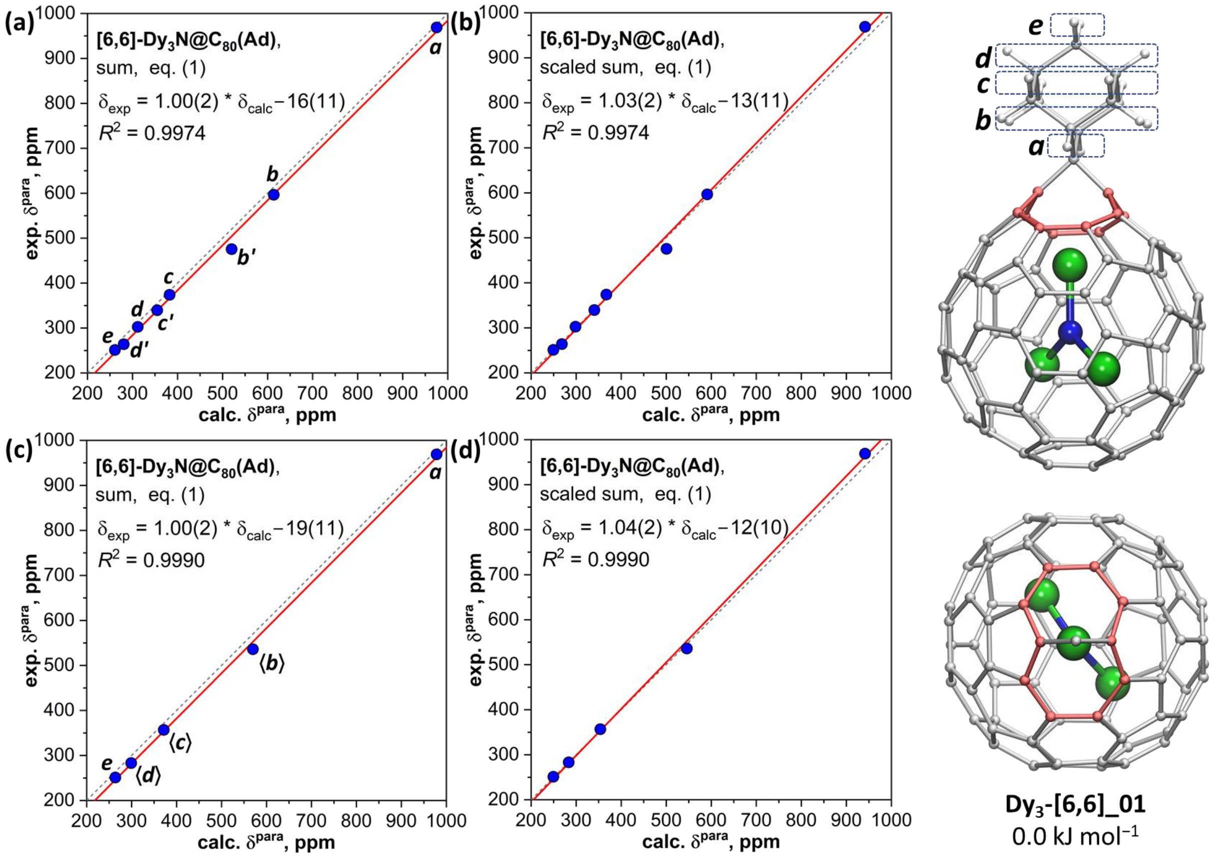

Figure S17. Experimental *versus* calculated paramagnetic chemical shifts in [6,6]-$Dy_3N@C_{80}$(Ad), T = 298 K, all calculations for the lowest-energy conformer. (a) Sum of contributions from two Dy ions. (b) Sum of contributions from two Dy ions, each scaled using fitting coefficients from Figures S11a (for ^{Ad}Dy) and S13c (for ^{cage}Dy). (c) Sum of contributions from two Dy ions and averaging in ***b***/***b'***, ***c***/***c'*** and ***d***/***d'*** pairs (averaged values are denoted as ⟨***b***⟩, ⟨***c***⟩, ⟨***d***⟩). (d) Sum of contributions from two Dy ions, each scaled using fitting coefficients from Figures S11a (for ^{Ad}Dy) and S13c (for ^{cage}Dy), and averaging in ***b***/***b'***, ***c***/***c'*** and ***d***/***d'*** pairs. In each plot, dashed line is $\delta_{exp} = \delta_{calc}$, red line is a linear fit for $\delta_{exp} = c_1 * \delta_{calc} + c_2$.

Table S5. Paramagnetic chemical shifts of Ad protons computed for [6,6]-^{Ad}Sc $Dy_2ScN@C_{80}$(Ad) (**Dy_2-II-^{Ad}Sc**) and [5,6]-^{Ad}Sc $Dy_2ScN@C_{80}$(Ad) (**Dy_2-I**), *T* = 298 K.

	^{cage}**Dy-1**	^{cage}**Dy-2**	δ^{para} **sum.**	δ^{para} **sum.***	δ^{para} **exp. Dy_2**	δ^{para} **exp. Dy_1**
**[6,6]-^{Ad}Sc $Dy_2ScN@C_{80}$(Ad)**						
**χ_x, cm^3 mol^{-1}**	0.009	0.009				
**χ_y, cm^3 mol^{-1}**	0.011	0.010				
**χ_z, cm^3 mol^{-1}**	0.117	0.118				
**(χ_z – 0.5{χ_x+χ_y})**	0.107	0.109				
**δ^{para}, Eq. 1**						
a	70.7	57.0	127.7	122.7	**19–33**	**82.0**
b	–45.0	103.9	58.9	53.4		**52.4**
b'	111.6	–65.8	45.8	40.2		**47.2**
c	–5.9	41.9	36.0	30.2		**31.9**
c'	49.4	–17.7	31.7	26.0		**30.0**
d	–18.5	44.4	25.9	20.1		**25.7**
d'	50.5	–29.5	21.0	15.2		**23.0**
e	14.0	6.9	20.9	15.1		**20.7**
a	70.7	57.0	127.7	122.7		**82.0**
**⟨*b*⟩**	33.3	19.1	52.3	46.8		**49.8**
**⟨*c*⟩**	21.7	12.1	33.9	28.1		**31.0**
**⟨*d*⟩**	16.0	7.5	23.5	17.6		**24.4**
e	14.0	6.9	20.9	15.1		**20.7**
**[5,6]-^{Ad}Sc $Dy_2ScN@C_{80}$(Ad)**						
**χ_x, cm^3 mol^{-1}**	0.009	0.007				
**χ_y, cm^3 mol^{-1}**	0.012	0.009				
**χ_z, cm^3 mol^{-1}**	0.117	0.120				
**(χ_z – 0.5{χ_x+χ_y})**	0.106	0.111				
**δ^{para}, Eq. 1**						
a	-21.76	118.33	96.6	63.2	**25-41**	**82.9**
a'	110.72	15.67	126.4	87.6		**59.8**
b	-6.74	54.87	48.1	23.5		**43.7**
b'	40.74	14.11	54.8	29.0		**36.8**
c	-14.05	42.11	28.1	7.0		**28.0**
c'	34.34	-0.69	33.6	11.6		**21.6**
d	6.54	15.66	22.2	2.2		**19.0**
e	0.35	18.50	18.8	-0.5		**16.8**
e'	11.47	8.30	19.8	0.2		**15.4**
**⟨*a*⟩**	44.5	67.0	111.5	75.4		**71.4**
**⟨*b*⟩**	17.0	34.5	51.5	26.2		**40.2**
**⟨*c*⟩**	10.1	20.7	30.9	9.3		**24.8**
d	6.5	15.7	22.2	2.2		**19.0**
**⟨*e*⟩**	5.9	13.4	19.3	-0.2		**16.1**

χ values are computed *ab initio* for *T* = 298 K and are given in CGS system, "δ^{para} sum" are chemical shifts obtained as direct sum of two or three components (for Dy_2ScN and Dy_3N, respectively), "δ^{para} sum*" are obtained by summation of the values, scaled using linear fit parameters from Figures S11a and S13c.

DFT computations

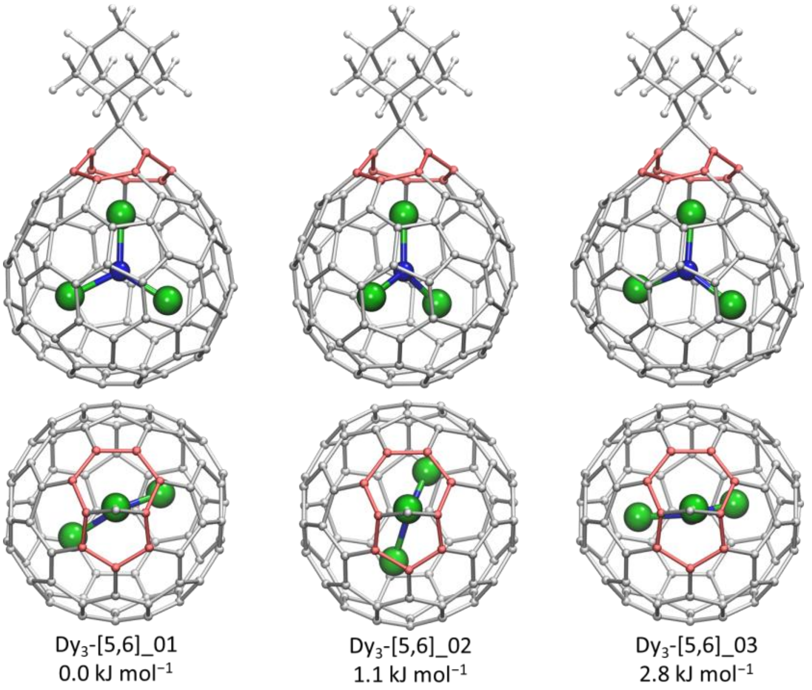

Figure S18. DFT-optimized structures of three unique conformers of [5,6]-open $Dy_3N@C_{80}$(Ad); each conformer is shown in two projections; only the bridge atom of the Ad moiety is shown in the bottom row.

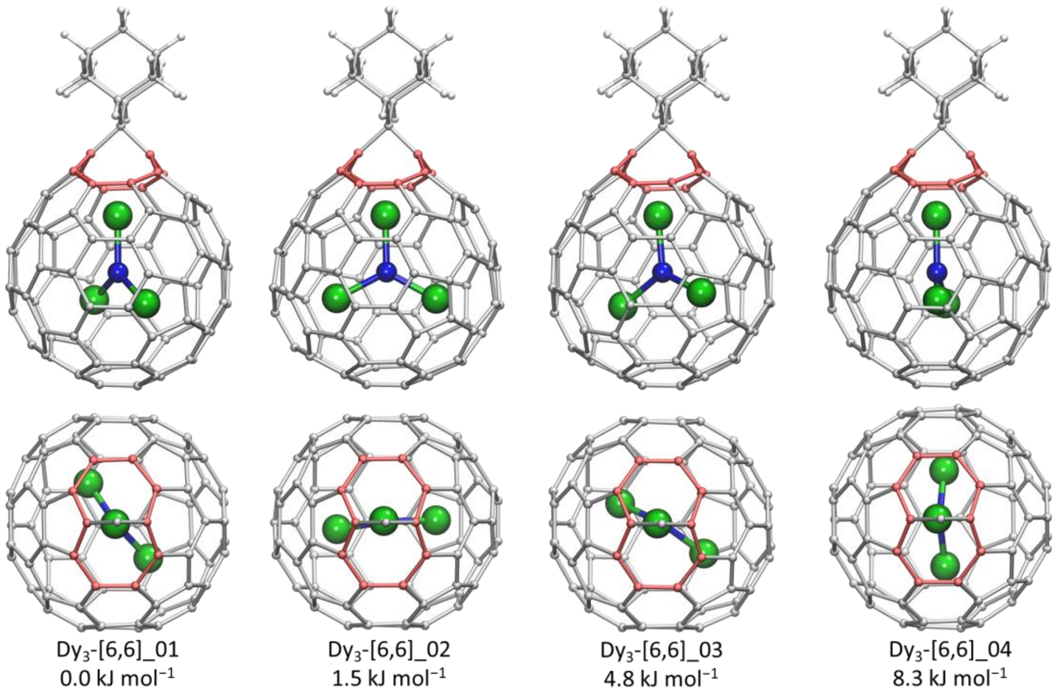

Figure S19. DFT-optimized structures of four unique conformers of [6,6]-open $Dy_3N@C_{80}$(Ad); each conformer is shown in two projections; only the bridge atom of the Ad moiety is shown in the bottom row.

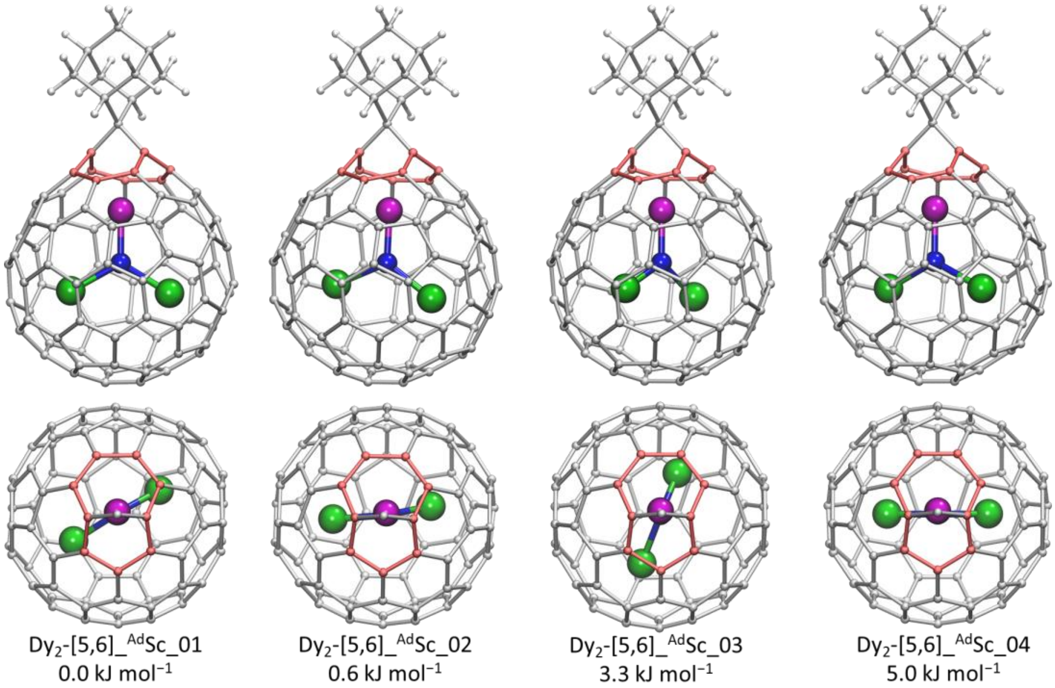

Figure S20. DFT-optimized structures of four unique conformers of [5,6]-open $Dy_2ScN@C_{80}$(Ad) with ^{Ad}Sc coordination; each conformer is shown in two projections; only the bridge atom of the Ad moiety is shown in the bottom row.

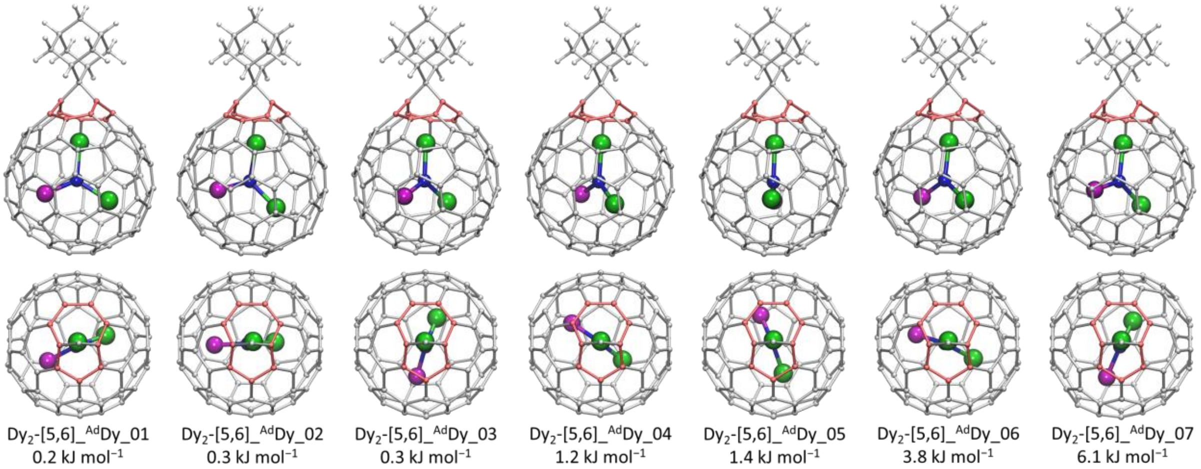

Figure S21. DFT-optimized structures of seven unique conformers of [5,6]-open $Dy_2ScN@C_{80}$(Ad) with ^{Ad}Dy coordination; each conformer is shown in two projections; only the bridge atom of the Ad moiety is shown in the bottom row.

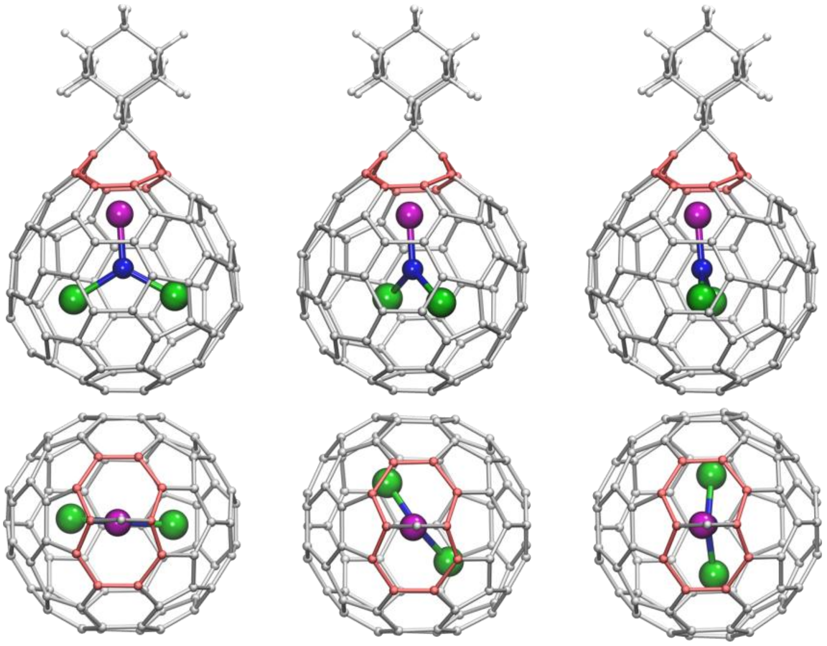

Dy$_2$-[6,6]_^{Ad}Sc_01 6.6 kJ mol^{-1} | Dy$_2$-[6,6]_^{Ad}Sc_02 8.0 kJ mol^{-1} | Dy$_2$-[6,6]_^{Ad}Sc_03 14.1 kJ mol^{-1}

Figure S22. DFT-optimized structures of three unique conformers of [6,6]-open $Dy_2ScN@C_{80}$(Ad) with ^{Ad}Sc coordination; each conformer is shown in two projections; only the bridge atom of the Ad moiety is shown in the bottom row.

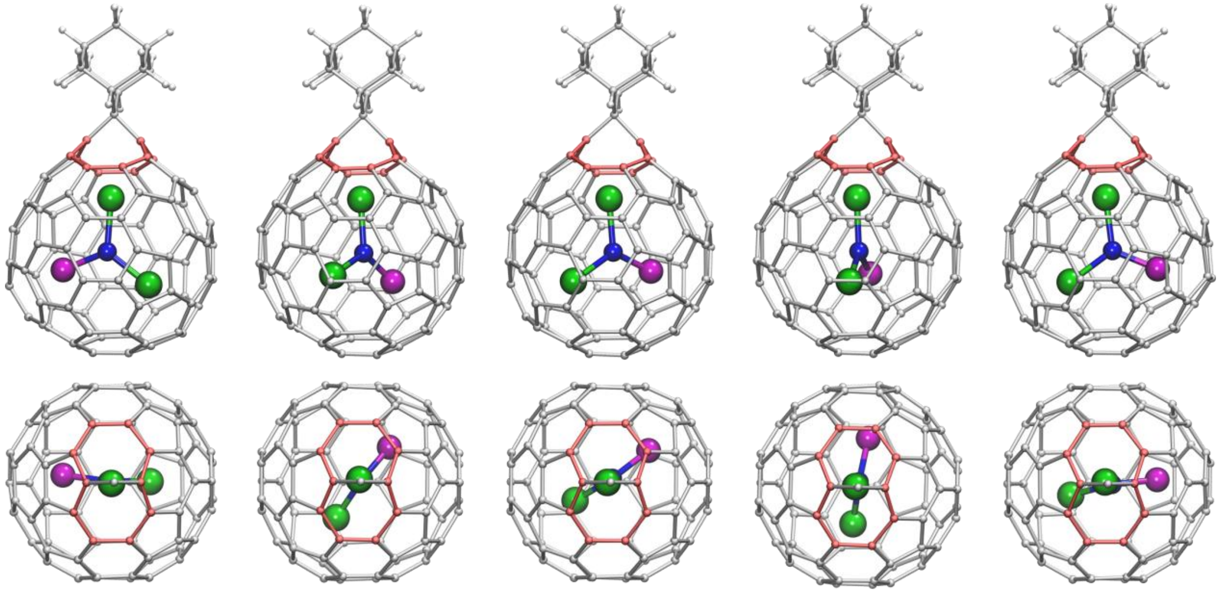

Dy$_2$-[6,6]_^{Ad}Dy_01 0.0 kJ mol^{-1} | Dy$_2$-[6,6]_^{Ad}Sc_02 0.9 kJ mol^{-1} | Dy$_2$-[6,6]_^{Ad}Sc_03 2.7 kJ mol^{-1} | Dy$_2$-[6,6]_^{Ad}Sc_04 2.9 kJ mol^{-1} | Dy$_2$-[6,6]_^{Ad}Sc_05 3.6 kJ mol^{-1}

Figure S23. DFT-optimized structures of five unique conformers of [6,6]-open $Dy_2ScN@C_{80}$(Ad) with ^{Ad}Dy coordination; each conformer is shown in two projections; only the bridge atom of the Ad moiety is shown in the bottom row.

Low-frequency vibrations

Table S6. Lowest DFT-computed vibrational frequencies (cm^{-1}) of selected $Dy_xSc_{3-x}N@C_{80}(Ad)$ conformers.[a]

	**Dy_2-I-^{Ad}Sc**		**Dy_2-II-^{Ad}Sc**		**Dy_2-II-^{Ad}Dy**		**Dy_3-I**		**Dy_3-II**	
	**56_01**	**56_02**	**66_01**	**66_02**	**66_01**	**66_02**	**56_01**	**56_02**	**66_01**	**66_02**
**1**	26.4	28.2	23.6	21.1	45.6	41.1	20.6	35.5	35.7	29.8
**2**	40.7	36.3	45.9	37.5	48.0	46.2	33.4	40.2	43.2	37.5
**3**	51.9	51.4	49.3	52.1	53.1	49.9	50.1	45.5	48.2	52.1
**4**	54.7	54.0	58.8	54.0	56.2	60.2	51.3	58.8	56.9	56.5
**5**	65.2	65.4	59.4	59.8	66.2	70.7	62.3	64.1	59.9	59.5
**6**	66.9	65.8	71.8	61.7	76.6	76.8	67.2	70.1	71.5	66.4
**7**	76.2	76.0	76.3	76.7	83.1	81.1	76.5	76.5	77.3	77.4
**8**	132.3	137.6	112.8	121.8	90.3	89.1	84.0	85.0	86.8	84.1
**9**	149.1	148.0	147.9	142.6	130.6	121.5	100.0	103.9	94.9	97.8

[a] Hessian calculations were performed at the PBE/TZ2P level for Y analogs, vibrational frequencies were then recalculated using atomic mass of Dy for Y atoms.

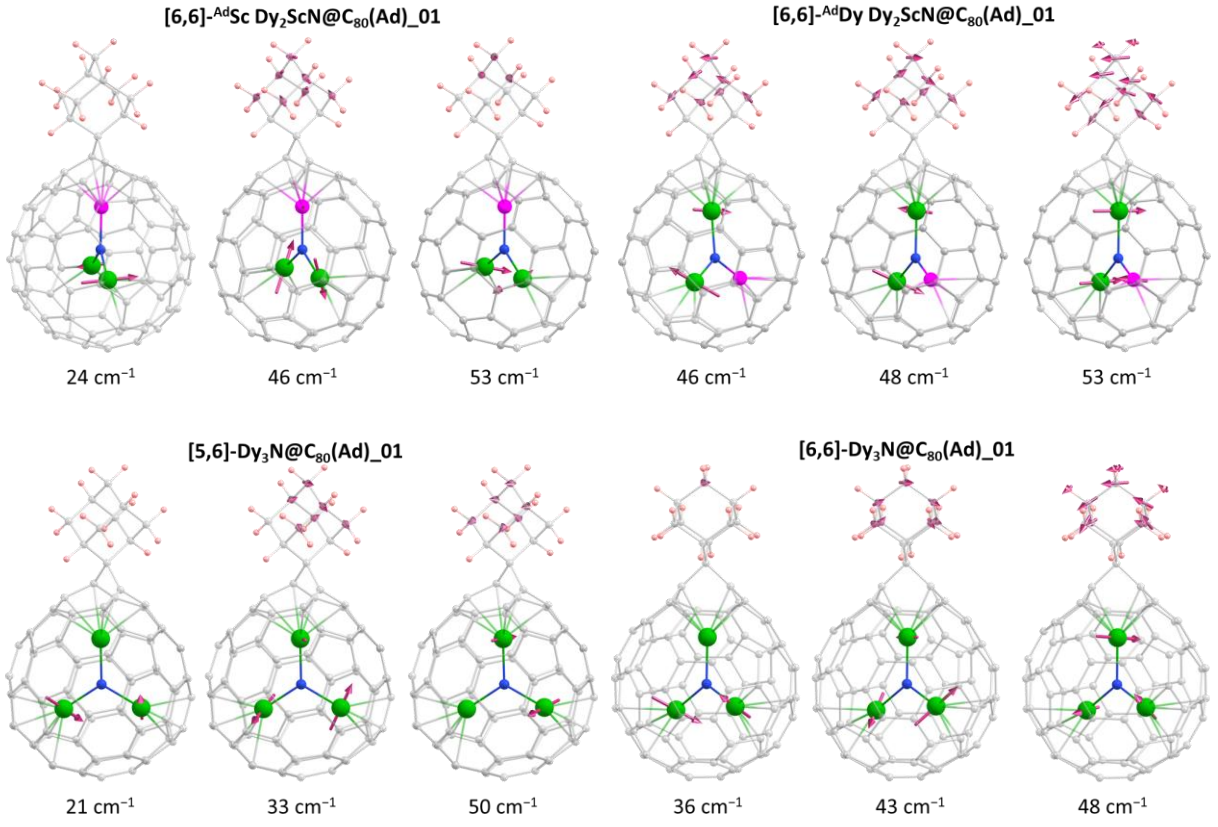

Figure S24. Vibrational displacements of the three lowest-frequency modes in representative $Dy_xSc_{3-x}N@C_{80}(Ad)$ conformers.

CASSCF calculations for ^{Ad}Sc-[5,6]-open $Dy_2ScN@C_{80}(Ad)$

Table S7a. LF splitting, pseudospin g-tensors, composition of LF states in m_J basis (%), and contribution of different B_q^k terms to ligand splitting (%) in [5,6]-open $Dy_2ScN@C_{80}$(Ad), conformer ^{Ad}Sc_01, ^{cage}**Dy1**.

	E cm^{-1}	g_x	g_y	g_z	|±15/2⟩	|±13/2⟩	|±11/2⟩	|±9/2⟩	|±7/2⟩	|±5/2⟩	|±3/2⟩	|±1/2⟩		
KD1	0	0.00006	0.00008	19.8460	**99.2**	0	0.5	0.2	0	0	0	0	$\sum B_q^2$	78.1
KD2	365	0.00178	0.00192	17.0672	0	**98.4**	0.9	0.4	0.3	0	0	0	$\sum B_q^4$	8.3
KD3	728	0.03955	0.04499	14.2805	0.5	0.8	**95.6**	2.3	0.5	0.1	0	0.1	$\sum B_q^6$	11.5
KD4	1011				0.2	0.5	1.9	**90.1**	2	3.7	0.8	0.7	$\sum B_q^8$	1.4
KD5	1167				0	0.2	0.8	2.9	**65.2**	4.2	**15.2**	**11.6**		
KD6	1254				0	0	0.1	2.7	**22.1**	**38.9**	**9.8**	**26.4**	B_0^2	47.0
KD7	1322				0	0.1	0.2	1	7.1	**39.6**	**39.3**	**12.7**	B_0^4	4.9
KD8	1429				0	0	0	0.4	2.8	**13.4**	**34.9**	**48.3**	B_0^6	3.1

Table S7b. LF splitting, pseudospin g-tensors, composition of LF states in m_J basis (%), and contribution of different B_q^k terms to ligand splitting (%) in [5,6]-open $Dy_2ScN@C_{80}$(Ad), conformer ^{Ad}Sc_01, ^{cage}**Dy2**.

	E cm^{-1}	g_x	g_y	g_z	|±15/2⟩	|±13/2⟩	|±11/2⟩	|±9/2⟩	|±7/2⟩	|±5/2⟩	|±3/2⟩	|±1/2⟩		
KD1	0	0.00028	0.00037	19.8900	**99.8**	0	0	0	0	0	0	0	$\sum B_q^2$	80.1
KD2	527	0.01951	0.02368	17.0009	0	**97.1**	1.7	0.9	0.1	0.1	0	0	$\sum B_q^4$	8.1
KD3	812	0.18937	0.19728	14.2680	0.1	2.3	**89**	5.1	2.8	0.1	0.2	0.3	$\sum B_q^6$	10.5
KD4	996				0	0.5	6.3	**86.9**	1.6	4	0.5	0.2	$\sum B_q^8$	1.0
KD5	1137				0	0	2.6	2.6	**88**	2.3	4.3	0.2		
KD6	1273				0	0	0	3.5	1.9	**79.5**	5.5	**9.5**	B_0^2	47.0
KD7	1384				0	0	0.1	0.8	4.5	8	**65.9**	**20.8**	B_0^4	4.7
KD8	1511				0	0	0.1	0.2	1.1	5.9	**23.5**	**69.2**	B_0^6	4.3

CASSCF calculations for ^{Ad}Sc-[6,6]-open $Dy_2ScN@C_{80}$(Ad)

Table S8a. LF splitting, pseudospin g-tensors, composition of LF states in m_J basis (%), and contribution of different B_q^k terms to LF splitting (%) in [6,6]-open $Dy_2ScN@C_{80}$(Ad), conformer ^{Ad}Sc_01, ^{cage}**Dy1**.

	E cm^{-1}	g_x	g_y	g_z	|±15/2⟩	|±13/2⟩	|±11/2⟩	|±9/2⟩	|±7/2⟩	|±5/2⟩	|±3/2⟩	|±1/2⟩		
KD1	0	0.00038	0.00044	19.8590	**99.5**	0	0.3	0.1	0.1	0	0	0	$\sum B_q^2$	77.2
KD2	396	0.00702	0.00709	17.1173	0	**96.6**	2.7	0.3	0.1	0.2	0.1	0	$\sum B_q^4$	8.6
KD3	742	0.07003	0.08284	14.3033	0.2	2.9	**88.9**	7.2	0.3	0.1	0.2	0.2	$\sum B_q^6$	12.2
KD4	1017				0.2	0.1	7.4	**85.2**	6.1	0.2	0.3	0.4	$\sum B_q^8$	1.4
KD5	1181				0.1	0.2	0.1	6	**88.1**	1.7	1.6	2.2		
KD6	1242				0	0.1	0	0.4	1.9	**55.3**	**23**	**19.3**	B_0^2	44.3
KD7	1310				0	0.1	0.2	0.4	2.9	**32.8**	**38**	**25.5**	B_0^4	4.8
KD8	1428				0	0.1	0.3	0.6	0.6	9.5	**36.6**	**52.3**	B_0^6	1.2

Table S8b. LF splitting, pseudospin g-tensors, composition of LF states in m_J basis (%), and contribution of different B_q^k terms to LF splitting (%) in [6,6]-open $Dy_2ScN@C_{80}$(Ad), conformer ^{Ad}Sc_01, ^{cage}**Dy2**.

	E cm^{-1}	g_x	g_y	g_z	|±15/2⟩	|±13/2⟩	|±11/2⟩	|±9/2⟩	|±7/2⟩	|±5/2⟩	|±3/2⟩	|±1/2⟩		
KD1	0	0.00006	0.00007	19.8824	**99.9**	0	0	0	0	0	0	0	$\sum B_q^2$	78.7
KD2	439	0.00091	0.00093	17.1720	0	**95.6**	3.9	0.1	0.1	0.1	0.1	0	$\sum B_q^4$	8.1
KD3	758	0.05973	0.06902	14.2986	0.1	3.9	**84.4**	11.2	0	0	0.1	0.4	$\sum B_q^6$	11.2
KD4	1004				0.1	0.1	9.5	**75.8**	**12.8**	0.7	0.6	0.3	$\sum B_q^8$	1.5
KD5	1169				0	0.1	1.6	**10.4**	**81.3**	3.3	2.3	0.9		
KD6	1269				0	0.1	0	1.6	4	**85.1**	8	1.1	B_0^2	48.9
KD7	1348				0	0.1	0	0.5	0.7	4.5	**61**	**33.1**	B_0^4	4.9
KD8	1430				0	0.1	0.3	0.2	1	6.2	**28**	**64.2**	B_0^6	1.0

CASSCF calculations for ^{Ad}Dy-[6,6]-open $Dy_2ScN@C_{80}$(Ad)

Table S9a. LF splitting, pseudospin g-tensors, composition of LF states in m_J basis (%), and contribution of different B_q^k terms to LF splitting (%) in [6,6]-open $Dy_2ScN@C_{80}$(Ad), conformer ^{Ad}Dy_01, ^{Ad}**Dy1**.

	E cm^{-1}	g_x	g_y	g_z	$\|\pm15/2\rangle$	$\|\pm13/2\rangle$	$\|\pm11/2\rangle$	$\|\pm9/2\rangle$	$\|\pm7/2\rangle$	$\|\pm5/2\rangle$	$\|\pm3/2\rangle$	$\|\pm1/2\rangle$		
KD1	0	0.00298	0.00378	19.7669	**98**	0	1.6	0	0.2	0	0	0	$\sum B_q^2$	77.8
KD2	329				0	**97.1**	0.9	1.2	0	0.7	0.1	0	$\sum B_q^4$	5.8
KD3	597				1.7	1	**93**	3	0.2	0	0.7	0.2	$\sum B_q^6$	14.7
KD4	784				0	1.4	3.1	**91.2**	3	0.4	0.5	0.4	$\sum B_q^8$	1.2
KD5	904				0.1	0	0.5	2.9	**81.5**	8.2	6.2	0.4		
KD6	953				0	0.1	0.1	0.4	1.7	**35.3**	**21.8**	**40.6**	B_0^2	42.4
KD7	1039				0	0.3	0.2	0.8	**12.3**	**44.1**	**36.6**	5.6	B_0^4	3.0
KD8	1252				0	0.1	0.3	0.4	1	**11.2**	**34.2**	**52.7**	B_0^6	0.3

Table S9b. LF splitting, pseudospin g-tensors, composition of LF states in m_J basis (%), and contribution of different B_q^k terms to LF splitting (%) in [6,6]-open $Dy_2ScN@C_{80}$(Ad), conformer ^{Ad}Dy_01, ^{cage}**Dy2**.

	E cm^{-1}	g_x	g_y	g_z	$\|\pm15/2\rangle$	$\|\pm13/2\rangle$	$\|\pm11/2\rangle$	$\|\pm9/2\rangle$	$\|\pm7/2\rangle$	$\|\pm5/2\rangle$	$\|\pm3/2\rangle$	$\|\pm1/2\rangle$		
KD1	0	0.00002	0.00002	19.8875	**99.9**	0	0	0	0	0	0	0	$\sum B_q^2$	79.2
KD2	444				0	**95.1**	4.5	0.2	0.1	0.1	0.1	0	$\sum B_q^4$	7.9
KD3	756				0.1	4.5	**82.5**	**12.6**	0	0	0	0.3	$\sum B_q^6$	10.8
KD4	998				0	0.1	**11.2**	**73.5**	**13.9**	0.4	0.6	0.2	$\sum B_q^8$	1.5
KD5	1159				0	0.1	1.5	**11.9**	**80.3**	4.2	1.3	0.5		
KD6	1261				0	0.1	0	1.1	4.9	**86**	6.7	1.2	B_0^2	48.1
KD7	1342				0	0.1	0	0.4	0.3	4.6	**65.4**	**29.4**	B_0^4	4.7
KD8	1425				0	0	0.2	0	0.6	4.7	**26**	**68.4**	B_0^6	1.5

CASSCF calculations for [5,6]-open $Dy_3N@C_{80}$(Ad)

Table S10a. LF splitting, pseudospin g-tensors, composition of LF states in m_J basis (%), and contribution of different B_q^k terms to LF splitting (%) in [5,6]-open $Dy_3N@C_{80}$(Ad), conformer _01, ^{Ad}**Dy1**.

	E cm^{-1}	g_x	g_y	g_z	|±15/2⟩	|±13/2⟩	|±11/2⟩	|±9/2⟩	|±7/2⟩	|±5/2⟩	|±3/2⟩	|±1/2⟩		
KD1	0	0.00084	0.00124	19.6408	**95.6**	0	4.2	0	0.1	0	0	0	$\sum B_q^2$	79.7
KD2	326				0	**96**	0	3.7	0	0.2	0	0	$\sum B_q^4$	5.3
KD3	637				4.3	0	**93.1**	0.1	2.1	0	0.3	0.1	$\sum B_q^6$	13.1
KD4	831				0	3.8	0.1	**89.2**	0.5	4.6	0.4	1.4	$\sum B_q^8$	1.4
KD5	922				0	0.1	1.4	3.2	**37**	8.9	**27.5**	**21.7**		
KD6	991				0	0	1	2.6	**46.9**	**28.1**	1.8	**19.6**	B_0^2	44.5
KD7	1118				0	0	0.1	1	11.9	**46.1**	**36.1**	4.9	B_0^4	2.5
KD8	1368				0	0	0	0	1.6	**12**	**33.9**	**52.4**	B_0^6	0.5

Table S10b. LF splitting, pseudospin g-tensors, composition of LF states in m_J basis (%), and contribution of different B_q^k terms to LF splitting (%) in [5,6]-open $Dy_3N@C_{80}$(Ad), conformer _01, ^{cage}**Dy2**.

	E cm^{-1}	g_x	g_y	g_z	|±15/2⟩	|±13/2⟩	|±11/2⟩	|±9/2⟩	|±7/2⟩	|±5/2⟩	|±3/2⟩	|±1/2⟩		
KD1	0	0.00008	0.00012	19.8331	**99.1**	0	0.6	0.3	0	0	0	0	$\sum B_q^2$	76.9
KD2	382				0	**98.7**	0.3	0.5	0.4	0	0	0	$\sum B_q^4$	9.3
KD3	770				0.5	0.3	**96.9**	1.4	0.6	0.1	0.1	0.1	$\sum B_q^6$	11.9
KD4	1070				0.4	0.4	1.4	**90**	1.9	3	1.7	1.1	$\sum B_q^8$	1.3
KD5	1223				0	0.3	0.3	5.7	**52.6**	7.4	**14.5**	**19.2**		
KD6	1309				0	0.1	0.2	1.1	**35.3**	**29.2**	8.3	**25.7**	B_0^2	44.8
KD7	1368				0	0	0.1	0.7	5.2	**43.8**	**43.1**	7.1	B_0^4	5.3
KD8	1495				0	0	0.1	0.4	4	**16.3**	**32.3**	**46.9**	B_0^6	3.5

Table S10c. LF splitting, pseudospin g-tensors, composition of LF states in m_J basis (%), and contribution of different B_q^k terms to LF splitting (%) in [5,6]-open $Dy_3N@C_{80}$(Ad), conformer _01, ^{cage}**Dy3**.

	E cm^{-1}	g_x	g_y	g_z	|±15/2⟩	|±13/2⟩	|±11/2⟩	|±9/2⟩	|±7/2⟩	|±5/2⟩	|±3/2⟩	|±1/2⟩		
KD1	0	0.00028	0.00039	19.8899	**99.9**	0	0	0	0	0	0	0	$\sum B_q^2$	78.7
KD2	543				0	**95.9**	2.9	0.8	0.1	0.1	0	0	$\sum B_q^4$	8.6
KD3	842				0.1	3.5	**84.1**	**9.5**	2.3	0.1	0.1	0.2	$\sum B_q^6$	10.9
KD4	1042				0	0.5	**9.9**	**80**	4.9	3.9	0.7	0.1	$\sum B_q^8$	1.2
KD5	1179				0	0	2.7	4.6	**83.8**	3.6	4.8	0.5		
KD6	1313				0	0	0.1	4	3.2	**76.6**	5.6	**10.5**	B_0^2	46.1
KD7	1424				0	0	0.1	0.8	4.6	9.3	**63.4**	**21.7**	B_0^4	5.1
KD8	1561				0	0	0.1	0.2	1.2	6.2	**25.3**	**66.9**	B_0^6	4.2

CASSCF calculations for [6,6]-open $Dy_3N@C_{80}(Ad)$

Table S11a. LF splitting, pseudospin g-tensors, composition of LF states in m_J basis (%), and contribution of different B_q^k terms to LF splitting (%) in [6,6]-open $Dy_3N@C_{80}$(Ad), conformer _01, ^{Ad}**Dy1**.

	E cm^{-1}	g_x	g_y	g_z	|±15/2⟩	|±13/2⟩	|±11/2⟩	|±9/2⟩	|±7/2⟩	|±5/2⟩	|±3/2⟩	|±1/2⟩		
KD1	0	0.00177	0.00218	19.8153	**98.9**	0	0.8	0	0.2	0	0	0	$\sum B_q^2$	77.6
KD2	319				0	**97.7**	1.2	0.2	0.1	0.7	0	0	$\sum B_q^4$	6.4
KD3	585				0.7	1.1	**93**	4	0.4	0	0.8	0.1	$\sum B_q^6$	14.4
KD4	789				0.1	0.3	3.6	**91.7**	2	1.6	0.6	0.1	$\sum B_q^8$	1.1
KD5	912				0.2	0.1	0.1	2	**71.8**	**11.3**	8.6	5.8		
KD6	1001				0.1	0.4	0.6	0.8	**22.7**	**40.3**	**14.7**	**20.4**	B_0^2	43.3
KD7	1065				0	0.2	0.2	0.5	1.8	**35.9**	**39.9**	**21.4**	B_0^4	3.2
KD8	1197				0	0.2	0.5	0.8	0.8	**10.2**	**35.2**	**52.3**	B_0^6	0.4

Table S11b. LF splitting, pseudospin g-tensors, composition of LF states in m_J basis (%), and contribution of different B_q^k terms to LF splitting (%) in [6,6]-open $Dy_3N@C_{80}$(Ad), conformer _01, ^{cage}**Dy2**.

	E cm^{-1}	g_x	g_y	g_z	|±15/2⟩	|±13/2⟩	|±11/2⟩	|±9/2⟩	|±7/2⟩	|±5/2⟩	|±3/2⟩	|±1/2⟩		
KD1	0	0.00003	0.00003	19.8768	**99.7**	0	0.2	0.1	0	0	0	0	$\sum B_q^2$	77.6
KD2	437				0	**95.9**	3.7	0.2	0	0.1	0.1	0	$\sum B_q^4$	8.8
KD3	780				0.3	3.7	**85.7**	**9.9**	0.1	0.1	0.1	0.2	$\sum B_q^6$	11.1
KD4	1039				0	0.1	8	**75.9**	**12.7**	2.3	0.7	0.3	$\sum B_q^8$	1.7
KD5	1211				0	0.1	2.1	9.3	**74.4**	5.8	7	1.2		
KD6	1307				0	0.1	0.3	3.9	9	**67.5**	**10.8**	8.5	B_0^2	45.4
KD7	1380				0	0	0	0.8	2.1	**14.5**	**45.5**	**37.1**	B_0^4	4.9
KD8	1471				0	0.1	0.2	0.1	1.5	9.6	**35.8**	**52.8**	B_0^6	0.1

Table S11c. LF splitting, pseudospin g-tensors, composition of LF states in m_J basis (%), and contribution of different B_q^k terms to LF splitting (%) in [6,6]-open $Dy_3N@C_{80}$(Ad), conformer _01, ^{cage}**Dy3**.

	E cm^{-1}	g_x	g_y	g_z	|±15/2⟩	|±13/2⟩	|±11/2⟩	|±9/2⟩	|±7/2⟩	|±5/2⟩	|±3/2⟩	|±1/2⟩		
KD1	0	0.00007	0.00008	19.8556	**99.4**	0	0.5	0.1	0	0	0	0	$\sum B_q^2$	77.5
KD2	424				0	**97.3**	2	0.3	0.1	0.1	0.1	0	$\sum B_q^4$	8.9
KD3	785				0.5	2.2	**91.5**	5.1	0.2	0	0.1	0.2	$\sum B_q^6$	11.4
KD4	1053				0	0.2	4.6	**86.3**	5.6	2.6	0.2	0.5	$\sum B_q^8$	1.6
KD5	1221				0	0	0.8	4	**78.2**	2.6	**10.3**	4		
KD6	1305				0	0.1	0.2	3.2	**10.5**	**55.9**	**15.1**	**14.9**	B_0^2	46.4
KD7	1400				0	0.1	0	0.6	4.2	**31.3**	**35.8**	**27.9**	B_0^4	5.1
KD8	1474				0	0	0.2	0.4	1	7.3	**38.5**	**52.5**	B_0^6	1.2

DFT-optimized Cartesian coordinates of the conformers used in CASSCF calculations

[5,6]-open $Dy_2ScN@C_{80}$(Ad), conformer ^{Ad}Sc_01

Dy	0.00000000	0.00000000	0.00000000
Dy	-1.84685489	0.08520791	-3.20048753
Sc	1.70167258	-0.15481109	-3.08065739
H	5.25527829	-2.46916220	-3.70495812
H	4.43293536	1.32651609	-5.63775787
H	6.81829344	-0.68848393	-2.89292504
H	7.66552703	-1.97136844	-3.76831592
H	6.33920265	1.53630963	-4.02576951
H	6.83582848	1.80818452	-5.70080355
H	4.87754186	-2.77453242	-6.16060948
H	6.52865286	-3.19297628	-5.68313021
H	4.39655057	-0.55121197	-7.29731175
H	5.70764655	0.59589641	-7.60045312
H	8.45537265	0.29989067	-4.50507753
H	6.52025291	-1.77918341	-7.76314661
H	8.45191147	-1.56740973	-6.17858037
H	8.11512638	-0.01734678	-6.96744854
C	4.12884869	-0.00572228	-3.17046296
C	3.15967981	-1.04896540	-4.80633161
C	2.65633897	-2.16245493	-3.99680818
C	3.07813105	-2.19068383	-2.59217085
C	3.76204904	-0.97435916	-2.13503824
C	3.53844773	-0.51394324	-0.77444046
C	3.46988330	0.88324093	-0.53613647
C	3.38962077	1.78620035	-1.65929375
C	3.58010715	1.33944249	-3.00745413
C	2.68290593	1.92946302	-3.99816356
C	2.01760756	1.21516738	-5.11554333
C	2.14159259	-0.20508063	-5.43069509
C	0.92203327	-0.86005539	-5.80498554
C	0.60040717	-2.19625669	-5.36845199
C	1.40679120	-2.81082440	-4.36338178
C	0.76263048	-3.66500284	-3.44145533
C	1.22906742	-3.77081239	-2.08691420
C	2.33196728	-2.99924423	-1.63117918
C	2.29761249	-2.58902476	-0.25788833
C	2.88859383	-1.35796386	0.16298412
C	2.13756212	-0.83471841	1.28817477
C	1.99195564	0.58253163	1.49191444
C	2.67034677	1.42264053	0.53996370
C	2.10200055	2.66162264	0.06324114
C	2.53314609	2.86905635	-1.28859390
C	1.69735218	3.49145874	-2.25693611
C	1.79516334	3.01591835	-3.60543998
C	0.63929588	3.00242091	-4.44607214
C	0.75009345	1.90047017	-5.35454562
C	-0.41864591	1.21839177	-5.81479962
C	-0.30939641	-0.17317335	-6.04665599
C	-1.41114286	-1.07390738	-5.79259931
C	-0.82820580	-2.32844501	-5.35825499
C	-1.48087820	-3.12250649	-4.37287761
C	-0.66722067	-3.83525042	-3.44695730
C	-1.08383709	-4.04396438	-2.08797905
C	0.08565216	-4.02841605	-1.24892355
C	0.01798956	-3.52094143	0.07935059
C	1.16438138	-2.84053470	0.58908437
C	1.05675094	-1.76717816	1.56577412
C	-0.22436422	-1.31697037	2.07436314
C	-0.37130096	0.11615934	2.36991335
C	0.73501530	1.05360097	2.06206618
C	0.14821948	2.24930909	1.47258213
C	0.80997755	3.07871298	0.48890528
C	0.00706137	3.79307582	-0.44486124
C	0.44816957	4.00435665	-1.80261185
C	-0.70931058	3.97951533	-2.65172655
C	-0.63488895	3.43869410	-3.96221402
C	-1.80014174	2.76315384	-4.46045149
C	-1.71937342	1.68850053	-5.40357233
C	-2.89434939	0.82945550	-5.25165411
C	-2.71995209	-0.61589729	-5.39040029
C	-3.41057661	-1.46545279	-4.44045737
C	-2.77094998	-2.67213575	-3.93343379
C	-3.17104500	-2.87161698	-2.57248483
C	-2.33031424	-3.54136507	-1.61988706
C	-2.41388007	-3.12169436	-0.25616313
C	-1.25047575	-3.10763721	0.58231652
C	-1.36105662	-1.99546344	1.50627640
C	-2.61452544	-1.32794631	1.20551386
C	-2.73400990	0.08098147	1.35966417
C	-1.59895461	0.77638724	1.92773696
C	-1.27558986	2.08554396	1.41678380
C	-2.08673195	2.76963666	0.43983490
C	-1.42641203	3.64673402	-0.46459015
C	-1.87322175	3.77533391	-1.82788979
C	-2.99400199	3.03445297	-2.30022850
C	-2.98104407	2.57480128	-3.66672299
C	-3.68885620	1.39405464	-4.14157987
C	-4.31864814	0.52997195	-3.14786124
C	-4.19645091	-0.90186176	-3.33910121
C	-4.02193773	-1.77854325	-2.19508882
C	-4.03308065	-1.31118990	-0.83957877
C	-3.26154266	-2.02557406	0.12992746
C	-4.21298765	0.08414826	-0.64871826
C	-4.30109665	0.98259048	-1.78103138
C	-3.71287806	2.23957915	-1.36569550
C	-3.27053228	2.11115825	-0.00609858
C	-3.58078199	0.77855734	0.44157217
C	4.56545166	-0.55470440	-4.51655877
C	5.61430148	-1.70393360	-4.40573029
C	5.13061698	0.48981866	-5.51860776
C	6.95129291	-1.14112031	-3.88896610
C	6.47444678	1.03912919	-4.99957537
C	5.82426784	-2.35201146	-5.78682564
C	5.34880219	-0.17037287	-6.89480948
C	7.49919690	-0.09992271	-4.87821736
C	6.37153543	-1.31151994	-6.77691425
C	7.71006176	-0.75559790	-6.25586097
N	0.00362052	-0.00538178	-2.13520214

[6,6]-open $Dy_2ScN@C_{80}$(Ad), conformer ^{Ad}Sc_01

Dy	0.00000000	0.00000000	0.00000000
Dy	-1.84201723	0.06007126	-3.22154057
Sc	1.72905508	-0.00539658	-3.05870149
H	4.67982997	2.27686717	-4.82881680
H	4.60581283	-2.04370530	-5.15302001
H	5.79472929	2.41909941	-7.02688639
H	4.25335840	1.56965993	-7.19555356
H	7.14543913	2.23606342	-4.89770282
H	6.57267004	1.25269335	-3.54377910
H	4.21013975	-0.97763046	-7.38699457
H	5.72131427	-1.89494460	-7.35069140
H	7.07153931	-2.07732876	-5.22245368
H	6.52953290	-1.28885257	-3.73523117
H	6.04981492	0.35063285	-8.43304921
H	8.36256211	0.03797608	-4.79534829
H	7.99083200	1.10087797	-7.03529175
H	7.96086538	-0.66531561	-7.16800637
C	4.38740661	0.10908690	-4.83422547
C	4.07811420	0.00295776	-3.35939700
C	3.72765281	-1.22954052	-2.64741435
C	3.59697168	-0.86375060	-1.25075310
C	3.61811570	0.56044599	-1.14412133
C	3.75863720	1.12307141	-2.47302297
C	2.94956648	2.31010714	-2.77850890
C	2.22844834	2.42519830	-4.03816739
C	2.14094177	1.37622477	-5.07425431
C	2.91637568	0.16096068	-5.18970710
C	2.10049098	-1.03337696	-5.26192189
C	2.14634944	-2.22312076	-4.38683408
C	2.87113767	-2.32562147	-3.12495487
C	2.26695889	-3.15624297	-2.10983002
C	2.35647340	-2.86001045	-0.69587047
C	2.96443722	-1.66651484	-0.25806191
C	2.42774919	-0.99706665	0.89561074
C	2.46534504	0.43216112	1.01931675
C	3.01953161	1.23494582	-0.03924209
C	2.45573374	2.49988544	-0.29781527
C	2.37952966	3.00707448	-1.64951861
C	1.15135378	3.74883528	-1.73784861
C	0.41530962	3.81241373	-2.95161087
C	0.97877859	3.16369557	-4.09151217
C	0.12108048	2.56803514	-5.07437280
C	0.80387841	1.44178108	-5.62036875
C	0.10281505	0.27301393	-6.02052482
C	0.76149362	-0.96702097	-5.80459044
C	0.03505002	-2.13414684	-5.42646784
C	0.86760491	-2.89802975	-4.54219585
C	0.27871859	-3.68928656	-3.51236420
C	1.01209473	-3.83110914	-2.30476873
C	0.33651822	-3.94104158	-1.03828654
C	1.16767875	-3.33392058	-0.03554881
C	0.59602192	-2.63605112	1.07818538
C	1.28708342	-1.48677091	1.62668011
C	0.61334829	-0.35082734	2.27561331
C	1.36079592	0.85769283	1.85575358
C	0.69662683	2.09255216	1.46686608
C	1.28000305	2.90801508	0.43350091
C	0.47630330	3.68983717	-0.46797149
C	-0.94630358	3.68253563	-0.38709599
C	-1.68217104	3.83462688	-1.60102034
C	-1.00839210	3.90987210	-2.86647834
C	-1.85164679	3.28453845	-3.84727078
C	-1.30485935	2.57609915	-4.94561262
C	-2.05142811	1.43899435	-5.44120090
C	-1.33456206	0.29229185	-5.93219355
C	-2.09919414	-0.88881807	-5.62346320
C	-1.39195783	-2.11013562	-5.29735235
C	-1.96545851	-2.95163362	-4.31293573
C	-1.14907303	-3.74684318	-3.43795735
C	-1.82229855	-3.83443641	-2.17379632
C	-1.08539124	-3.89467507	-0.95123164
C	-1.65562712	-3.26735672	0.20229301
C	-0.82473031	-2.63901181	1.19180891
C	-1.52902575	-1.48774371	1.73481213
C	-0.85439155	-0.32725187	2.27096573
C	-1.49372996	0.93240520	1.94616273
C	-0.73348095	2.11940199	1.56881049
C	-1.53464529	2.90365844	0.65962260
C	-2.78048248	2.22248628	0.46167252
C	-3.47679733	2.30748741	-0.78727613
C	-2.93274378	3.14937414	-1.79729181
C	-3.05227377	2.80791326	-3.19540758
C	-3.77808351	1.65520499	-3.66031222
C	-3.32286144	0.99330099	-4.88009695
C	-3.35885774	-0.48656482	-4.99916380
C	-3.84456507	-1.30571643	-3.88834378
C	-3.15199442	-2.53421260	-3.59443359
C	-3.04683835	-3.08411765	-2.26177165
C	-3.56547074	-2.38617005	-1.13482172
C	-2.87821158	-2.51946670	0.11378588
C	-2.79771241	-1.41977204	1.03524878
C	-3.40738732	-0.16883313	0.73576969
C	-2.74803508	1.00173537	1.21964994
C	-4.20532520	1.15903639	-1.22510024
C	-4.34933892	0.83055306	-2.62687462
C	-4.37934336	-0.62155056	-2.73713308
C	-4.24806766	-1.15827240	-1.39833574
C	-4.16806636	-0.06329997	-0.47425353
C	5.18368215	1.38535707	-5.22577253
C	5.14086292	-1.12077837	-5.41383043
C	5.26306490	1.49155306	-6.76128852
C	6.60811118	1.30867528	-4.64329594
C	5.22064307	-0.99998203	-6.94833082
C	6.56586943	-1.18050966	-4.83074488
C	5.99832293	0.26895150	-7.33566957
C	7.34598249	0.08721362	-5.21666990
C	7.42170712	0.20084235	-6.75074647
N	0.00387801	0.00312953	-2.13709610

[6,6]-open $Dy_2ScN@C_{80}(Ad)$, conformer $^{Ad}Dy\_01$

Dy	0.00000000	0.00000000	0.00000000	C	3.95138289	-0.59525876	-2.97149005
Dy	-1.77103664	-0.12786900	-3.34551817	C	3.31798845	-1.86229069	-3.31746140
Sc	1.75054318	0.02476076	-3.00329942	C	2.84900271	-2.72231239	-2.26227919
H	0.45798815	-2.34395199	3.55659091	C	1.70206231	-3.57364879	-2.42249217
H	-0.10931305	1.94747823	3.76532441	C	0.95453390	-3.59174439	-3.63633329
H	-0.67302277	-2.59592767	5.73538875	C	-0.44884611	-3.83725406	-3.55013093
H	-1.75407885	-1.78704694	4.59370109	C	-1.09818609	-3.96210504	-2.27545211
H	1.82927648	-2.25895300	5.60525848	C	-2.41898689	-3.41124368	-2.39760682
H	2.53808491	-1.20696770	4.37229562	C	-3.03928787	-2.76101072	-1.29985395
H	-2.08882082	0.74087904	4.71740942	C	-3.91328521	-1.66926833	-1.59728221
H	-1.23846807	1.68855016	5.94477133	C	-4.01264612	-0.55017043	-0.70392310
H	1.26235685	2.02516304	5.81419155	C	-4.22046039	0.63264421	-1.49058841
H	2.20436677	1.31680621	4.49563181	C	-3.65846340	1.88277560	-1.08465498
H	-1.79170186	-0.61091932	6.79830158	C	-3.24312518	2.76967705	-2.11072114
H	2.48555537	-0.03472311	6.57838052	C	-2.11208431	3.63389377	-1.92331559
H	0.51461948	-1.23737751	7.55459364	C	-1.46777608	3.79907673	-3.19620665
H	0.28287852	0.51662095	7.64051443	C	-0.04999043	3.94342825	-3.28853154
C	0.15294588	-0.18624588	3.36138441	C	0.60080435	3.37664099	-4.43006993
C	1.16046153	0.00264809	2.25323022	C	1.92005373	2.82598286	-4.32278000
C	1.47485531	1.27249224	1.58896405	C	2.02427838	1.69038390	-5.21962344
C	2.56534859	0.98791412	0.67667046	C	2.90144612	0.57420850	-4.97959519
C	2.75128419	-0.42728520	0.60416124	C	2.36145268	-0.71291575	-5.35203349
C	1.77606232	-1.06711174	1.46625139	C	2.56822891	-1.90386627	-4.54186419
C	1.14066790	-2.28949944	0.96164423	C	1.41874959	-2.76231397	-4.70281233
C	-0.29321007	-2.49902688	1.09676852	C	0.50244983	-2.12096578	-5.60389586
C	-1.25770792	-1.51387937	1.64194831	C	-0.91077328	-2.29804858	-5.45941431
C	-0.98458915	-0.28686445	2.36691812	C	-1.36412364	-3.19166714	-4.45316590
C	-1.58566632	0.89170561	1.76844946	C	-2.60000799	-2.92774867	-3.74850048
C	-0.91216045	2.13852950	1.32332868	C	-3.46856205	-1.82506777	-4.07473624
C	0.53015823	2.32572839	1.18631572	C	-4.12441309	-1.19852863	-2.95210200
C	0.95992503	3.19599611	0.11157543	C	-4.31830023	0.24840028	-2.88468082
C	2.18499157	2.98664810	-0.63066993	C	-3.86344708	1.12490219	-3.93821139
C	2.96027767	1.83326897	-0.40014096	C	-3.30793462	2.38020229	-3.50214115
C	3.62847077	1.21958193	-1.51335433	C	-2.19446931	3.02463314	-4.16615625
C	3.85557135	-0.19598885	-1.58034161	C	-1.53457350	2.37692209	-5.24569726
C	3.35197772	-1.04741663	-0.53180607	C	-0.12950101	2.59419422	-5.39299898
C	2.89656893	-2.33568164	-0.87023331	C	0.73889993	1.55430698	-5.87352058
C	1.77848725	-2.92614515	-0.17052864	C	0.22972249	0.27444424	-6.21787235
C	1.05122018	-3.69508649	-1.14445798	C	1.06549280	-0.85437279	-5.98615440
C	-0.35909659	-3.84641057	-1.05559977	C	-1.77919177	-1.19192803	-5.79392018
C	-1.00328956	-3.25663527	0.07636324	C	-3.08839607	-0.96646752	-5.19407696
C	-2.32983697	-2.73598404	-0.05538704	C	-3.28463424	0.50146978	-5.12583684
C	-2.45455396	-1.63251082	0.83996490	C	-2.08711953	1.11558039	-5.68722617
C	-3.25985890	-0.50572193	0.51203544	C	-1.19538541	0.07851033	-6.12855209
C	-2.77384089	0.75907533	0.95412827	C	0.37428094	-1.46751721	4.21320170
C	-2.95005696	1.93035119	0.15964540	C	0.04468871	1.02088931	4.33441067
C	-1.80348561	2.76472180	0.35515911	C	-0.82084152	-1.66426184	5.16622239
C	-1.35254328	3.60521188	-0.70998195	C	1.66839534	-1.32852082	5.03754154
C	0.04444817	3.83740890	-0.79538068	C	-1.14816501	0.80935577	5.28707776
C	0.68897022	4.01400106	-2.07138010	C	1.34118083	1.14367530	5.15780881
C	2.01894257	3.48118649	-1.97313653	C	-0.93468374	-0.46461183	6.12183182
C	2.63528173	2.83894036	-3.09178044	C	1.55694891	-0.12913180	5.99328601
C	3.53043301	1.72572225	-2.85385726	C	0.36159134	-0.33328933	6.94287209
C	3.76152874	0.62577121	-3.79454599	N	-0.00075999	0.00261440	-2.14690304

[5,6]-open $Dy_3N@C_{80}(Ad)$, conformer _01

Dy	0.00000000	0.00000000	0.00000000
Dy	1.78193072	0.47533934	-3.06137114
Dy	-1.78472945	-0.21592947	-3.14573744
H	0.66764452	2.33229515	3.68804916
H	-0.49596255	-1.84759979	3.66369916
H	2.51695356	0.87689866	4.54454674
H	1.89499303	1.97031644	5.78850458
H	1.83722299	-1.57146789	4.53380053
H	0.72402284	-2.19274569	5.75929722
H	-1.65880487	2.04099585	4.56504427
H	-0.54995984	2.65137253	5.80088313
H	-2.34349352	-0.40736558	4.55406594
H	-1.70030734	-1.51756394	5.77096388
H	2.17569515	-0.36081872	6.69118829
H	-1.98804749	0.79891982	6.71093742
H	0.33456834	1.06809030	7.61709638
H	-0.14062031	-0.63857262	7.60641455
C	1.14746105	-0.00569040	2.29343480
C	-0.96652481	0.58373005	2.30581403
C	-0.77137825	1.82679182	1.54690244
C	0.61925399	2.20069839	1.23466927
C	1.62016972	1.16049521	1.53500906
C	2.74857430	1.01189698	0.62541529
C	3.22483475	-0.29571387	0.34683016
C	2.44824563	-1.43631986	0.77222713
C	1.31937698	-1.31377141	1.65298091
C	0.17198840	-2.17112934	1.31418688
C	-1.26593472	-1.76926892	1.32133381
C	-1.79651816	-0.44354421	1.66813727
C	-2.83707458	0.03742138	0.79979131
C	-2.92418638	1.41754196	0.39152024
C	-1.82678075	2.29113843	0.65559882
C	-1.55693731	3.28967901	-0.30909264
C	-0.21314198	3.73103918	-0.55192861
C	0.88159672	3.15255788	0.14889150
C	2.11493658	3.07241305	-0.58201481
C	3.03261923	2.00452086	-0.35042070
C	3.72248778	1.70697759	-1.59014028
C	4.14451974	0.37499678	-1.91171926
C	3.87084964	-0.61726141	-0.90701022
C	3.47938067	-1.95927612	-1.25166474
C	2.59516299	-2.44847753	-0.23141599
C	1.51525424	-3.31992815	-0.53016815
C	0.31917356	-3.16037781	0.24847631
C	-0.95258917	-3.39284252	-0.36293277
C	-1.91636228	-2.53655896	0.25732432
C	-3.02652429	-2.04955416	-0.50828125
C	-3.48629705	-0.74771147	-0.20994522
C	-4.01112952	0.12505360	-1.23537033
C	-3.64508644	1.47142317	-0.85197052
C	-3.31393975	2.42506363	-1.85169598
C	-2.29518951	3.37015140	-1.54418387
C	-1.39935407	3.86466679	-2.55207460
C	-0.11991604	4.11077139	-1.94002851
C	1.08347908	3.92422840	-2.67683169
C	2.22297667	3.44707001	-1.96748834
C	3.23231092	2.61740524	-2.60909963
C	3.17157448	2.24298477	-4.01226161
C	3.69254950	0.91038247	-4.39089868
C	4.14910331	-0.01491888	-3.31987060
C	3.63871165	-1.34515990	-3.62315366
C	3.30947648	-2.33404445	-2.61488568
C	2.29290377	-3.28566596	-2.90524292
C	1.40837977	-3.77724306	-1.87549478
C	0.12510416	-4.01124960	-2.47310377
C	-1.06769901	-3.78143006	-1.73944605
C	-2.21113803	-3.32168142	-2.47155773
C	-3.19649028	-2.47924004	-1.86957706
C	-3.81010680	-1.65803786	-2.90892927
C	-4.20552565	-0.29302856	-2.60609155
C	-3.99357658	0.71360776	-3.64180539
C	-3.47838819	2.01453266	-3.22503362
C	-2.55458133	2.49112587	-4.21189899
C	-1.49380723	3.41264728	-3.89642995
C	-0.28910520	3.31812257	-4.65769077
C	0.98962940	3.56845051	-4.05530144
C	1.97514980	2.70463543	-4.68003817
C	1.25734416	1.90677042	-5.66074365
C	1.65815954	0.56980901	-5.94335996
C	2.87210337	0.09083448	-5.30273197
C	2.87258104	-1.28007280	-4.83762035
C	1.79343314	-2.20937845	-5.09832606
C	1.54715877	-3.22781009	-4.13721301
C	0.20892382	-3.69329687	-3.87471042
C	-0.90654139	-3.14966578	-4.57439772
C	-2.15442175	-3.01867748	-3.87363830
C	-3.15299599	-2.00299486	-4.18215935
C	-2.86794176	-0.99049044	-5.19604685
C	-3.33249848	0.37410586	-4.92374034
C	-2.42855921	1.48217781	-5.22735304
C	-1.16569905	1.34308185	-5.91063094
C	-0.12947469	2.29538739	-5.65285622
C	-0.77162114	0.02489415	-6.25266567
C	-1.57894917	-1.11102219	-5.84379895
C	-0.65078103	-2.19974085	-5.60175237
C	0.68471878	-1.73981099	-5.86540671
C	0.61615876	-0.36203000	-6.27028285
C	0.08453752	0.24623649	3.35150824
C	0.42425449	1.44681503	4.28991130
C	-0.24786203	-0.96639654	4.26597604
C	1.63697186	1.09463855	5.17111855
C	0.96660276	-1.30488428	5.15374774
C	-0.78756389	1.77031638	5.18307927
C	-1.45447785	-0.63065326	5.16516894
C	1.30558810	-0.11271974	6.06263519
C	-1.12067121	0.56313824	6.07402060
C	0.09506450	0.21943092	6.95528264
N	0.00162465	-0.00622696	-2.08789112

[6,6]-open $Dy_3N@C_{80}(Ad)$, conformer _01

Dy	0.00000000	0.00000000	0.00000000
Dy	1.10115445	-1.48597172	-3.05920836
Dy	-1.20057613	1.21499502	-3.29682342
H	-0.03590995	-2.22265850	3.61023254
H	-0.24066651	2.10644458	3.63430214
H	-1.35872704	-2.29336255	5.69294230
H	-2.26927745	-1.44205874	4.43854477
H	1.16428514	-2.17362704	5.76376842
H	2.06079718	-1.23301691	4.56371803
H	-2.39014612	1.10835720	4.45271114
H	-1.56347265	2.02852355	5.71624858
H	0.95780513	2.14732556	5.78863341
H	1.94017958	1.31088395	4.57870091
H	-2.38438464	-0.18088953	6.59215783
H	1.93012787	0.02292203	6.71933878
H	-0.21199845	-0.97049358	7.56230261
H	-0.29568298	0.79907673	7.57261755
C	-0.13471329	-0.05618215	3.31883246
C	0.98939720	0.00229928	2.30885775
C	1.45574119	1.21281121	1.62301630
C	2.58166606	0.79817960	0.80177643
C	2.65340936	-0.62764815	0.79646626
C	1.56519502	-1.14894738	1.60487359
C	0.85095851	-2.31596491	1.06529942
C	-0.60087777	-2.40723850	1.10178506
C	-1.52736639	-1.33073904	1.54442279
C	-1.21144204	-0.10099414	2.24971501
C	-1.65586439	1.09951429	1.56138287
C	-0.83647415	2.26520841	1.13009075
C	0.62668077	2.32070932	1.10303864
C	1.20786246	3.09791437	0.02891134
C	2.45932456	2.75510853	-0.61012262
C	3.13757050	1.55909600	-0.27182036
C	3.86249822	0.86245496	-1.28432454
C	3.92910743	-0.57127729	-1.28991162
C	3.28424950	-1.32759363	-0.28054475
C	2.73267924	-2.58378645	-0.64651914
C	1.51130734	-3.02816464	-0.02434446
C	0.80739545	-3.79623062	-1.01430867
C	-0.61509398	-3.82492229	-1.01277554
C	-1.28977967	-3.14099855	0.04646039
C	-2.54857318	-2.51298747	-0.21456507
C	-2.65800580	-1.37848101	0.64319935
C	-3.34215057	-0.20215186	0.21664990
C	-2.77895146	1.03431164	0.64919544
C	-2.78640410	2.18238368	-0.20210965
C	-1.59210432	2.92506870	0.06749065
C	-0.98707782	3.66540892	-0.99101356
C	0.42057575	3.78225279	-0.96910887
C	1.17141657	3.85496965	-2.19649438
C	2.43458007	3.20896047	-1.97243927
C	3.08440558	2.47082460	-3.00992654
C	3.84350651	1.31517358	-2.64382626
C	3.89610167	0.15627069	-3.49402764
C	3.95405086	-1.03180553	-2.66310018
C	3.43315227	-2.31436268	-3.06893627
C	2.80803923	-3.08895897	-2.00794545
C	1.59252542	-3.86359355	-2.23779373
C	0.95566111	-3.91570164	-3.54016722
C	-0.48568175	-3.91112037	-3.50933250
C	-1.25406660	-3.92430340	-2.28150666
C	-2.51598389	-3.28437234	-2.52815489
C	-3.16268719	-2.52996982	-1.50748193
C	-3.91973551	-1.38179534	-1.89643193
C	-4.01013926	-0.22875904	-1.04328228
C	-4.04151931	0.93897518	-1.88301720
C	-3.41264786	2.15179889	-1.49458780
C	-2.88090386	2.99419348	-2.54515473
C	-1.65027756	3.69858895	-2.28016754
C	-0.91750535	3.79486074	-3.51604630
C	0.53105806	3.80228365	-3.46987803
C	1.21891629	3.13354527	-4.52252706
C	2.47708458	2.47484042	-4.30030123
C	2.53334609	1.31502837	-5.15133704
C	3.21052881	0.12325094	-4.73796316
C	2.68725693	-1.16068207	-5.15780113
C	2.85001977	-2.41238153	-4.41426351
C	1.61721227	-3.21430943	-4.65025516
C	0.76063047	-2.41629861	-5.52678800
C	-0.68598652	-2.41920416	-5.48806881
C	-1.29953987	-3.23101689	-4.50013282
C	-2.54916546	-2.85701597	-3.89841408
C	-3.21079417	-1.64242665	-4.26608877
C	-3.92699168	-0.93185574	-3.26082101
C	-4.00636689	0.51544607	-3.26965045
C	-3.46138180	1.33306153	-4.32573928
C	-2.95919016	2.67230760	-3.97444537
C	-1.70095521	3.17790705	-4.58744662
C	-0.95872387	2.36295633	-5.55521707
C	0.48066708	2.39239049	-5.51838746
C	1.30461609	1.25957079	-5.89474963
C	0.72171751	0.01241380	-6.23049785
C	1.43391886	-1.19010298	-5.86048754
C	-1.39157276	-1.24235961	-5.91110670
C	-2.64091067	-0.85917764	-5.31308462
C	-2.75157230	0.58990323	-5.34479627
C	-1.53143848	1.09905402	-5.95208524
C	-0.70054730	-0.04327461	-6.28814567
C	-0.09739539	-1.31710513	4.22898552
C	-0.21572937	1.19242566	4.24282737
C	-1.37834680	-1.37564122	5.08350570
C	1.13209295	-1.25541389	5.15566848
C	-1.49652767	1.11983758	5.09707852
C	1.01383170	1.23738630	5.16998073
C	-1.46500063	-0.13388672	5.98709031
C	1.04779023	-0.01526091	6.06101029
C	-0.23443167	-0.08094147	6.91124505
N	-0.00098697	0.00439439	-2.09422131

Magnetic properties of $Dy_2ScN@C_{80}$ and $Dy_2ScN@C_{80}(Ad)$

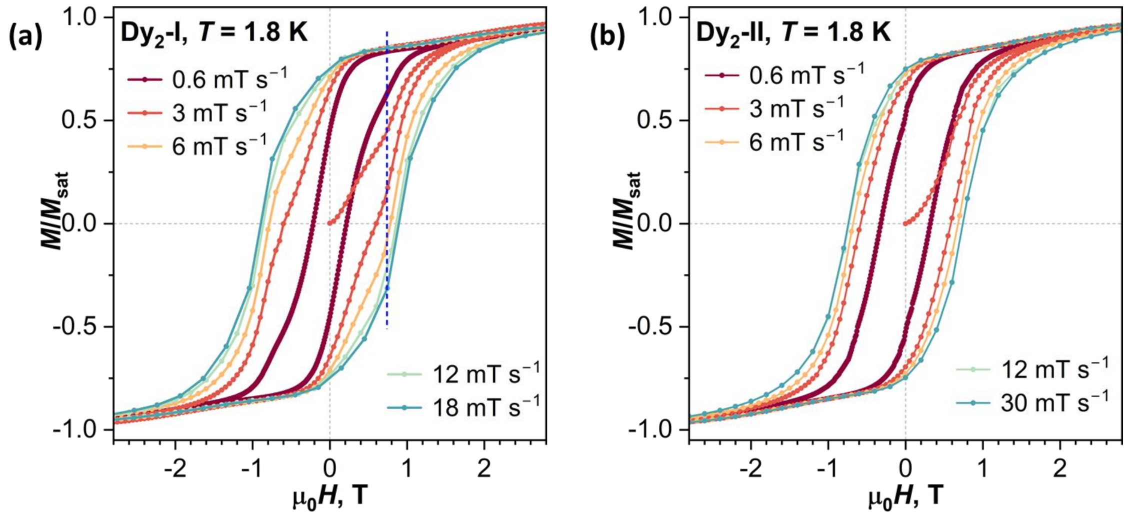

Figure S25. Magnetic hysteresis curves of **Dy_2-I** (a) and **Dy_2-II** (b) measured at 1.8 K with different sweep rates. Note that the QTM feature assigned to the level crossing in **Dy_2-I** (marked by the dashed blue line) does not change its position with the sweep rate. In **Dy_2-II**, variation of the sweep rate changes the width of the hysteresis but does not produce a visible QTM-derived feature

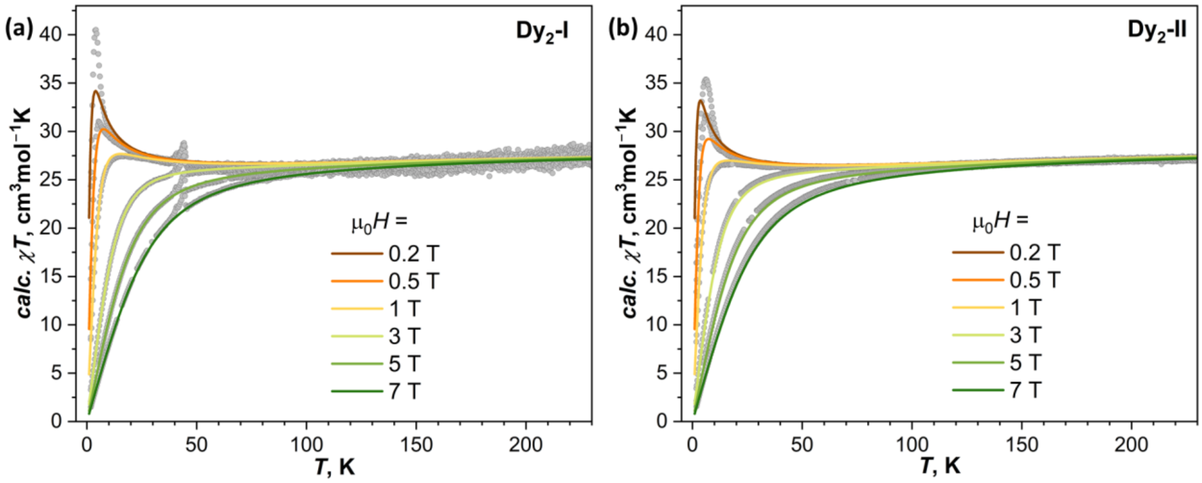

Figure S26. Experimental (dots) and simulated (colored lines) χT curves of **Dy_2-I** (a) and **Dy_2-II** (b) measured in different field; χ is defined as M/H. Note that the low-temperature part of experimental data is affected by blocking of magnetization, which can result in a deviation from thermodynamic behavior.

Simulations were performed using effective spin Hamiltonian: $\widehat{H}_{\mathrm{spin}} = \widehat{H}_{\mathrm{LF}_1} + \widehat{H}_{\mathrm{LF}_2} - 2j_{12}\widehat{J_1} \cdot \widehat{J_2} + \widehat{H}_{\mathrm{ZEE}}$, where the first two terms are single-ion ligand field Hamiltonians, and the third term describes interactions between two Dy ions. This Hamiltonian is a two-center version of Hamiltonian (2) in the main text. Ligand-field parameters and the angle between quantization axes of Dy ions were adopted from CASSCF calculations, whereas coupling constants for isomers **Dy_2-I** and **Dy_2-II** (see Table 2 in the main text) were found by matching U^{eff} values from Orbach relaxation processes.

Relaxation of magnetization in Dy_2-I

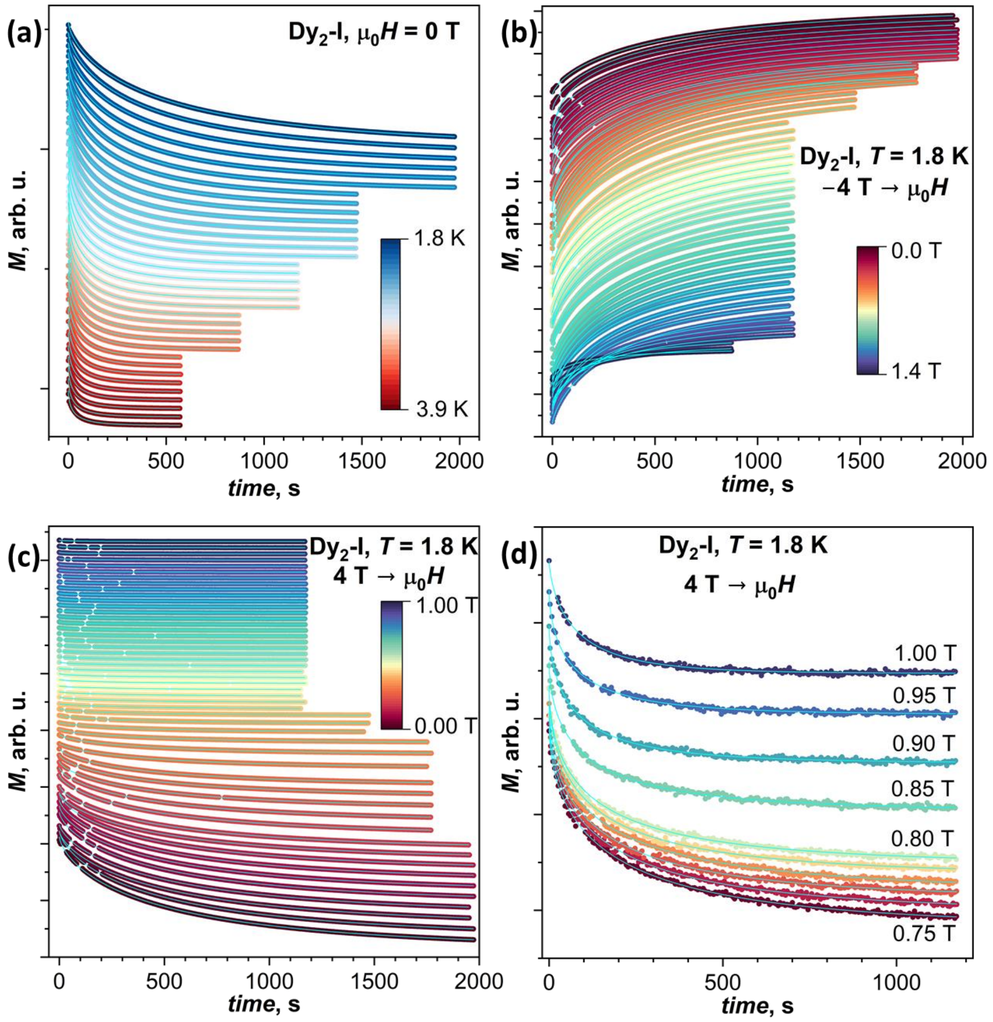

Figure S27. (a) Magnetization relaxation curves measured for **Dy_2-I** at different temperatures in zero field. (b-d) Magnetization relaxation curves measured for **Dy_2-I** at 1.8 K in different positive fields μ_0H after ramping the field from (b) −4 T (measurement of τ_-), and (c) from +4 T (measurement of τ_+); since only a small fraction of molecules is in the non-equilibrium state when measurements are performed at 0.5−1.0 T, decay curves look very flat if shown in the same scale with low-field data; (d) selected curves from (c) in the field range of 0.75−1.00 T. For clarity, curves are shown with a vertical off-set; experimental data are colored dots, fitted curves are cyan.

Table S12. Relaxation times of **Dy_2-I** measured in zero field at different temperatures.

T, K	*τ*, s	±Δ*τ*, s	β	±Δβ	*T*, K	*τ*, s	±Δ*τ*, s	β	±Δβ
1.8	513.9	0.8	0.682	0.001	2.65	90.4	1.1	0.617	0.003
1.85	445.0	0.9	0.674	0.001	2.7	85.3	1.2	0.614	0.004
1.9	387.6	0.9	0.669	0.001	2.75	79.7	1.4	0.609	0.004
1.95	340.0	1.0	0.664	0.001	2.8	81.8	1.0	0.647	0.004
2	301.7	1.1	0.661	0.002	2.85	75.9	1.3	0.636	0.005
2.05	265.6	1.1	0.650	0.002	2.9	71.8	1.1	0.631	0.004
2.1	245.2	0.9	0.673	0.002	2.95	67.7	1.2	0.625	0.005
2.15	220.8	1.0	0.666	0.002	3	61.9	1.1	0.613	0.005
2.2	196.4	1.0	0.655	0.002	3.1	63.2	0.8	0.664	0.005
2.25	178.6	1.0	0.650	0.002	3.2	57.4	0.9	0.657	0.005
2.3	161.2	1.1	0.643	0.002	3.3	50.2	0.8	0.635	0.005
2.35	146.4	1.2	0.636	0.002	3.4	46.5	0.9	0.628	0.006
2.4	132.4	1.2	0.626	0.003	3.5	44.1	1.1	0.628	0.007
2.45	120.7	1.3	0.620	0.003	3.6	40.4	1.1	0.615	0.007
2.5	117.6	1.1	0.641	0.003	3.7	35.5	1.0	0.595	0.007
2.55	110.5	1.2	0.640	0.003	3.8	33.0	1.2	0.584	0.008
2.6	101.2	1.2	0.632	0.003	3.9	31.1	1.3	0.575	0.009

Table S13. Relaxation times τ_+ of **Dy_2-I** measured at 1.8 K in different fields after fast ramp from 4 T.

H, T	τ_+, s	±Δτ_+, s	β	±Δβ	*H*, T	τ_+, s	±Δτ_+, s	β	±Δβ
0.01	523.0	1.0	0.685	0.002	0.60	302.3	2.5	0.652	0.005
0.02	520.7	0.9	0.688	0.002	0.61	301.1	3.2	0.590	0.005
0.03	546.0	1.0	0.714	0.002	0.62	338.2	3.0	0.704	0.006
0.04	542.9	0.9	0.715	0.002	0.63	284.9	3.2	0.606	0.005
0.05	512.8	0.9	0.699	0.002	0.64	274.1	2.5	0.637	0.005
0.06	506.6	0.9	0.697	0.002	0.65	267.0	3.5	0.586	0.006
0.07	504.0	0.8	0.702	0.002	0.66	275.8	4.4	0.577	0.007
0.08	498.3	0.9	0.699	0.002	0.67	260.9	3.9	0.571	0.007
0.09	522.9	1.1	0.730	0.002	0.68	229.7	3.0	0.590	0.007
0.10	518.0	1.0	0.727	0.002	0.69	226.8	2.8	0.581	0.007
0.12	477.9	0.9	0.711	0.002	0.70	228.5	3.4	0.527	0.006
0.14	468.9	0.9	0.709	0.002	0.71	212.4	3.5	0.516	0.007
0.16	465.4	0.9	0.716	0.002	0.72	209.8	3.1	0.564	0.007
0.18	458.0	0.8	0.716	0.002	0.73	195.0	3.1	0.558	0.008
0.20	450.5	0.7	0.707	0.001	0.74	175.0	2.0	0.580	0.006
0.25	468.1	0.9	0.743	0.002	0.75	178.3	2.5	0.567	0.007
0.30	435.7	1.0	0.715	0.002	0.76	161.3	2.7	0.564	0.009
0.35	450.6	1.1	0.743	0.002	0.77	148.4	2.5	0.568	0.008
0.40	432.2	1.3	0.753	0.002	0.78	153.8	3.0	0.538	0.008
0.45	396.7	1.2	0.694	0.002	0.79	127.1	1.9	0.536	0.007
0.50	384.6	1.8	0.679	0.003	0.80	129.9	2.5	0.533	0.009
0.52	395.6	2.7	0.721	0.004	0.85	106.8	2.2	0.557	0.010
0.54	346.5	2.3	0.643	0.004	0.90	78.7	1.8	0.582	0.010
0.56	385.9	3.2	0.692	0.005	0.95	75.3	1.9	0.585	0.011
0.58	342.5	2.6	0.741	0.006	1.00	82.2	1.9	0.675	0.012

Table S14. Relaxation times τ_{-} of **Dy$_2$-I** measured at 1.8 K in different fields after fast ramp from −4 T.

***H*, T**	**τ_{-}, s**	**±Δτ_{-}, s**	**β**	**±Δβ**	***H*, T**	**τ_{-}, s**	**±Δτ_{-}, s**	**β**	**±Δβ**
0.01	548.3	1.1	0.695	0.002	0.62	472.0	2.0	0.775	0.002
0.02	515.1	1.3	0.661	0.002	0.63	454.4	2.0	0.743	0.002
0.03	540.4	2.1	0.675	0.003	0.64	453.8	1.8	0.733	0.002
0.04	507.7	1.7	0.663	0.002	0.65	450.7	1.9	0.740	0.002
0.05	506.1	1.2	0.670	0.002	0.66	462.9	2.1	0.777	0.002
0.06	503.1	1.0	0.676	0.002	0.67	442.0	1.8	0.741	0.002
0.07	497.8	1.0	0.679	0.002	0.68	439.9	1.9	0.731	0.003
0.08	488.9	0.9	0.673	0.002	0.69	452.4	2.4	0.793	0.003
0.09	486.6	0.9	0.683	0.002	0.70	423.4	1.7	0.734	0.002
0.10	482.1	0.8	0.687	0.002	0.71	419.2	1.8	0.722	0.003
0.12	498.9	0.8	0.724	0.002	0.72	409.4	1.7	0.731	0.002
0.14	467.1	0.8	0.708	0.002	0.73	402.6	1.8	0.732	0.002
0.16	456.2	0.5	0.698	0.001	0.74	397.2	1.7	0.715	0.002
0.18	475.4	0.9	0.736	0.002	0.75	387.0	2.1	0.722	0.003
0.20	468.6	0.9	0.745	0.002	0.76	400.8	2.2	0.771	0.002
0.25	435.1	0.7	0.713	0.002	0.77	375.2	2.0	0.721	0.002
0.30	430.8	0.6	0.713	0.001	0.78	368.8	2.3	0.716	0.003
0.35	434.6	0.8	0.719	0.002	0.79	379.9	2.5	0.775	0.003
0.40	433.0	0.9	0.724	0.002	0.80	353.0	2.2	0.736	0.003
0.45	442.5	1.1	0.733	0.002	0.85	388.5	13.1	0.777	0.008
0.50	450.7	1.2	0.728	0.002	0.90	335.2	2.4	0.729	0.002
0.52	470.5	1.9	0.790	0.002	0.95	332.3	2.5	0.702	0.003
0.54	450.1	1.8	0.742	0.003	1.00	318.6	1.4	0.727	0.002
0.56	454.8	1.6	0.746	0.002	1.10	269.4	1.4	0.738	0.003
0.58	471.3	1.8	0.780	0.002	1.20	233.4	1.2	0.707	0.003
0.60	458.8	1.6	0.740	0.002	1.40	177.1	1.0	0.650	0.003
0.61	457.1	1.6	0.744	0.002					

Relaxation of magnetization in Dy_2-II

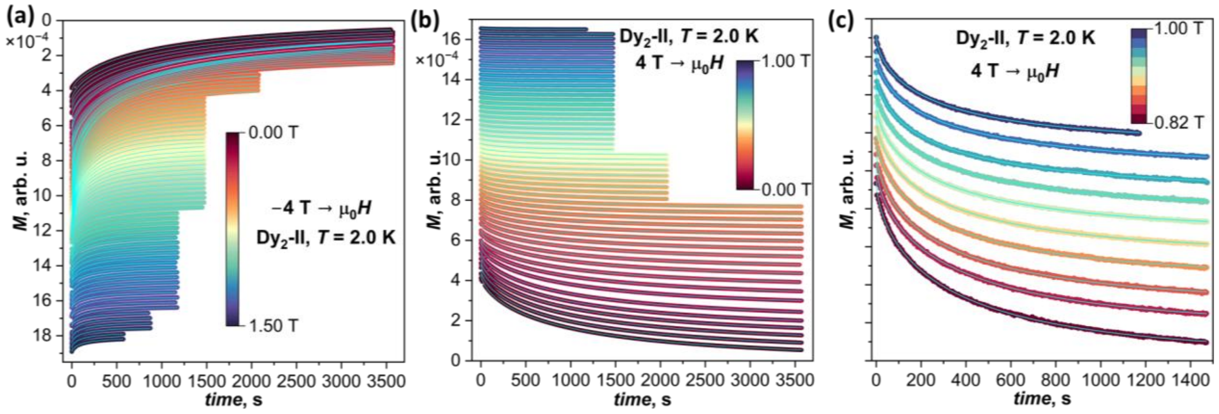

Figure S28. Magnetization relaxation curves measured for **Dy_2-II** at 2 K in different positive fields $\mu_0 H$ after ramping the field from (a) −4 T (measurements of τ_-), and (b) from +4 T (measurements of τ_+); since only a small fraction of molecules is in the non-equilibrium state when measurements are performed at 0.5−1.0 T, decay curves look very flat if shown in the same scale with low-field data; (c) selected curves from (b) in the field range of 0.82−1.00 T. For clarity, curves in (a, b) are shown with a vertical off-set; experimental data are colored dots, fitted curves are cyan.

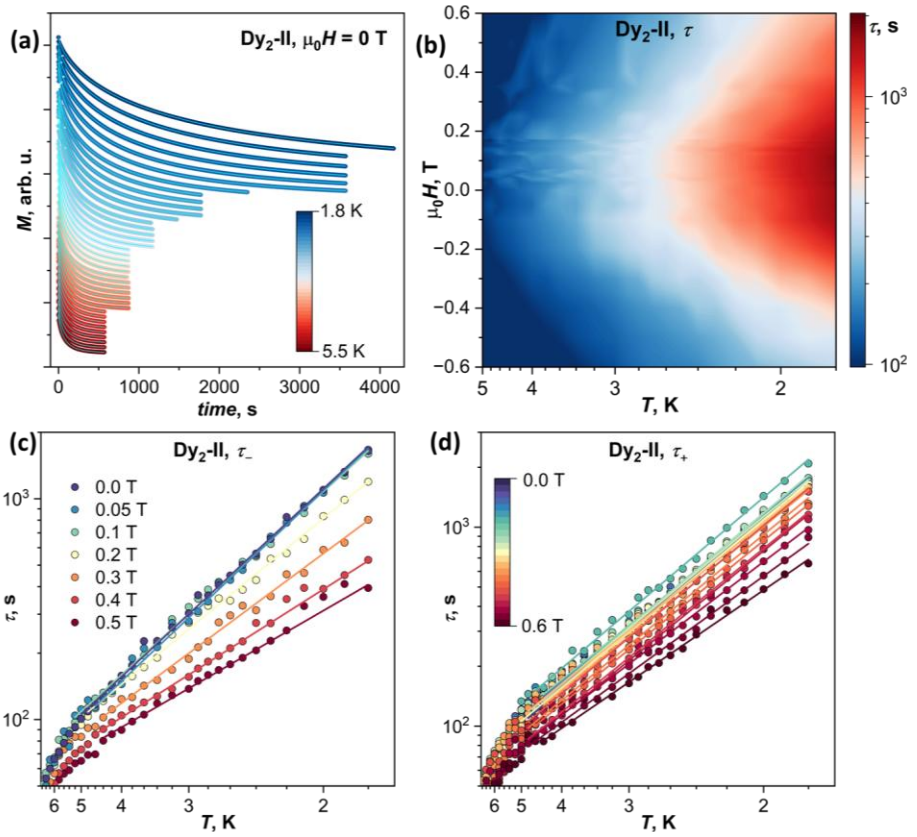

Figure S29. (a) Magnetization decay curves measured for **Dy_2-II** in zero field at different temperatures (plotted with a vertical off-set). (b) Relaxation times of **Dy_2-II** as a function of temperature and magnetic field (times in negative field are equivalent to τ_-). (c) Temperature dependence of τ_- measured at different fields and their fitting with Orbach mechanism. (d) Temperature dependence of τ_+ measured at different fields and its fitting with Orbach mechanism.

Table S15. Relaxation times of **Dy_2-II** measured at different temperatures in zero field

T, K	τ, s	±Δτ, s	β	±Δβ	T, K	τ, s	±Δτ, s	β	±Δβ
1.8	1563.6	2.1	0.646	0.001	3.7	171.9	1.1	0.681	0.004
1.9	1236.2	1.9	0.647	0.001	3.8	161.9	0.9	0.677	0.003
2.0	1010.8	1.5	0.633	0.001	3.9	152.1	0.9	0.672	0.003
2.1	842.9	1.5	0.625	0.001	4.0	166.2	0.9	0.707	0.003
2.2	708.6	1.7	0.615	0.001	4.1	136.6	1.0	0.667	0.003
2.3	596.4	1.8	0.603	0.001	4.2	130.6	1.0	0.667	0.003
2.4	563.8	1.5	0.657	0.002	4.3	122.9	1.0	0.662	0.003
2.5	461.7	1.1	0.657	0.002	4.4	118.6	1.0	0.665	0.003
2.6	410.9	1.2	0.651	0.002	4.5	110.7	0.9	0.658	0.003
2.7	368.8	1.3	0.647	0.002	4.6	106.2	0.9	0.659	0.003
2.8	325.7	1.4	0.632	0.002	4.7	106.6	0.7	0.705	0.004
2.9	304.7	1.2	0.656	0.002	4.8	101.9	0.7	0.705	0.004
3.0	306.2	1.0	0.706	0.003	4.9	96.5	0.7	0.699	0.004
3.1	256.5	1.0	0.668	0.003	5.0	93.1	0.7	0.703	0.004
3.2	237.7	1.1	0.665	0.003	5.1	88.8	0.7	0.702	0.004
3.3	220.6	1.1	0.661	0.003	5.2	84.1	0.6	0.700	0.003
3.4	205.4	1.2	0.657	0.003	5.3	81.2	0.7	0.705	0.004
3.5	190.4	1.0	0.677	0.003	5.4	77.5	0.6	0.708	0.003
3.6	179.2	1.0	0.673	0.003	5.5	73.6	0.6	0.708	0.003

Table S16. Relaxation times τ_+ of **Dy_2-II** measured at 2.0 K in different fields after fast ramp from 4 T.

H, T	τ_+, s	±Δτ_+, s	β	±Δβ	H, T	τ_+, s	±Δτ_+, s	β	±Δβ
0.00	990.1	1.5	0.632	0.001	0.49	496.5	1.4	0.637	0.002
0.01	994.0	1.5	0.637	0.001	0.50	472.6	2.1	0.662	0.002
0.02	992.7	1.2	0.632	0.001	0.52	466.5	1.9	0.663	0.002
0.03	1008.1	1.4	0.644	0.001	0.54	455.0	1.8	0.665	0.002
0.04	1011.4	1.3	0.639	0.001	0.56	445.7	2.1	0.660	0.003
0.06	1027.3	1.4	0.647	0.001	0.58	435.2	2.0	0.642	0.002
0.08	1038.7	1.4	0.649	0.001	0.60	435.1	2.1	0.642	0.003
0.10	1029.3	1.5	0.651	0.001	0.62	430.4	2.0	0.637	0.002
0.12	1049.4	1.7	0.668	0.001	0.64	411.2	2.3	0.639	0.003
0.14	985.4	1.5	0.651	0.001	0.66	404.5	1.8	0.641	0.003
0.16	995.0	1.6	0.671	0.001	0.68	395.4	1.8	0.639	0.003
0.18	915.8	1.4	0.646	0.001	0.70	390.1	2.2	0.635	0.003
0.20	883.3	1.3	0.647	0.001	0.72	375.4	1.9	0.624	0.003
0.22	840.6	1.1	0.634	0.001	0.74	369.9	1.9	0.636	0.003
0.24	808.5	1.2	0.641	0.001	0.76	353.1	1.9	0.625	0.003
0.26	776.5	1.2	0.640	0.001	0.78	352.5	4.0	0.622	0.007
0.28	745.5	1.3	0.638	0.001	0.80	345.6	2.2	0.613	0.004
0.30	713.7	1.3	0.635	0.001	0.82	343.0	2.2	0.621	0.004
0.32	680.7	1.3	0.634	0.001	0.84	341.9	2.2	0.613	0.004
0.34	628.3	1.9	0.664	0.002	0.86	328.2	2.4	0.615	0.004
0.36	606.4	1.9	0.661	0.002	0.88	320.0	2.2	0.617	0.004
0.38	587.9	1.8	0.659	0.002	0.90	314.5	2.2	0.595	0.004
0.40	570.0	1.6	0.648	0.002	0.92	317.8	2.4	0.590	0.004
0.42	554.1	1.6	0.647	0.002	0.94	313.8	2.5	0.593	0.004
0.44	537.7	1.6	0.644	0.002	0.96	297.2	3.3	0.582	0.006
0.46	526.1	1.4	0.644	0.002	0.98	329.2	3.1	0.576	0.005
0.48	516.2	1.6	0.640	0.002	1.00	285.5	3.0	0.615	0.006

Table S17. Relaxation times τ_{-} of **Dy_2-II** measured at 2.0 K in different fields after fast ramp from −4 T.

***H*, T**	**τ_{-}, s**	**±Δτ_{-}, s**	**β**	**±Δβ**	***H*, T**	**τ_{-}, s**	**±Δτ_{-}, s**	**β**	**±Δβ**
0.00	1042.7	1.6	0.652	0.001	0.80	195.2	1.2	0.591	0.002
0.01	991.6	1.5	0.631	0.001	0.82	197.8	1.2	0.630	0.003
0.02	982.0	1.5	0.620	0.001	0.84	196.1	1.1	0.613	0.003
0.03	985.9	1.6	0.615	0.001	0.86	196.5	1.1	0.607	0.003
0.04	965.0	2.1	0.596	0.001	0.88	194.4	1.1	0.608	0.003
0.06	1018.9	1.8	0.623	0.001	0.90	191.7	1.0	0.588	0.003
0.08	936.7	1.8	0.601	0.001	0.92	189.1	0.9	0.585	0.003
0.10	909.7	1.5	0.606	0.001	0.94	195.6	1.2	0.612	0.003
0.12	871.7	1.5	0.611	0.001	0.96	195.9	1.1	0.610	0.003
0.14	871.4	1.4	0.608	0.001	0.98	236.4	1.3	0.654	0.004
0.16	821.5	1.3	0.606	0.001	1.00	183.6	1.3	0.623	0.004
0.18	771.7	1.4	0.611	0.001	1.02	183.2	1.1	0.609	0.003
0.20	712.4	1.2	0.602	0.001	1.04	181.0	1.0	0.603	0.003
0.22	664.4	1.4	0.610	0.002	1.06	181.1	1.1	0.614	0.003
0.24	662.8	1.4	0.639	0.002	1.08	178.0	1.0	0.608	0.003
0.26	575.8	1.3	0.614	0.002	1.10	214.6	1.1	0.649	0.003
0.28	532.0	1.3	0.613	0.002	1.12	171.6	1.0	0.596	0.003
0.30	492.5	1.4	0.615	0.002	1.14	176.1	1.1	0.619	0.004
0.32	457.2	1.2	0.607	0.002	1.16	167.8	1.0	0.586	0.003
0.34	423.8	1.3	0.605	0.002	1.18	172.0	1.0	0.614	0.003
0.36	425.1	1.4	0.680	0.002	1.20	208.8	1.1	0.662	0.004
0.38	363.6	1.3	0.639	0.003	1.22	208.8	1.1	0.654	0.003
0.40	322.6	1.2	0.642	0.003	1.24	166.8	1.0	0.606	0.003
0.42	305.1	1.1	0.632	0.002	1.26	161.4	0.9	0.592	0.003
0.44	292.6	1.2	0.640	0.003	1.28	159.8	0.9	0.587	0.003
0.46	280.2	1.2	0.636	0.003	1.30	158.1	0.8	0.593	0.003
0.48	265.8	1.2	0.634	0.003	1.32	200.6	1.0	0.651	0.004
0.50	245.2	1.3	0.652	0.003	1.34	160.3	1.0	0.614	0.004
0.52	235.7	1.3	0.651	0.004	1.36	198.3	1.1	0.655	0.004
0.54	226.5	1.3	0.645	0.003	1.38	155.6	1.1	0.606	0.004
0.56	218.3	1.3	0.644	0.004	1.40	184.7	1.1	0.678	0.004
0.58	210.4	1.4	0.642	0.004	1.42	145.4	1.0	0.606	0.004
0.60	209.1	1.6	0.661	0.004	1.44	141.8	0.9	0.608	0.003
0.62	201.3	1.6	0.672	0.004	1.46	139.4	0.9	0.591	0.004
0.64	198.9	1.5	0.672	0.004	1.48	131.1	1.3	0.617	0.005
0.66	193.9	1.6	0.682	0.004	1.50	133.2	1.2	0.664	0.005
0.68	194.2	2.8	0.709	0.007	1.52	131.4	1.2	0.635	0.005
0.70	209.9	3.8	0.677	0.007	1.54	164.4	1.4	0.702	0.005
0.72	208.7	2.9	0.606	0.003	1.56	136.8	0.9	0.587	0.003
0.74	200.9	1.9	0.607	0.002	1.58	141.9	1.0	0.623	0.004
0.76	198.3	1.8	0.586	0.002	1.60	133.7	4.9	0.498	0.009
0.78	195.7	1.3	0.602	0.002					

Table S18a. U_+^{eff} and $\tau_{0,+}$ values for **Dy$_2$-II**, determined from temperature dependencies of τ_+ measured in different magnetic fields.

H mT	$\tau_{0,+}$ s	±Δ $\tau_{0,+}$ s	U_+^{eff} cm^{-1}	±Δ U_+^{eff} cm^{-1}	*H* mT	$\tau_{0,+}^{(\beta)}$ s	±Δ $\tau_{0,+}$ s	$U_+^{eff,(\beta)}$ cm^{-1}	±Δ U_+^{eff} cm^{-1}
0.0	22.0	0.6	5.45	0.05	0.0	21.9	0.6	5.46	0.05
25.0	20.6	0.5	5.58	0.04	25.0	20.6	0.5	5.58	0.05
37.5	21.7	0.8	5.52	0.07	37.5	21.6	0.8	5.53	0.07
50.0	24.4	0.8	5.36	0.06	50.0	24.6	0.9	5.35	0.06
62.5	23.7	0.9	5.40	0.06	62.5	24.0	1.0	5.38	0.07
75.0	22.4	0.6	5.49	0.05	75.0	22.5	0.7	5.48	0.05
100.0	27.1	1.1	5.50	0.08	100.0	27.3	1.2	5.48	0.08
112.5	23.0	0.7	5.47	0.06	112.5	23.0	0.7	5.47	0.06
125.0	25.9	0.7	5.25	0.05	125.0	26.0	0.7	5.25	0.05
137.5	22.3	0.7	5.51	0.05	137.5	22.2	0.7	5.52	0.06
150.0	23.5	0.7	5.38	0.06	150.0	23.4	0.7	5.39	0.06
162.5	22.4	0.6	5.40	0.05	162.5	22.3	0.6	5.42	0.06
169.0	22.4	0.8	5.36	0.06	169.0	22.4	0.9	5.37	0.07
175.0	25.3	0.9	5.17	0.06	175.0	25.3	0.9	5.17	0.06
200.0	22.5	0.6	5.28	0.04	200.0	22.5	0.6	5.28	0.05
250.0	19.9	0.8	5.32	0.07	250.0	19.8	0.8	5.33	0.08
275.0	19.6	0.4	5.25	0.03	275.0	19.6	0.4	5.25	0.03
300.0	20.9	0.5	5.40	0.05	300.0	20.8	0.5	5.41	0.05
325.0	17.8	0.4	5.25	0.04	325.0	17.8	0.4	5.26	0.04
350.0	17.0	0.7	5.28	0.07	350.0	16.9	0.7	5.29	0.07
400.0	19.3	0.7	4.93	0.06	400.0	19.3	0.7	4.93	0.06
500.0	19.3	0.6	4.70	0.05	500.0	19.6	0.6	4.67	0.05
600.0	19.8	0.6	4.44	0.05	600.0	19.8	0.6	4.44	0.05

Table S18b. U_-^{eff} and $\tau_{0,-}$ values for **Dy$_2$-II**, determined from temperature dependencies τ_- measured in different magnetic fields.

H mT	$\tau_{0,-}$ s	±Δ $\tau_{0,-}$ s	U_-^{eff} cm^{-1}	±Δ U_-^{eff} cm^{-1}	*H* mT	$\tau_{0,-}^{(\beta)}$ s	±Δ $\tau_{0,-}$ s	$U_-^{eff,(\beta)}$ cm^{-1}	±Δ U_-^{eff} cm^{-1}
0	22.0	0.6	5.45	0.05	0	21.9	0.6	5.46	0.05
50	20.5	0.7	5.52	0.06	50	20.6	0.7	5.52	0.06
100	23.6	0.7	5.31	0.06	100	23.1	0.7	5.35	0.06
200	25.6	1.0	4.81	0.07	200	25.2	0.9	4.85	0.07
300	25.0	1.2	4.33	0.08	300	24.8	1.0	4.35	0.08
400	26.9	0.4	3.71	0.02	400	27.2	0.4	3.69	0.03
500	27.4	0.6	3.38	0.04	500	26.9	0.7	3.42	0.05

Two sets of data are shown in each table: on the left, fitting was performed for ln($\tau_\pm$) values without weighting, whereas the values on the right utilized uncertainty in the form:[16, 17]

$$\sigma_{\ln(\tau)}^2 = \frac{\pi^2}{6}(\beta^{-2} - 1)$$

where β is the parameter from the stretched exponential fitting of relaxation times. Two sets of values appeared very similar, with variations of $\tau_\pm$ and $U_\pm^{eff}$ values between the sets of less than 0.03 s and 0.02 cm^{-1}, respectively.

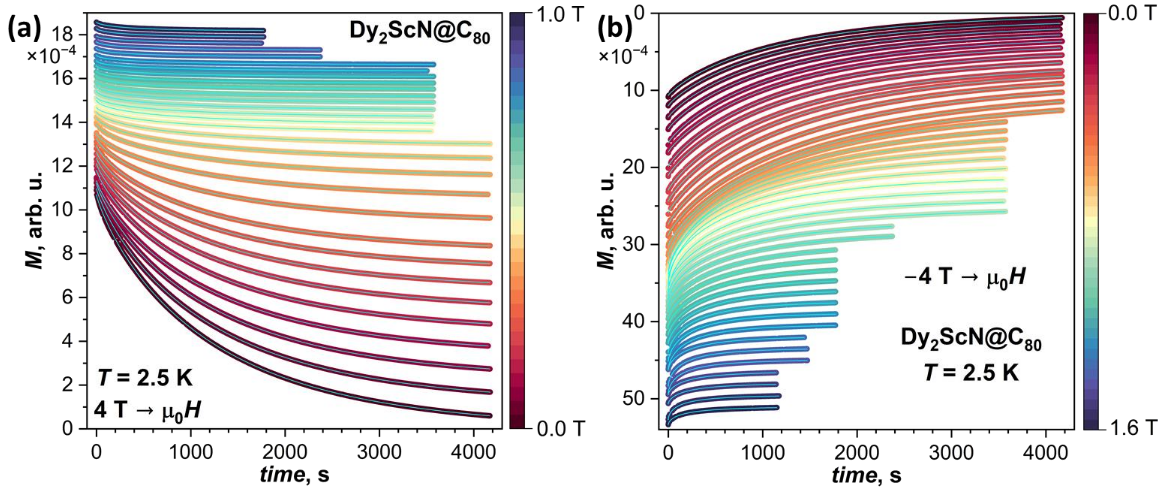

Figure S30. Magnetization decay curves of $Dy_2ScN@C_{80}$ powder measured in different positive fields after ramping the field from (a) +4 T (measurement of τ_+), and (b) from –4 T (measurement of τ_-). For clarity, curves are shown with a vertical off-set; experimental data are colored dots, fitted curves are cyan.

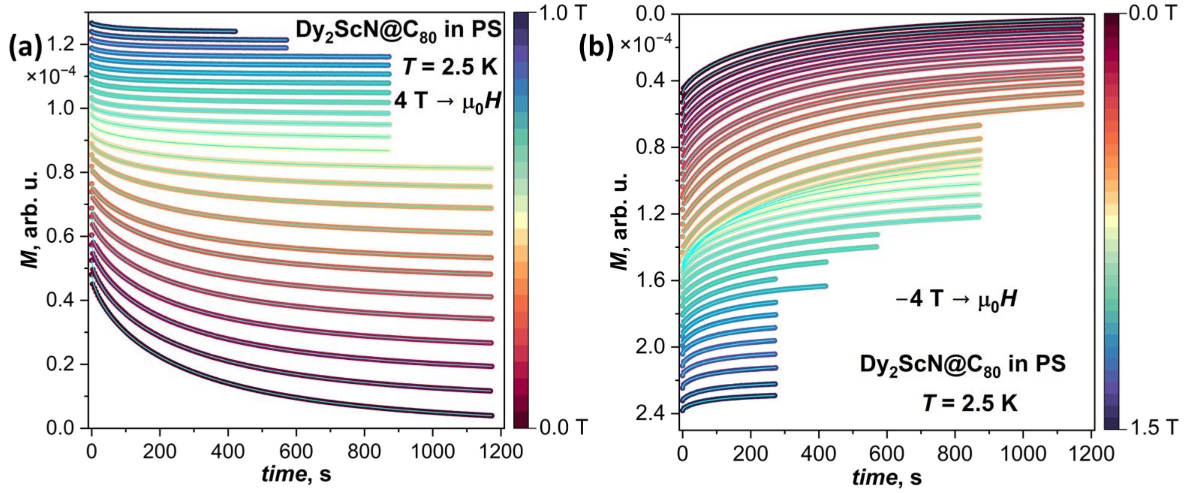

Figure S31. Magnetization decay curves of $Dy_2ScN@C_{80}$ diluted in polystyrene measured in different positive fields after ramping the field from (a) +4 T (measurement of τ_+), and (b) from –4 T (measurement of τ_-). For clarity, curves are shown with a vertical off-set; experimental data are colored dots, fitted curves are cyan.

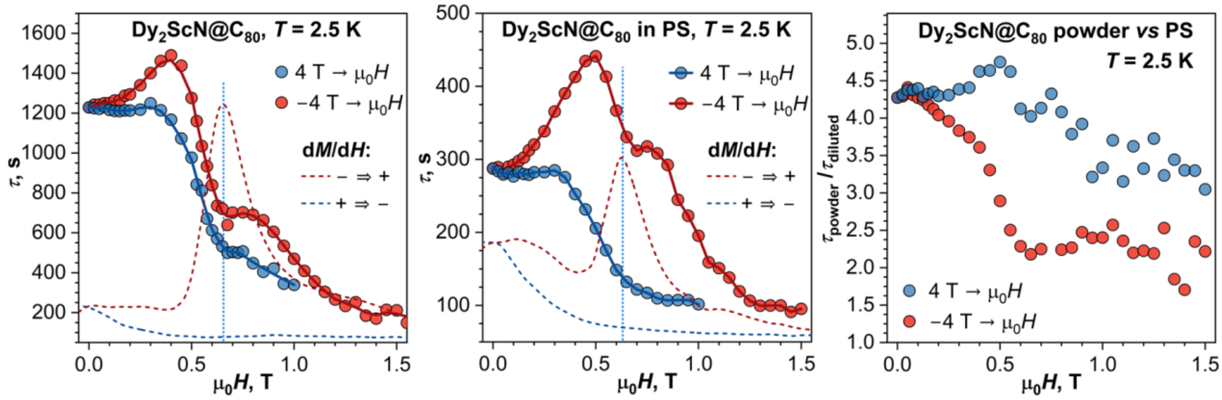

Figure S32. Field dependence of τ_+ (blue) and τ_- (red) in $Dy_2ScN@C_{80}$ powder and $Dy_2ScN@C_{80}$ diluted in polystyrene (same as Figure 7d-e in the main text), and ratio of the values for powder and PS-diluted sample (right).

Table S19. Relaxation times τ_+ of $Dy_2ScN@C_{80}$ powder measured at 2.5 K in different fields after fast ramp from 4 T.

H, T	τ_+, s	$\pm\Delta\tau_+$, s	β	±Δβ	*H*, T	τ_+, s	$\pm\Delta\tau_+$, s	β	±Δβ
0	1228.6	0.4	0.860	0.000	525	841.7	1.5	0.608	0.001
25	1225.0	0.5	0.857	0.001	550	811.3	1.7	0.630	0.002
50	1222.0	0.4	0.858	0.001	575	671.3	1.6	0.592	0.002
75	1232.1	0.4	0.859	0.000	600	612.6	2.3	0.574	0.002
100	1216.7	0.7	0.844	0.001	625	570.0	1.9	0.572	0.002
125	1211.7	0.9	0.833	0.001	650	531.9	2.3	0.563	0.002
150	1210.6	0.9	0.829	0.001	675	500.0	2.4	0.553	0.002
175	1214.0	1.0	0.818	0.001	700	503.5	2.8	0.557	0.003
200	1213.4	1.3	0.801	0.001	725	500.4	2.7	0.538	0.003
250	1217.7	1.5	0.777	0.001	750	506.8	2.7	0.546	0.003
300	1249.2	1.2	0.774	0.001	800	447.9	3.1	0.567	0.004
350	1214.3	2.0	0.708	0.001	850	404.8	1.9	0.592	0.003
400	1167.0	1.9	0.676	0.001	900	420.9	2.6	0.670	0.004
450	1072.9	1.5	0.649	0.001	950	344.7	1.6	0.631	0.003
500	976.6	1.8	0.650	0.001	1000	339.1	1.5	0.678	0.003

Table S20. Relaxation times τ_- of $Dy_2ScN@C_{80}$ powder measured at 2.5 K in different fields after fast ramp from −4 T.

H, mT	τ_-, s	$\pm\Delta\tau_-$, s	β	±Δβ	*H*, mT	τ_-, s	$\pm\Delta\tau_-$, s	β	±Δβ
0	1229.0	0.4	0.860	0.000	675	639.2	5.4	0.647	0.004
25	1241.5	0.6	0.862	0.000	700	701.4	6.0	0.749	0.003
50	1242.8	0.4	0.868	0.000	750	702.6	2.1	0.660	0.001
75	1249.8	0.5	0.871	0.001	800	690.5	2.2	0.670	0.001
100	1250.7	0.6	0.864	0.001	850	663.2	1.7	0.689	0.001
125	1263.1	0.3	0.875	0.000	900	605.3	2.0	0.713	0.001
150	1263.5	0.6	0.869	0.001	950	534.7	1.2	0.739	0.001
175	1287.2	0.3	0.874	0.000	1000	469.4	1.3	0.735	0.002
200	1294.2	0.3	0.865	0.000	1050	409.1	1.1	0.738	0.002
250	1339.8	0.4	0.860	0.000	1100	356.1	1.1	0.724	0.002
300	1400.9	0.4	0.854	0.000	1150	303.7	1.1	0.692	0.003
350	1461.3	0.4	0.843	0.000	1200	265.3	1.1	0.673	0.003
400	1490.0	0.5	0.832	0.000	1250	233.1	1.1	0.652	0.003
450	1437.5	1.0	0.813	0.001	1300	252.0	1.2	0.673	0.003
500	1276.2	0.7	0.772	0.000	1350	184.2	1.2	0.622	0.004
525	1159.9	0.7	0.746	0.000	1400	170.1	1.1	0.596	0.003
550	1034.7	0.5	0.716	0.000	1450	213.9	1.4	0.661	0.004
575	933.6	0.9	0.714	0.001	1500	211.2	1.3	0.645	0.004
600	838.7	1.1	0.694	0.001	1550	149.1	1.1	0.581	0.004
625	735.9	1.0	0.645	0.001	1600	190.2	1.2	0.608	0.004
650	720.4	2.5	0.689	0.002					

Table S21. Relaxation times τ_+ of $Dy_2ScN@C_{80}$ diluted in polystyrene and measured at 2.5 K in different fields after fast ramp from 4 T.

H, T	τ_+, s	±Δτ_+, s	β	±Δβ	*H*, T	τ_+, s	±Δτ_+, s	β	±Δβ
0	287.3	0.6	0.694	0.002	450	231.4	1.7	0.584	0.003
25	283.8	0.5	0.693	0.002	500	205.6	1.7	0.580	0.004
50	279.3	0.4	0.694	0.001	550	175.5	1.7	0.581	0.005
75	281.6	0.5	0.700	0.002	600	148.5	1.5	0.590	0.006
100	276.7	0.5	0.684	0.002	650	132.1	1.4	0.582	0.006
125	282.8	0.6	0.696	0.002	700	121.8	1.4	0.602	0.007
150	279.9	0.5	0.682	0.002	750	117.2	1.2	0.621	0.006
175	279.1	0.6	0.683	0.002	800	109.7	1.3	0.607	0.007
200	282.3	0.7	0.666	0.002	850	106.9	1.5	0.628	0.009
250	282.6	0.8	0.663	0.002	900	107.3	1.4	0.662	0.009
300	284.9	0.9	0.633	0.002	950	107.3	1.8	0.669	0.011
350	275.6	1.1	0.630	0.003	1000	101.6	1.7	0.710	0.012
400	252.5	1.7	0.624	0.003					

Table S22. Relaxation times τ_- of $Dy_2ScN@C_{80}$ diluted in polystyrene and measured at 2.5 K in different fields after fast ramp from −4 T.

H, mT	τ_-, s	±Δτ_-, s	β	±Δβ	*H*, mT	τ_-, s	±Δτ_-, s	β	±Δβ
0	287.6	0.5	0.695	0.001	700	311.9	2.3	0.688	0.004
25	289.2	4.1	0.684	0.008	750	317.8	2.3	0.692	0.003
50	281.9	5.1	0.671	0.011	800	308.2	2.3	0.714	0.003
75	289.8	1.1	0.697	0.003	850	292.4	2.1	0.729	0.003
100	292.7	2.3	0.692	0.007	900	244.6	2.5	0.776	0.004
125	297.8	1.0	0.705	0.003	950	222.8	2.5	0.777	0.005
150	302.4	0.6	0.703	0.002	1000	182.1	4.7	0.795	0.010
175	312.4	0.8	0.702	0.003	1050	148.7	4.2	0.807	0.008
200	320.2	0.9	0.699	0.003	1100	140.8	4.4	0.783	0.012
250	338.5	1.3	0.698	0.004	1150	137.8	14.0	0.747	0.023
300	365.5	0.9	0.700	0.002	1200	132.5	22.3	0.758	0.036
350	390.4	1.2	0.703	0.003	1250	218.8	46.4	0.606	0.018
400	412.9	1.8	0.712	0.003	1300	224.5	49.1	0.549	0.015
450	434.7	3.3	0.711	0.005	1350	149.4	18.8	0.584	0.013
500	441.2	2.6	0.712	0.004	1400	237.8	52.9	0.498	0.013
550	413.3	2.0	0.702	0.003	1450	107.0	10.0	0.699	0.019
600	366.9	1.4	0.697	0.003	1500	135.6	15.5	0.583	0.012
650	330.6	3.2	0.696	0.006					

Magnetic properties of $Dy_3N@C_{80}$ and $Dy_3N@C_{80}(Ad)$

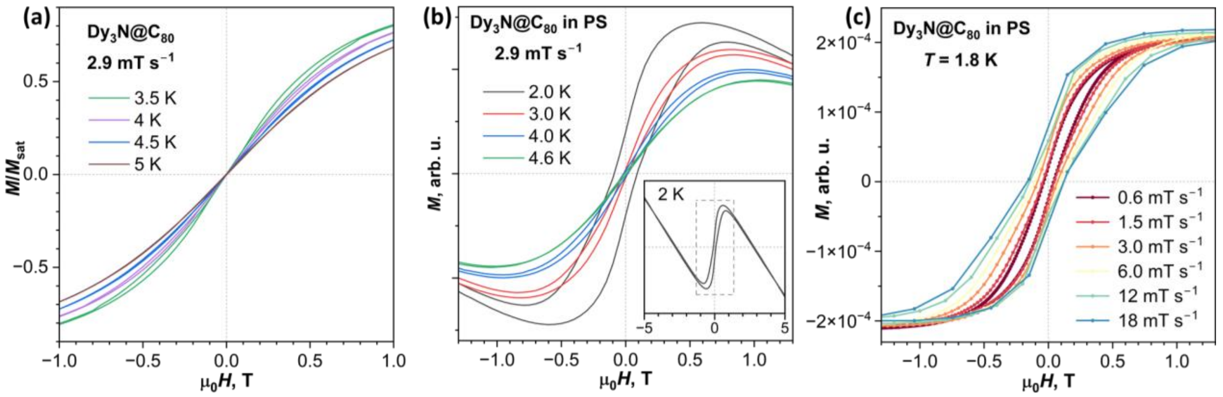

Figure S33. (a) Magnetic hysteresis measurements of powder $Dy_3N@C_{80}$ at 3.5−5 K; narrow opening is still detectable at 4.5 K but not at 5.0 K. (b) Magnetic hysteresis measurements of $Dy_3N@C_{80}$ diluted in polystyrene at 2.0−4.6 K; the strong diamagnetic background is caused by PS matrix (see also inset with a broader field range). (c) Magnetic hysteresis of $Dy_3N@C_{80}$ diluted in polystyrene and measured at 1.8 K with different field sweep rates; linear background is subtracted.

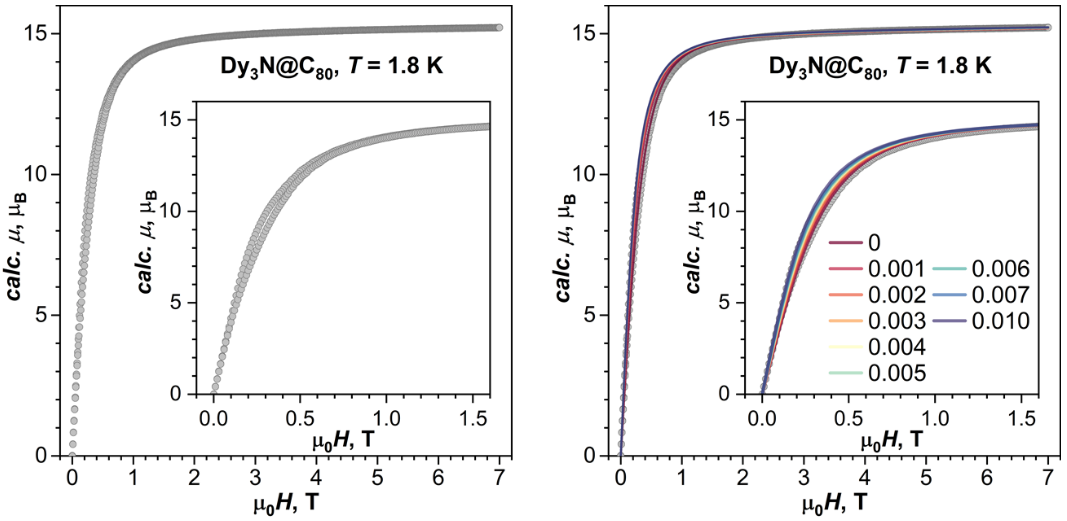

Figure S34. (a) Magnetization curve of $Dy_3N@C_{80}$ measured at 1.8 K with the sweep rate of 0.6 mT s^{-1}; inset shows that hysteresis is still open, but is much narrower than at faster sweeps. (b) The same experimental data as in (a) overlaid with simulated magnetization curves obtained with different *j* values (0−0.05 cm^{-1} with the step of 0.005 cm^{-1} for the main plot, and 0−0.01 cm^{-1} with the step of 0.001 cm^{-1} for the inset). Variation of magnetization curves of $Dy_3N@C_{80}$ with *j* is less pronounced than the variation of χT curves (Figure S23).

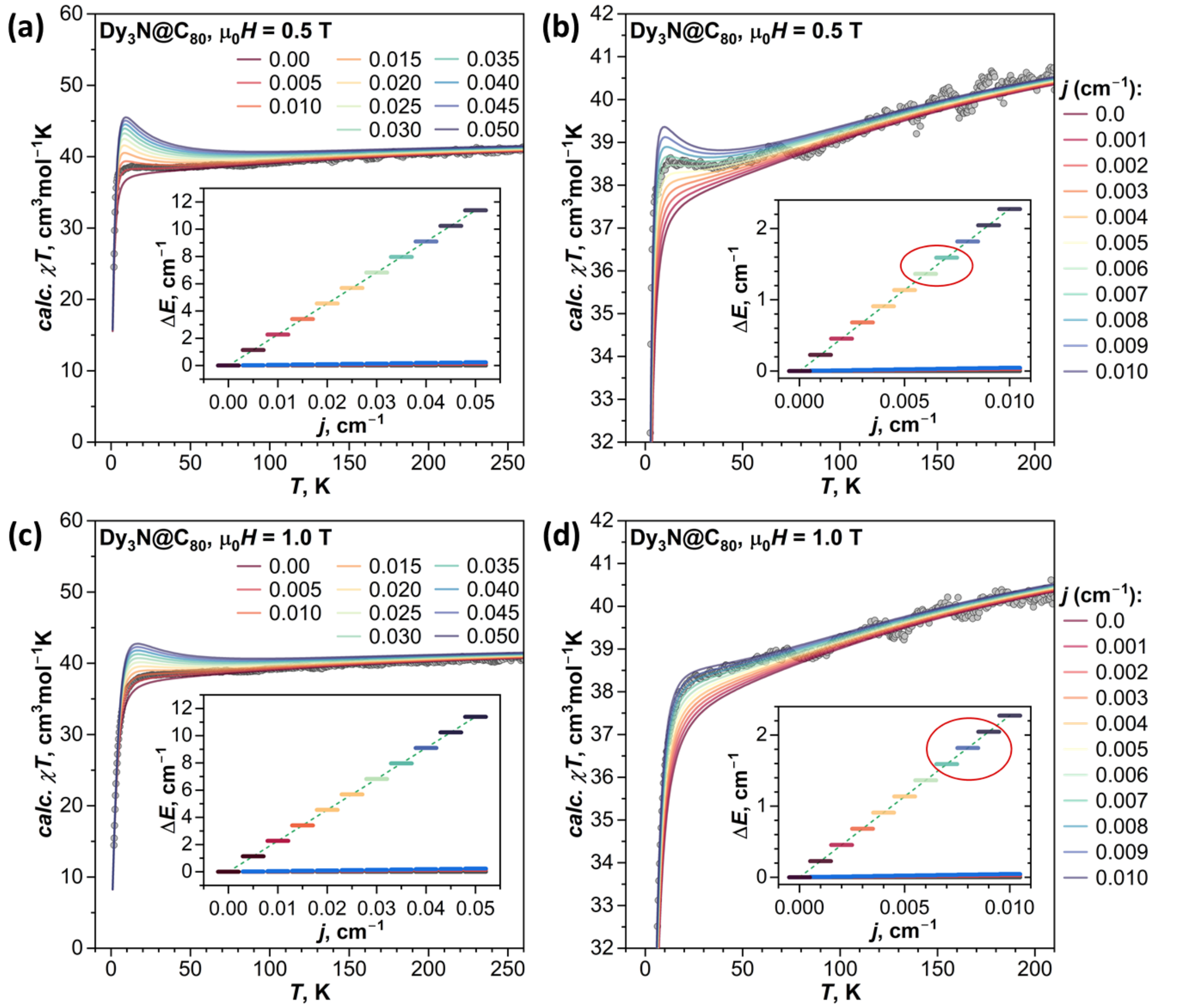

Figure S35. (a) Experimental χT curve of $Dy_3N@C_{80}$ measured in the field of 0.5 T compared to simulations with j values in the range of 0–0.05 cm^{-1}, step 0.005 cm^{-1}; inset shows variation of the energy splitting for exchange states with j. (b) Enlargement of experimental data in the low-field region and comparison to simulations for j values in the range of 0–0.01 cm^{-1}. Note the change of the scale in (b) in comparison to (a), which leads to a more apparent worsening of the signal-to-noise ratio with increasing temperature. (c,d) The same as (a,b) but for χT data measured in the field of 1.0 T.

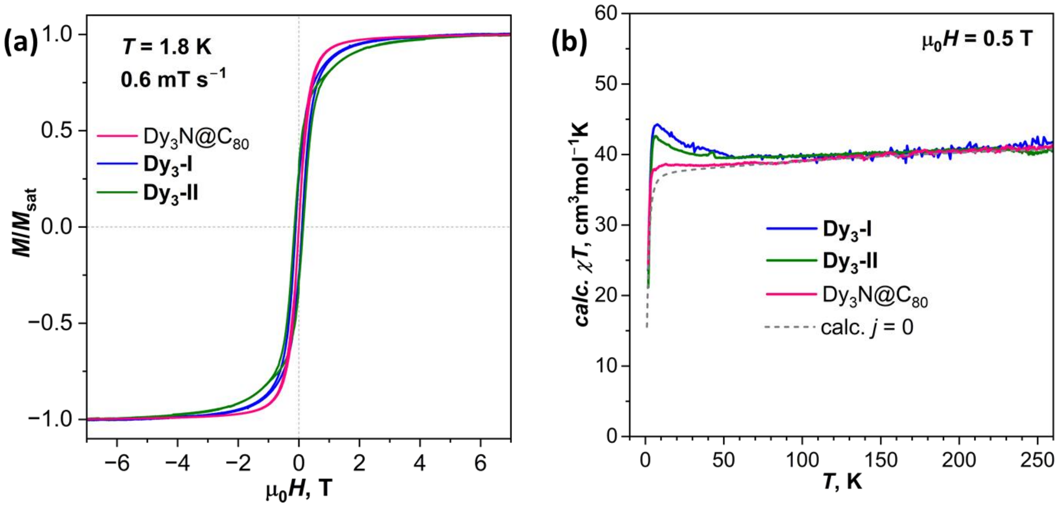

Figure S36. (a) Magnetization curves of **Dy$_3$-I**, **Dy$_3$-II**, and Dy$_3$N@C$_{80}$ measured with slow sweep rate of 0.6 mT s^{-1}at 1.8 K. (b) χT curves of **Dy$_3$-I**, **Dy$_3$-II**, and Dy$_3$N@C$_{80}$ measured in the field of 0.5 T (χ is defined as M/H); also shown is the calculated curve for Dy$_3$N@C$_{80}$ with non-interacting Dy moments.

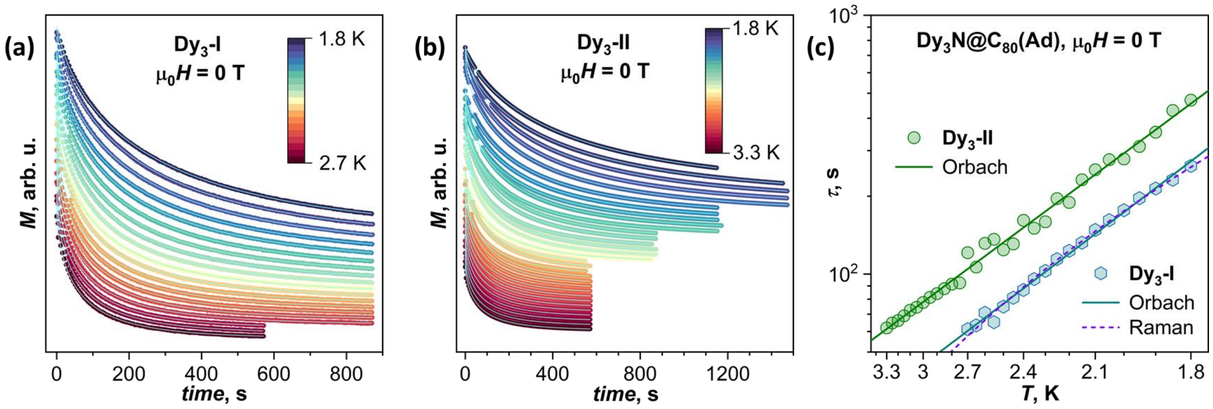

Figure S37. (a, b) Magnetization decay curves of **Dy$_3$-I** (a) and **Dy$_3$-II** (b) measured in zero field at different temperatures; curves are shown with a vertical off-set; experimental data are colored dots, fitted curves are cyan. (c) Relaxation times of **Dy$_3$-I** and **Dy$_3$-II** as a function of temperature and their fitting with Orbach mechanism (both isomers) and Raman mechanism (**Dy$_3$-I** only). Fitted parameters for Orbach mechanism: τ_0 = 3.0(2) s and U^{eff} = 8.1(2) K for **Dy$_3$-I**, τ_0 = 5.5(3) s and U^{eff} = 7.9(2) K for **Dy$_3$-II**. Fitted parameters for Raman mechanism in **Dy$_3$-I**, $\tau^{-1} = cT^n$: c = 4.4(2) 10^{-4} s^{-1}K^{-n}, n = 3.7(1). Raman mechanism describes experimental data of **Dy$_3$-I** somewhat better than the Orbach mechanism, but deviations are not substantial enough to make unequivocal assignment.

Dy···Dy coupling constants in $Dy_3N@C_{80}$(Ad) isomers

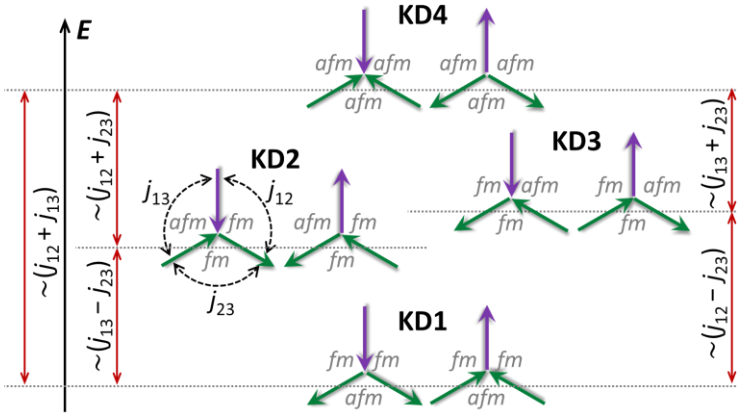

Figure S38. Schematic description of four exchange Kramers doublets (KDs) and relations between their energies and j_{ij} constants in Hamiltonian (2) of the main text.

$$\hat{H}_{\text{spin}} = \sum_i \hat{H}_{\text{LF}_i} - 2j_{12}\hat{J}_1 \cdot \hat{J}_2 - 2j_{13}\hat{J}_1 \cdot \hat{J}_3 - 2j_{23}\hat{J}_2 \cdot \hat{J}_3 + \hat{H}_{\text{ZEE}}$$

A disadvantage of this Hamiltonian form is that the space for full diagonalization is large (4096 states), which makes calculations rather lengthy and precludes brute force fitting of all coupling constants since the number of iterations in such fittings is usually quite large. We therefore decided to estimate the values by simulations of magnetization and χT curves with variable constants. Note that DFT and CASSCF calculations showed that angles between quantization axes of Dy ions are not strictly 120°, but rather α_{12} = 117.8°, α_{13} = 120.4°, α_{23} = 121.2° in **Dy_3-I** and α_{12} = 117.4°, α_{13} = 125.3°, α_{23} = 116.8° in **Dy_3-II**. Since the energies of exchange interactions in Hamiltonian (2) depend not only on the coupling constants but also on the angles between the frames of operators $\hat{J}_i$ as $\Delta E_{ij} = 225 j_{ij}\cos(\alpha)$, KD2 and KD3 energies are split even when $j_{12} = j_{13}$.

Magnetization curves are most sensitive to the values of coupling constants at low temperature. However, at 1.8 K both $Dy_3N@C_{80}$(Ad) isomers exhibit magnetic hysteresis even when measurements were performed with the slow sweep rate of 0.6 mT s^{-1}.We therefore relied on comparison between experiment and simulations in the field range where the hysteresis is already closed. When hysteresis is open, experimental equilibrium magnetization curve is not known, but it should remain within the opening. Therefore, for such regions, we expected for simulated curve to pass between the branches of experimental curves.

Figures S39−S42 demonstrate series of simulations, which allowed to find the set of coupling constants providing the best match to experimental data for **Dy_3-II**. At first, we fixed all constants equal and demonstrated that the values of j have significant influence on the shape of χT curves, whereas magnetization curves at 1.8 K are almost identical for different j (except for j = 0) (Figure S39). The weak dependence of magnetization curves on the j value indicates that coupling constants in **Dy_3-II** cannot be equal, else magnetization curve cannot be reproduced. That the coupling constants should be different is also suggested based on the QTM argument, as discussed in the main text.

Then, we fixed $j_{12} = j_{13}$ = 0.05 cm^{-1} and varied j_{23}. The shape of magnetization curves has more pronounced dependence on j_{23} (and thus on the difference between j_{12} and j_{23}) than χT (Figure S40). Experimental magnetization curve is reasonably well matched by the (j_{12}, j_{13}, j_{23}) values of (0.05, 0.05, 0.0) cm^{-1}, but this set of constants does not match experimental χT curve. The latter is to a large extent determined by the energy of KD4 (j_{12}+j_{13}, Figure S38), hence at the next step we varied the j_{12} value while keeping the difference between j_{12} and j_{23} constant and equal to 0.05 cm^{-1}. Variation of j_{12} had

nearly no effect on magnetization curves, but had a clear influence on the shape of χT curves (Figure S41). The best match to experimental data was obtained for the (j_{12}, j_{13}, j_{23}) values of (0.035, 0.035, −0.015) cm^{-1}. As the agreement between experimental and simulated magnetization curve was not perfect, we decided to split j_{12} and j_{13} constants (which were kept equal in all previous simulations). Their sum was kept constant, and we just varied their difference. Splitting of j_{12} and j_{13} values had a weak but distinct influence on the shape of χT and magnetization curves (Figure S42). The best match to experimental data was obtained for the (j_{12}, j_{13}, j_{23}) values of (0.028, 0.042, −0.015) cm^{-1}, which give the energies of exchange states KD1−KD4 of 0.0, 4.3, 7.1, and 8.2 cm^{-1} (Figure 8c in the main text).

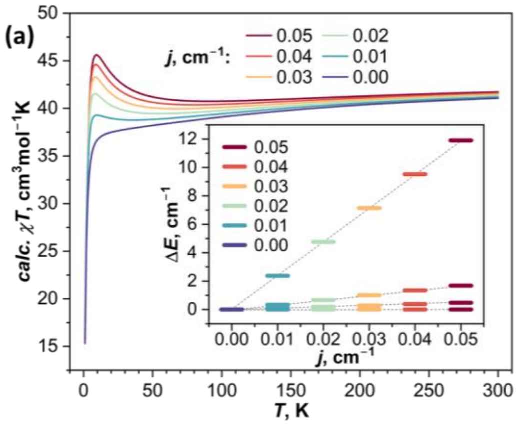

Figure S39. Simulated χT (a) and magnetization curves (b) for **Dy_3-II** with different values of coupling constant j ($j = j_{12} = j_{13} = j_{23}$); χT is simulated for the magnetic field of 0.5 T, magnetization curve for T = 1.8 K. Insets show variation of the energy levels with j. The values of j have significant influence on the shape of χT curves, whereas magnetization curves at 1.8 K are almost identical for different j (except for j = 0). The weak dependence of magnetization curves on j value indicates that coupling constants in **Dy_3-II** cannot be equal, else magnetization curve cannot be reproduced. That the coupling constants should be different is also suggested based on the QTM argument, as discussed in the main text.

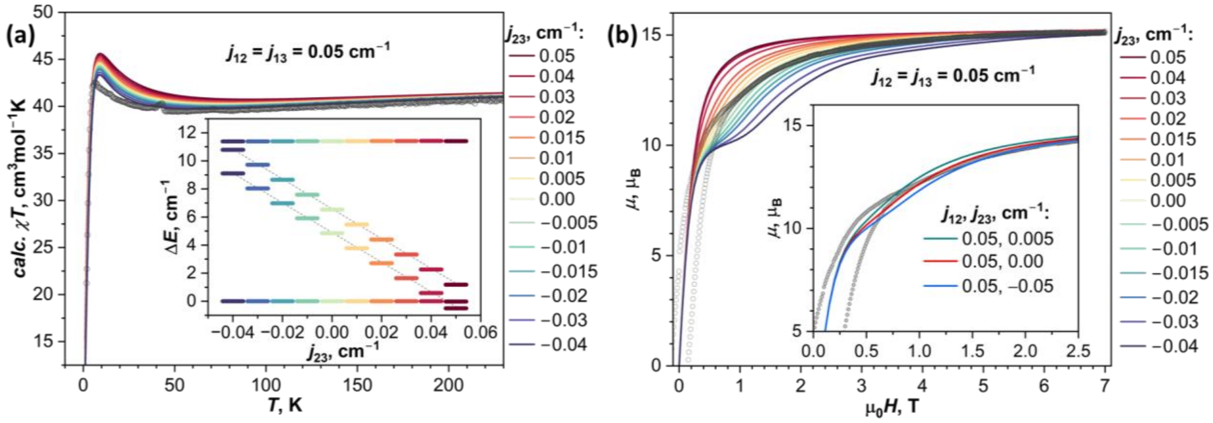

Figure S40. Simulated χT (a) and magnetization curves (b) for **Dy_3-II** with $j_{12} = j_{13}$ = 0.05 cm^{-1} and different values of j_{23}. χT is simulated for the magnetic field of 0.5 T, magnetization curve for T = 1.8 K; experimental data are shown as gray dots. The shape of magnetization curves has more pronounced dependence on j_{23} (and thus on the difference between j_{12} and j_{23}) than χT. Experimental magnetization curve is reasonably well matched by the (j_{12}, j_{13}, j_{23}) values of (0.05, 0.05, 0.0) cm^{-1}, but this set of constants does not match experimental χT curve. Since the latter is to a large extent determined by the energy of KD4~(j_{12}+j_{13}), at the next step we varied the j_{12} value while keeping the difference between j_{12} and j_{23} constant and equal to 0.05 cm^{-1}.

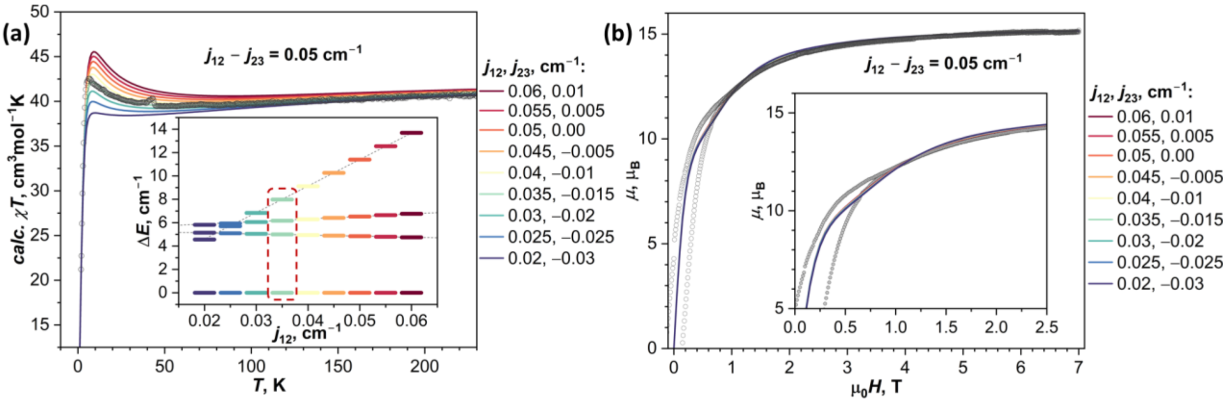

Figure S41. Simulated χT (a) and magnetization curves (b) for **Dy$_3$-II** with the constant difference $j_{12} - j_{23}$ = 0.05 cm^{-1}. χT is simulated for the magnetic field of 0.5 T, magnetization curve for T = 1.8 K; experimental data are shown as gray dots. Variation of j_{12} has nearly no effect on magnetization curves, but has a clear influence on the shape of χT curves. The best match to experimental data is obtained for the (j_{12}, j_{13}, j_{23}) values of (0.035, 0.035, −0.015) cm^{-1}.

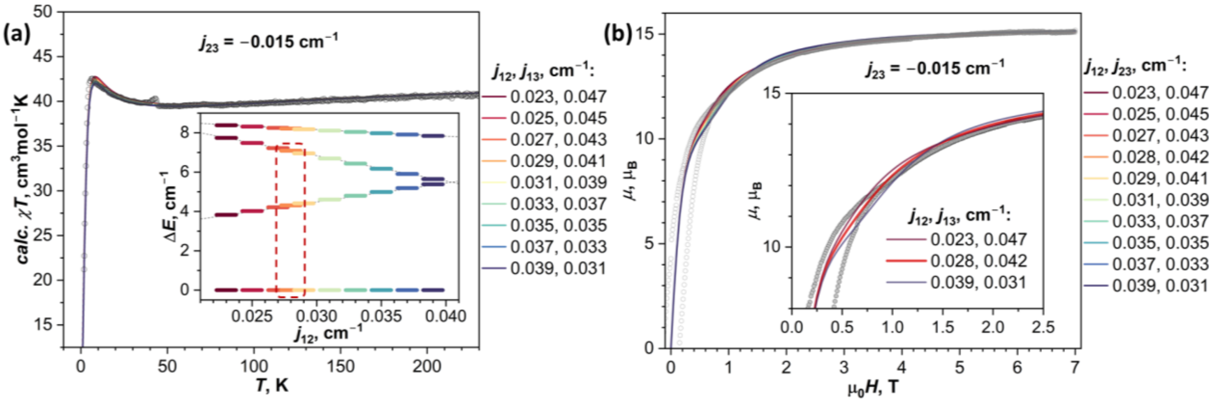

Figure S42. Simulated χT (a) and magnetization curves (b) for **Dy$_3$-II** with the constant j_{23} = −0.015 cm^{-1}, and constant sum of j_{12} +j_{13} = 0.07 cm^{-1}, whereas individual values of j_{12} and j_{13} are varied. χT is simulated for the magnetic field of 0.5 T, magnetization curve for T = 1.8 K; experimental data are shown as gray dots. Splitting of j_{12} and j_{13} values has a weak but distinct influence on the shape of χT and magnetization curves. The best match to experimental data is obtained for the (j_{12}, j_{13}, j_{23}) values of (0.028, 0.042, −0.015) cm^{-1}.

For **Dy_3-I** we partially relied on the result obtained for **Dy_3-II**. Simulations of magnetization curves with the $j_{12} = j_{13}$ value of 0.05 cm^{-1} and variable j_{23} (Figure S43, inset) demonstrated that the difference between j_{12} and j_{23} is near 0.035–0.040 cm^{-1}, and the value of 0.038 cm^{-1} was found the most optimal in subsequent simulations. Then we varied j_{12} while keeping the j_{12}–j_{23} difference constant, and obtained the best agreement for the (j_{12}, j_{13}, j_{23}) values of (0.055, 0.055, 0.017) cm^{-1}, which give KD1–KD4 energies of 0.0, 3.4, 3.9, and 11.6 cm^{-1} (Figure S43).

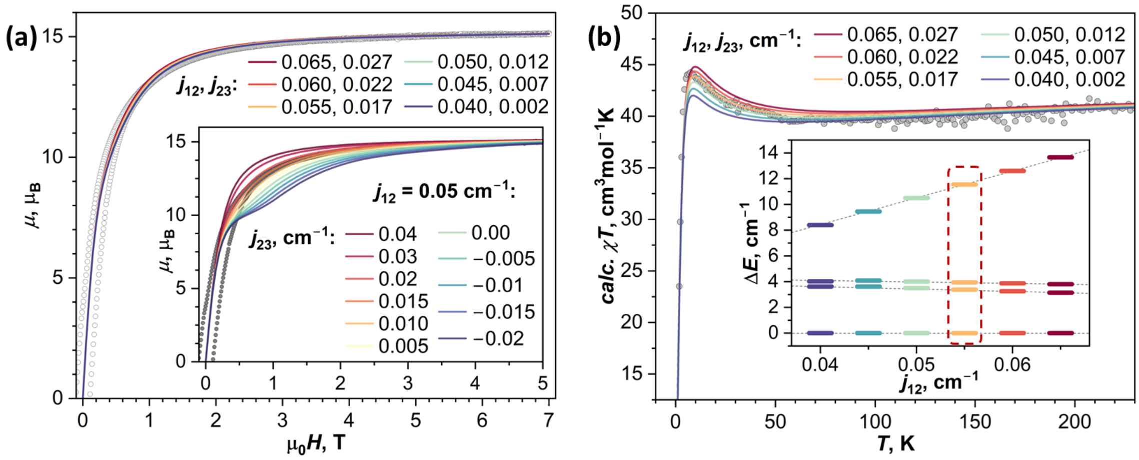

Figure S43. Simulated magnetization curves (a) and χT (b) for **Dy_3-I** with the constant difference $j_{12} - j_{23} = 0.038$ cm^{-1}, whereas individual values of j_{12} and j_{13} are varied. χT is simulated for the magnetic field of 0.5 T, magnetization curve for $T = 1.8$ K; experimental data are shown as gray dots. Inset in (a) shows simulated magnetization curves for j_{12} = 0.05 cm^{-1} and varied j_{23}. Variation of j_{23} with constant j_{12} has a visible effect on magnetization curves and allows estimation that the difference between j_{12} and j_{23} should be around 0.035–0.040 cm^{-1}. Keeping the difference 0.038 cm^{-1} and varying j_{12} has no visible effect on magnetization curves in (a) but changes the shape of χT in (b). The best match to experimental data is obtained for the (j_{12}, j_{13}, j_{23}) values of (0.055, 0.055, 0.017) cm^{-1}.

References

1. Krylov, D. S.; Liu, F.; Avdoshenko, S. M.; Spree, L.; Weise, B.; Waske, A.; Wolter, A. U. B.; Büchner, B.; Popov, A. A. Record-high thermal barrier of the relaxation of magnetization in the nitride clusterfullerene $Dy_2ScN@C_{80}$-I_h. *Chem. Commun.* **2017**, *53*, 7901-7904.
2. Hao, Y.; Velkos, G.; Schiemenz, S.; Rosenkranz, M.; Wang, Y.; Büchner, B.; Avdoshenko, S. M.; Popov, A. A.; Liu, F. Using internal strain and mass to modulate Dy···Dy coupling and relaxation of magnetization in heterobimetallic metallofullerenes $DyM_2N@C_{80}$ and $Dy_2MN@C_{80}$ (M = Sc, Y, La, Lu). *Inorg. Chem. Front.* **2023**, *10*, 468-484.
3. Ye, Q.; Komarov, I. V.; Kirby, A. J.; Jones, M. 3,5,7-Trimethyl-1-azatricyclo[3.3.1.13,7]decan-2-ylidene, an Aminocarbene without π Conjugation. *J. Org. Chem.* **2002**, *67* (26), 9288-9294.
4. Schneider, Y.; Prévost, J.; Gobin, M.; Legault, C. Y. Diazirines as Potent Electrophilic Nitrogen Sources: Application to the Synthesis of Pyrazoles. *Org. Lett.* **2014**, *16* (2), 596-599.
5. Chilton, N. F.; Anderson, R. P.; Turner, L. D.; Soncini, A.; Murray, K. S. PHI: A powerful new program for the analysis of anisotropic monomeric and exchange-coupled polynuclear d- and f-block complexes. *J. Comput. Chem.* **2013**, *34* (13), 1164-1175.
6. Laikov, D. N.; Ustynuk, Y. A. PRIRODA-04: a quantum-chemical program suite. New possibilities in the study of molecular systems with the application of parallel computing. *Russ. Chem. Bull.* **2005**, *54* (3), 820-826.
7. Laikov, D. N. Fast evaluation of density functional exchange-correlation terms using the expansion of the electron density in auxiliary basis sets. *Chem. Phys. Lett.* **1997**, *281*, 151-156.
8. Hafner, J. Ab-initio simulations of materials using VASP: Density-functional theory and beyond. *J. Comput. Chem.* **2008**, *29* (13), 2044-2078.
9. Kresse, G.; Hafner, J. Ab initio molecular dynamics for liquid metals. *Phys. Rev. B* **1993**, *47* (1), 558-561.
10. Kresse, G.; Joubert, D. From ultrasoft pseudopotentials to the projector augmented-wave method. *Phys. Rev. B* **1999**, *59* (3), 1758-1775.
11. Perdew, J. P.; Burke, K.; Ernzerhof, M. Generalized gradient approximation made simple. *Phys. Rev. Lett.* **1996**, *77* (18), 3865-3868.
12. Grimme, S. Density functional theory with London dispersion corrections. *WIREs Comput. Mol. Sci.* **2011**, *1* (2), 211-228.
13. Aquilante, F.; Autschbach, J.; Baiardi, A.; Battaglia, S.; Borin, V. A.; Chibotaru, L. F.; Conti, I.; Vico, L. D.; Delcey, M.; Galván, I. F.; et al. Modern quantum chemistry with [Open]Molcas. *J. Chem. Phys.* **2020**, *152* (21), 214117.
14. Chibotaru, L. F.; Ungur, L. Ab initio calculation of anisotropic magnetic properties of complexes. I. Unique definition of pseudospin Hamiltonians and their derivation. *J. Chem. Phys.* **2012**, *137* (6), 064112.
15. Yang, W.; Barbosa, M. F. d. S.; Israel, N.; Rosenkranz, M.; Liu, F.; Avdoshenko, S. M.; Popov, A. A. Controlling Magnetic Anisotropy of Endohedral Lanthanide Ions by Carbene Addition: Paramagnetic NMR, Lanthanide Luminescence, and Single-Molecule Magnetism in Adamantylidene Adducts of $MSc_2N@C_{80}$ (M = Nd, Dy). *J. Am. Chem. Soc.* **2025**, *147* (37), 33812–33827.
16. Blackmore, W. J. A.; Gransbury, G. K.; Evans, P.; Kragskow, J. G. C.; Mills, D. P.; Chilton, N. F. Characterisation of magnetic relaxation on extremely long timescales. *Phys. Chem. Chem. Phys.* **2023**, *25* (25), 16735-16744.
17. Zorn, R. Logarithmic moments of relaxation time distributions. *J. Chem. Phys.* **2002**, *116* (8), 3204-3209.
